# Patient experiences with hypertrophic cardiomyopathy: a conceptual model of symptoms and impacts on quality of life

**DOI:** 10.1186/s41687-020-00269-8

**Published:** 2020-12-01

**Authors:** Erica Zaiser, Amy J. Sehnert, Ashley Duenas, Sara Saberi, Ella Brookes, Matthew Reaney

**Affiliations:** 1 Evidera, London, UK; 2 MyoKardia, Inc, Brisbane, California USA; 3 Frankel Cardiovascular Center, University of Michigan Hospital, Michigan Medicine, Ann Arbor, Michigan USA; 4 IQVIA, 3 Forbury Place, 23 Forbury Road, Reading, RG1 3JH UK

**Keywords:** Hypertrophic cardiomyopathy, Conceptual model, HCM symptoms, Patient-reported outcomes, Burden of disease, Quality of life, Shortness of breath

## Abstract

**Background:**

Hypertrophic cardiomyopathy (HCM) is a primary myocardial disorder defined by left ventricular hypertrophy that cannot be explained by another cardiac or systemic disease. There is a general lack of knowledge about patients’ perspectives on the symptoms and day-to-day limitations they experience as a result of HCM. We therefore sought an in-depth understanding of patients’ experiences of obstructive (oHCM) and nonobstructive (nHCM) forms of the disease, including symptoms and their quality of life impacts, and to develop a conceptual model to capture them.

**Methods:**

Development of the HCM conceptual model involved a web-based survey to capture patients’ insights, a targeted literature review (which included relevant guidelines and patient advocacy websites), one-to-one interviews with clinical experts, and one-to-one qualitative concept elicitation interviews with patients. Key symptoms and their impacts most important to patients’ experiences were identified and used to develop a conceptual model of the patient experience with HCM.

**Results:**

The HCM symptoms reported by patient interviewees (*n* = 27) were largely consistent with findings from the patient web survey (*n* = 444), literature review, and interviews with three expert clinicians. The symptoms most commonly reported in patient interviews included tiredness (89%), shortness of breath (89%), shortness of breath with physical activity (89%), and dizziness/light-headedness (89%). Other symptoms commonly reported included chest pain (angina) (70%), chest pain (angina) with physical exertion (70%), and palpitations (fluttering or rapid heartbeat) (81%). The most commonly reported impacts of HCM symptoms on patients’ lives included limitations to physical activities (78%), emotional impacts, including feeling anxious or depressed (78%), and impacts on work (63%). Symptoms and impacts were similar for both oHCM and nHCM.

**Conclusions:**

A conceptual model was developed, which identifies the core symptoms that patients with oHCM and nHCM reported as most frequent and most important: shortness of breath, palpitations, fatigue/tiredness, dizziness/light-headedness, and chest pain, as well as the impacts those symptoms have on patients’ lives. This HCM conceptual model reflecting patients’ experiences and perspectives was used in the development of a patient-reported outcomes instrument for use in clinical trials and it may also help inform the clinical management of HCM.

## Background

Hypertrophic cardiomyopathy (HCM) is a primary myocardial disorder defined by left ventricular hypertrophy that cannot be explained by another cardiac or systemic disease [1–4]. Globally, approximately 1/500 people in the general population is thought to be affected by HCM, but the number of diagnosed cases is less than 1/3000 [1, 3, 5]. Patients are often diagnosed at a young age, sometimes even in childhood, and often incur a lifelong and progressive burden from the disease [6, 7].

HCM is a clinically heterogeneous disease, with a diverse clinical presentation and course. It can be a debilitating and life-changing disease resulting in impaired functionality and reduced quality of life [8, 9]. The most commonly reported symptoms are shortness of breath, especially with physical exertion, fatigue, chest pain, palpitations from arrhythmias including atrial flutter or atrial fibrillation, dizziness, and fainting (or syncope) [1, 2, 5, 8, 10, 11].

Because of widely debated historical concerns about elevated sudden cardiac death risk associated with vigorous exercise in athletic individuals [12–15], patients with HCM often limit their physical activity, which can, in turn, lead to other complications, such as obesity and depression [16–20]. Patients with HCM have reduced exercise capacity and cardiorespiratory fitness [21], which have been shown to be independent predictors of early mortality from heart failure and sudden cardiac death as well as disease progression [22–25]. First-line therapies for management of symptomatic HCM have not been shown to improve cardiorespiratory fitness [26]; the only noninvasive measure that has been shown to improve it is moderate-intensity exercise [27]. In symptomatic patients, impacts of HCM such as emotional distress and limited social functioning can lead to impaired quality of life [8, 9].

HCM can be classified as obstructive (oHCM; also known as hypertrophic obstructive cardiomyopathy or HOCM) or nonobstructive (nHCM) based on the presence or absence of left ventricular outflow tract obstruction [1, 2, 11]. It is estimated that two-thirds of patients with HCM have oHCM, and one-third of patients have nHCM [11]. Relatively little is known from the patients’ perspective about the symptoms and day-to-day limitations they experience over time from HCM, and whether experiences differ between oHCM and nHCM.

The objectives of this study were: to gain an in-depth understanding of patients’ experience with oHCM and nHCM, including the symptoms and impacts of the disease that most profoundly affect patients’ quality of life; and to develop a conceptual model capturing the symptoms and impacts of this disease that are most clinically meaningful when considering treatment outcomes. Such a conceptual model is a description or diagram generated to understand patients’ experience with a disease and the relationships among the symptoms and impacts considered in a patient-reported outcome (PRO) instrument, consistent with Food and Drug Administration (FDA) guidance for developing PRO instruments [28]. Symptoms refer to the physical manifestations experienced by patients (e.g. “shortness of breath”), and impacts refer to the consequences those symptoms have on a patient’s life (e.g. “limitations to physical activities”). The conceptual model of a PRO instrument evolves over the course of instrument development as empiric evidence is gathered to support item grouping and scores.

## Methods

Development of a conceptual model to better understand the symptoms and impacts experienced by patients with oHCM and nHCM involved a sequence of steps: a patient web survey to capture their insights; a targeted literature review (which included relevant guidelines and patient advocacy websites); one-to-one interviews with clinical experts; and one-to-one qualitative concept elicitation interviews with patients. All aspects of this study were conducted in accordance with relevant local guidelines for the protection of research participants, and all participants gave written informed consent prior to data collection. This study was conducted according to the Declaration of Helsinki. The qualitative patient interview study protocol was approved by Ethical & Independent Review Services. Elements of this multipart study were also reviewed and approved by the Copernicus Group Independent Review Board (IRB) and the Chesapeake IRB.

### Patient web survey

A patient web-based survey was conducted in 2015 to identify the key signs, symptoms, and impacts of HCM. An 80-question survey was developed in collaboration with the Hypertrophic Cardiomyopathy Association (HCMA), a US-based HCM advocacy, education, and patient-support group, to evaluate patients’ experience of HCM by asking closed questions about the symptoms, treatment decisions and outcomes, and quality of life. The web-based survey was distributed via email to 2469 members of the HCMA. The full web-based survey is available as supplementary material (Supplemental File 1).

### Literature review

A review of published guidelines about HCM diagnosis or treatment was performed to identify HCM-specific symptoms and their impacts. Three HCM guidelines were evaluated to further understand the patient experience: the 2011 American College of Cardiology Foundation/American Heart Association guideline [1], the 2011 Cardiac Society of Australia and New Zealand guidelines [3], and the 2014 European Society of Cardiology guidelines [4].

Secondary research on patient advocacy websites for the HCMA [29] and Cardiomyopathy UK [30] sought to pinpoint any experiences reported on patient forums.

Additionally, a targeted literature review was conducted using PubMed and the Excerpta Medica Database (Embase), coupling “hypertrophic cardiomyopathy” terms with keywords related to outcomes and quality of life. PubMed/Embase search terms were adapted from an existing review and developed with input from a medical research librarian. Published articles were included in the review when they contained details related to symptoms, symptom impacts, effects of therapies on symptoms, quality of life, or PROs reported in adult patients (≥ 18 years of age) with HCM, heart failure, or cardiomyopathy. Articles describing PRO findings were targeted to best reflect patients’ self-reported experiences with the disease, as opposed to relying on clinical outcomes or measurements or physician-reported assessments. Effects of therapies on symptoms were included to help reflect the complete patient experience with HCM, including the fact that some symptoms may be managed by treatment more easily than others. The symptoms and impacts reported in the literature review and the websites were cross-referenced with those identified in the guideline search, and a list of HCM symptoms and impacts was generated. The full literature review methodology and findings are available as supplementary material (Supplemental File 2).

### Clinical expert interviews

Following the patient web survey and literature review, separate, semi-structured, one-to-one telephone interviews with each of three clinical experts from the US and Europe were carried out between December 22, 2016 and January 4, 2017 to verify and build upon the findings of the literature review. The three clinicians were selected because they are recognized experts in cardiology, specifically in HCM patient care and research, and have experience that is largely representative of European and US clinicians working with HCM patients.

Each one-time interview was based on a brief, semi-structured guide, which included asking the clinician to describe aspects of the disease observed in clinical practice described in the clinician’s own words. The clinicians were asked to provide information on how they diagnose HCM, and to detail their experiences with the clinical presentation of the disease, including the most frequently reported symptoms and the impacts these have on patients’ lives. Interviewers asked follow-up questions as appropriate, and probed the clinicians regarding specific symptoms and impacts if these had not been mentioned spontaneously.

### Patient concept elicitation interviews

One-to-one qualitative concept elicitation interviews with 27 patients in the US, the United Kingdom (UK), France, and Italy were conducted in 2017 and 2018 to confirm or add to the findings from the web survey, literature review, and expert clinician interviews. Participants were recruited through referrals from established physician and nursing panels and through patient advocacy organizations. Each participant’s physician was contacted to complete a clinical form detailing the participant’s HCM diagnosis and treatment.

Participants who met the following inclusion criteria were invited to take part in these concept elicitation interviews: male or female patients between 18 and 75 years of age with a documented clinical diagnosis of HCM (hypertrophied and non-dilated left ventricle in absence of systemic or other known cause); New York Heart Association (NYHA) functional class > I; have experienced at least one HCM symptom (e.g. shortness of breath, chest pain, tiredness) within the 6 months prior to enrollment; willing and able to provide written informed consent to participate in research; able to read, speak, and understand English, French, or Italian sufficiently to complete all assessments; and willing and able to participate in a telephone interview session, including adherence to the interview instructions and completion of questionnaires. Patients were excluded if they had a major health problem that complicates their HCM (e.g. severe pulmonary disease or highly symptomatic severe disease other than HCM); were hospitalized for any reason at time of study enrollment; had major surgery, including thoracic or cardiac procedures within 8 weeks prior to enrollment; had a history of obstructive coronary artery disease (i.e. one or more stenoses > 70% of luminal diameter); or had undergone interventions such as myectomy or septal alcohol ablation.

Interviews were conducted in patients’ local languages and consisted of concept elicitation about the key symptoms and impacts of HCM on patients’ daily lives, with the aim of understanding the language patients use to talk about these experiences. A semi-structured interview guide was used to maintain consistency across different interviewers. Interviewees were first asked to spontaneously describe the process of diagnosis, symptomatology at the time of diagnosis, and treatment history. Subsequently, interviewers further probed the participants about their experience using a list of signs, symptoms, and impacts of HCM derived from the patient web survey, literature review, and clinician interviews. For each sign, symptom, and impact reported, participants were asked about the timing, frequency, location, triggers, sources of relief, and level of disturbance to their life. Each patient was asked to identify the five “most important” HCM symptoms and why those symptoms were chosen as most important.

All interviews were audio-recorded and transcribed, and non-English language (i.e. French or Italian) transcripts were translated into English for further analysis. All transcripts were cleaned of any personal identifiable information prior to coding. Qualitative interview data were analyzed using ATLAS.ti™ software version 7.5 (ATLAS.ti Scientific Software Development GmbH, Berlin, Germany). Key concepts identified from the interview transcripts were translated into relevant codes and used to maintain a coding dictionary and a grid to assess concept saturation: the point at which further interviews stop identifying new concepts. Assessing content saturation ensures that a thorough and comprehensive list of concepts has been identified, and is separate from assessing the frequency and importance of each concept.

A database was developed, tested, and validated to hold the quantitative sociodemographic information provided prior to and during the interview, and a quantitative list of signs, symptoms, and impacts mentioned by patients during the interviews. Data were entered into the database and reviewed by project scientific staff. Tables summarizing the data were developed and populated. Means, standard deviations, and ranges were calculated for continuous variables, and frequencies and percentages were calculated for categorical values. A two-sample t-test was used to compare the mean number of symptoms reported between patients with oHCM and nHCM.

### Conceptual model development

Participant-reported HCM symptoms and impacts were compared with those identified in the literature and clinician interviews. The key concepts identified as underlying the patient experience contributed to the development of a conceptual model of the most relevant HCM symptoms and impacts that addresses both obstructive and nonobstructive forms of the disease.

## Results

### Patient web survey

A total of 3089 adult patients were invited to complete the survey, with 2469 surveys successfully delivered. Of these, 474 responses to the survey were received, of which 444 responses (94%) were complete, self-reported an HCM diagnosis, and were analyzed. Among all respondents, 58.0% reported a diagnosis of oHCM (i.e. “have you been diagnosed with left ventricular outflow tract obstruction?”), 33.1% reported a diagnosis of nHCM, and 8.8% did not know their specific HCM condition. Overall, fatigue (74%), shortness of breath upon exertion (73%), and light-headedness (70%) were reported as the symptoms experienced most often. Other symptoms reported by more than half the respondents included palpitations (54%), dizziness after exertion (54%), and exercise intolerance (57%). Chest pain was reported by 39% of respondents, and fainting by 24%.

When asked about the impact of their symptoms on physical activity, 21% reported no limitation, 42% reported slight limitation, 31% reported marked limitation, and 6% reported being unable to be physically active without discomfort.

Patients with oHCM reported experiencing a greater number of symptoms than did patients with nHCM: 84% of oHCM patients reported experiencing four or more symptoms characteristic of HCM, versus 55% of nHCM patients. When asked to define the severity of their symptoms using descriptions based on the NYHA functional classification scale, 43% of patients with oHCM reported moderate-to-severe symptoms (i.e. NYHA Class III or IV), compared with 27% of nHCM patients. About 70% of patients with oHCM reported that their symptoms had somewhat or significantly worsened since their diagnosis, and that their symptoms had a greater impact on their ability to work.

The time between first experiencing symptoms and being diagnosed with HCM was reported as < 3 months by 36.5% of respondents, 3 months to 1 year by 14.1%, 1–3 years by 11.2%, 3–5 years by 6.6%, and > 5 years by 18.7%; 12.9% of respondents reported having never experienced symptoms.

### Literature review

The literature search yielded a total of 256 abstracts and titles for potential consideration. A total of 220 studies were excluded; the main reasons for exclusion were irrelevant patient population or no mention of PROs, symptoms, or impacts. After full-text review of the remaining 36 articles, 28 studies were excluded; the main reason for exclusion was no mention of PROs. The final set of eight articles included for review comprised five cross-sectional studies, one qualitative study, one cohort study, and one cost-effectiveness study.

The articles describing the experiences of patients with HCM identified in the literature review revealed shortness of breath or dyspnea, chest pain (angina) [31, 32], and fainting (syncope) [31–33] to be the primary complaints/symptoms of patients with HCM.

Similarly, all three HCM professional guideline documents describe patients with HCM as having common symptoms of dyspnea, palpitations, chest pain (angina), and fainting (syncope) [1, 3, 4]. Furthermore, the literature review identified fatigue [4], tachyarrhythmia [32, 33], orthopnea [9], pulmonary congestion [1], excessive sweating [4], and heart failure [4, 33] as additional symptoms experienced by some patients with HCM.

The patient advocacy websites also indicated that symptoms such as chest pain, light-headedness, blackouts/dizziness, and fatigue were common in HCM [29, 30]. In addition, the websites noted arrhythmia, cardiac arrest, and endocarditis as other symptoms experienced by some patients with HCM [29, 30].

These symptoms identified by the literature review were considered highly impactful on the mental health, physical functioning, and overall quality of life of patients [16, 18, 32–35]. Depression was also commonly reported in several studies [32, 33] and on the patient advocacy websites [29, 30]. The most commonly reported impact measured in the literature review was anxiety, which was mentioned in four studies [33–35]. It was also noted on the patient advocacy websites [29, 30].

Overall, the review was consistent in identifying shortness of breath/dyspnea, light-headedness and blackouts/fainting (syncope), palpitations/tachycardia, fatigue, and dizziness as the most common symptoms among patients with HCM. Furthermore, anxiety, depression, and reduced physical functioning with shortness of breath were identified as being particularly impactful on patient quality of life and functioning.

Although the presence of left ventricular outflow tract obstruction (oHCM) was associated with more severe symptoms and greater risk of heart failure and cardiovascular death [2], there was limited information in the literature describing differences in symptom experiences between patients with oHCM and those with nHCM. This topic was explored further in the clinician and patient interviews.

### Clinical expert interviews

The clinical expert interviews showed the three clinicians to be highly consistent in their views of the symptom burden of HCM and how these symptoms impact patients’ lives. They reported that symptoms are experienced by most patients diagnosed with HCM, particularly those with oHCM, and that the most commonly reported symptoms were shortness of breath with exertion or after a meal, chest pain (angina), palpitations, and feeling faint (syncope) or dizzy. The three clinical experts reported that the symptoms of HCM are often nonspecific, overlap with one another, can vary from day to day, and can show similarities with side effects of treatment or symptoms of a comorbidity. Because of these challenges, they said that symptoms alone are rarely used to diagnose HCM in their practices, and physical and imaging assessments are required.

When asked to what extent the most frequently reported symptoms were consistent with their assessment of the cardinal symptoms of HCM, all clinicians referred to the difficulty in untangling the symptoms of HCM from treatment side effects or comorbid issues such as obesity. For example, fatigue, hypertension, and sexual impairment were listed as symptoms that could be due to treatment, and shortness of breath was listed as a symptom that could also be due to excess weight.

Of these commonly experienced symptoms, shortness of breath and dizziness/light-headedness were considered by two of the three clinical experts as the most bothersome to patients. Chest pain (angina), feeling faint (syncope), tiredness/fatigue, and palpitations were also ranked by one of the three clinical experts as among the most bothersome for patients. Exertion was considered to be the predominant trigger for symptoms (with the exception of chest pain [angina], which can occur spontaneously). Limitations to physical activity were considered to have the greatest impact on patients’ lives, and the clinicians reported that many patients avoid exercise out of fear of sudden death. The experts cited anxiety as the most common psychological impact related to patients’ HCM symptom burden.

The clinicians were asked to describe how the experience of patients with oHCM and nHCM are similar or different, and their responses are summarized in Table 1. The clinicians reported that, in general, patients with oHCM experience very similar symptoms to patients with nHCM, but patients with oHCM may experience more symptoms simultaneously, more severe symptoms, and more consistent and sustained symptoms than do patients with nHCM.

**Table 1:** Clinical similarities and differences between oHCM and nHCM as reported by clinical experts

Are there differences between oHCM and nHCM with regard to:	Clinician 1 (Italy)	Clinician 2 (US)	Clinician 3 (France)
**Number of symptoms?**	• Obstructive patients have more reproducible and constant symptoms	• Obstructive patients have more symptoms	• (Clinician did not provide answer to this question directly)
**Types of symptoms?**	• Obstructive patients experience palpitations and syncope after effort (recovery phase); not as typical for nonobstructive patients • Syncope on effort is rare, and a worrying sign of severity and instability	• Obstructive patients experience more light-headedness	• Obstructive patients have more frequent shortness of breath with exercise, dyspnea, and angina • Dizziness and palpitations are also more likely with obstruction
**Severity of symptoms?**	• Obstructive patients experience more severe symptoms	• Obstructive patients perhaps experience more severe symptoms • Symptoms show up earlier in the disease course so they progress more than in nonobstructive patients	• More severe with obstruction
**Frequency of symptoms?**	• Patients with oHCM have more frequent and reproducible symptoms than those with nHCM • Non-obstructed patients are much more variable and difficult to reproduce symptoms in	• Not really; once symptoms show up, they are there	• More frequent with obstruction
**Impacts of symptoms?**	• Obstructive patients have more symptoms, more severe symptoms, and are more consistently symptomatic • When someone nonobstructive gets progressive symptoms, this is harder to deal with because it is harder to treat	• Patients with more symptoms and those more functionally disabled tend to be more depressed, so perhaps a greater proportion of patients with obstructive disease are depressed because they tend to have more severe symptoms earlier in the disease	• Symptoms are nonspecific so you must rely on more solid parameters (degree of thickness and obstruction, fibrosis, and arrhythmias), but because obstruction can lead to more severe symptoms, it can lead to more impacts
**Psychological impact of HCM?**	• All clinicians stated that there were psychological impacts associated with HCM and that it was most common for patients to have anxiety, especially after the initial diagnosis • Generally, the clinicians thought that obstructive patients experienced a greater psychological impact as a result of the greater severity of their symptoms compared with nonobstructive patients • Overall, clinicians perceived the patients with the most severe symptoms as more likely to experience a psychological impact		

*HCM* Hypertrophic cardiomyopathy, *nHCM* Nonobstructive HCM, *oHCM* Obstructive HCM, *US* United States of America

### Patient concept elicitation interviews

Concept elicitation interviews were performed with a total of 27 patients with HCM. Each concept elicitation interview was performed over approximately 90 min. Demographic and clinical information for the 27 interviewed patients is summarized in Table 2. The mean age of the patients was 44.6 years, and the most common comorbid conditions were anxiety (40.7%), hypertension (29.6%), and depression (25.9%). Patients rated the severity of their HCM symptoms that day as very mild (15.4%), mild (30.8%), moderate (46.2%), severe (3.8%), or very severe (3.8%). Clinical confirmation of obstructive status was missing for seven patients; four of these patients self-reported oHCM diagnoses, and self-reported obstructive status was unknown/missing for three patients.

**Table 2:** Demographic and clinical characteristics of concept elicitation patient interviewees

Characteristic	Patients (***N*** = 27)
Sex, male, n (%)	11 (40.7)
Age, yrs, mean (SD) [range]	44.6 (15.01) [22–74]
Age first diagnosed with HCM, yrs, mean (SD); median [range]	32.2 (17.11); 27 [0–72]
**Country, n (%)**	
UK	8 (38.1)
France	6 (22.2)
Italy	7 (25.9)
US	6 (22.2)
**Employment status, n (%)** **(patient-reported)**	
Employed, full-time	12 (44.4)
Employed, part-time	5 (18.5)
Homemaker	1 (3.7)
Student	1 (3.7)
Retired	5 (18.5)
Disabled	1 (3.7)
Other^a^	2 (7.4)
**Is HCM the subject’s primary cardiovascular diagnosis? (clinician-reported)**	
Yes, n (%)	22 (81.5)
**How long has the subject been diagnosed with HCM? (clinician-reported)**	
Yrs, mean (SD)	9.3 (8.24)
**HCM obstruction status, n (%) (clinician- or patient-reported)**^b^	
Obstructive^b^	11 (40.7)
Nonobstructive	13 (48.1)
Missing	3 (11.1)
**Health conditions, n (%)**^**c**^ **(patient-reported)**	
None	7 (25.9)
Anemia	5 (18.5)
Angina	5 (18.5)
Anxiety	11 (40.7)
Arthritis	2 (7.4)
Cancer	2 (7.4)
COPD/emphysema	1 (3.7)
Depression	7 (25.9)
Diabetes	1 (3.7)
Hypertension	8 (29.6)
Myocardial infarction	2 (7.4)
Atrial fibrillation	5 (18.5)
**Severity of HCM symptoms as rated “today”, n (%) (patient-reported)**	
Very mild	4 (15.4)
Mild	8 (30.8)
Moderate	12 (46.2)
Severe	1 (3.8)
Very severe	1 (3.8)
**Overall health, n (%) (patient-reported)**	
Excellent	2 (7.4)
Very good	2 (7.4)
Good	17 (63.0)
Fair	5 (18.5)
Poor	1 (3.7)

*COPD* Chronic obstructive pulmonary disease, *HCM* Hypertrophic cardiomyopathy, *SD* Standard deviation, *UK* United Kingdom, *US* United States, *yrs* Years

^a^Other included self-employed/PhD student, and craftsman

^b^Obstructive HCM was clinician-confirmed for seven patients and self-reported by four additional patients; all 11 were considered to have obstructive HCM for the qualitative analyses

^c^Not mutually exclusive

Table 3 summarizes the frequency of HCM symptoms reported by interview participants. A total of 29 different symptoms were reported and complete content saturation (the point at which further interviews stop identifying new concepts) was reached after 23 interviews. The most commonly reported symptoms included tiredness (89%), shortness of breath (89%), shortness of breath with physical activity (89%), and dizziness/light-headedness (89%). Other symptoms commonly reported included chest pain (angina) (70%), chest pain (angina) with physical exertion (70%), and palpitations (fluttering or rapid heartbeat) (81%).

**Table 3:** Frequency of HCM symptoms reported by at least two patients in concept elicitation patient interviews

HCM symptom	Patients (***N*** = 27) n (%)
Shortness of breath (dyspnea)	24 (89)
Shortness of breath when lying flat or at rest	14 (52)
Shortness of breath after meals	15 (56)
Shortness of breath with physical activity	24 (89)
Tiredness	24 (89)
Chest discomfort	11 (41)
Chest pain (angina)	19 (70)
Chest pain with physical activity/exertion	19 (70)
Chest pain after meals	9 (33)
Dizzy/light-headed	24 (89)
Fainting	11 (41)
Palpitations/heart beating quickly/heart fluttering/extrasystole/tachycardia	22 (81)
Sweating	2 (7)
“Couldn’t stand very well” (trouble standing)	2 (7)
Low heart rate	2 (7)
Overheating	2 (7)
Nausea	5 (19)
Headaches	2 (7)

*HCM* Hypertrophic cardiomyopathy

Participants with oHCM reported all the same major symptoms as those with nHCM. Among the oHCM participants, 20 symptoms were reported, of which 85% were reported in the first four interviews. Complete concept saturation in this sample was reached after nine interviews. Among the nHCM participants, 24 symptoms were reported; 88% of these were reported in the first 10 interviews. Concept saturation in this sample was reached after 13 interviews. The mean (standard deviation) number of symptoms reported did not vary between the two patient groups, with oHCM patients having 10.0 (1.76) versus nHCM patients having 8.8 (2.68, *p* = 0.24).

Table 4 lists the symptoms identified as most important to patients. The question was intentionally left open-ended for participants to consider factors that they deem important. Shortness of breath (81% overall; 91% oHCM; 69% nHCM) was the symptom most commonly reported as important, followed by tiredness (67% overall; 55% oHCM; 85% nHCM), palpitations (67% overall; 73% oHCM; 62% nHCM), and chest pain (56% overall; 64% oHCM; 46% nHCM). Two participants (10%) did not rate the importance of their symptoms.

**Table 4:** Symptoms identified as most important to patients during the concept elicitation interviews

HCM symptom, n (%)	Patients (***N*** = 27)^**a**^
Shortness of breath (dyspnea)	22 (81)
Tiredness	18 (67)
Palpitations/heart beating quickly/heart fluttering/extrasystole/tachycardia	18 (67)
Chest pain (angina)	15 (56)
Dizzy/light-headed	13 (48)
Fainting	3 (11)
Shortness of breath after meals	2 (7)
Shortness of breath with physical activity	2 (7)
Chest discomfort	2 (7)
Nausea	2 (7)
Chest pain after meals	1 (4)
Sweating	1 (4)
Overheating	1 (4)
Feet swelling	1 (4)

*HCM* Hypertrophic cardiomyopathy

^a^Two nHCM patients did not provide a list of symptoms; the obstruction status of three patients was unknown

Table 5 describes impacts of the disease on aspects of the patients’ lives identified during the concept elicitation interviews. A total of 15 impact concepts were identified. Concept saturation was achieved within the first 23 interviews. The most commonly reported impacts included limitations to physical activities (78%), emotional impacts (78%), feeling anxious or depressed (78%), and impacts on work (63%).

**Table 5:** Impacts of the disease most frequently identified by patients during the concept elicitation interviews

Impact of HCM, n (%)	Patients (***N*** = 27)
Limitations to physical activities	21 (78)
Emotional impacts	21 (78)
Feeling anxious or depressed	21 (78)
Work	17 (63)
Family	16 (59)
Social life	16 (59)
Limitations to daily tasks	14 (52)
Household chores	13 (48)
Sleep disruption	12 (44)
Driving	1 (4)
Communication	1 (4)
Can’t get plastic surgery	1 (4)
Traveling	1 (4)
Needs to have recovery time after activities	1 (4)
Can’t take certain cold medicines	1 (4)

*HCM* Hypertrophic cardiomyopathy

### Conceptual model development

The HCM symptoms reported by patient interview participants were largely consistent with the findings from the patient web survey, the literature review, and the interviews with the expert clinicians. The patient interviews provided additional specifics on the frequency and importance of these symptoms, and the impact they have on patients’ lives.

**Fig. 1:**
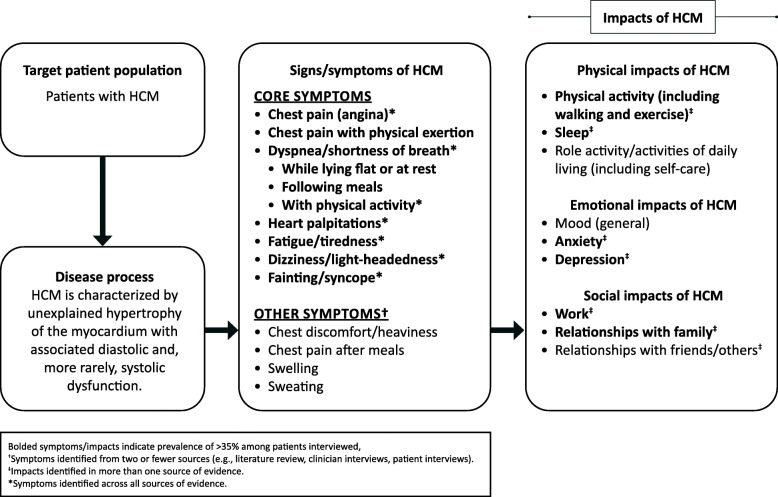
A conceptual model of the patient experience with hypertrophic cardiomyopathy

A conceptual model that organizes and prioritizes the most relevant concepts (both symptoms and impacts) in the HCM patient population was developed (Fig. 1). The conceptual model identifies shortness of breath, palpitations, fatigue/tiredness, dizziness/light-headedness, and chest pain as the core symptoms of both oHCM and nHCM. These symptoms have physical, emotional, and social impacts on patients, including limitations on their activities of daily living.

## Discussion

This comprehensive approach to identifying the most relevant concepts (symptoms and their impacts) in HCM generated a conceptual model of the patient experience with the disease. The conceptual model is based on multiple sources of evidence (literature review, including relevant guidelines and patient advocacy websites; clinical expert interviews; patient web survey; and patient concept elicitation interviews) and indicates a substantial patient burden associated with HCM and its symptoms. This is the first study to describe a conceptual model specific to patients with this important disease.

The model identifies shortness of breath, palpitations, fatigue/tiredness, dizziness/light-headedness, and chest pain as the core symptoms of both oHCM and nHCM. The model also identifies limitations to physical activities, emotional impacts of feeling anxious or depressed, and impacts on work, sleep, and family relationships as the key impacts of HCM on areas of patients’ lives. These HCM symptoms and impacts should be considered in clinical assessments of disease severity and progression. Given that therapies are aimed at reducing the burden of symptoms, thereby improving quality of life along with improving outcomes, response to currently available and any future treatments for HCM in terms of these core symptoms and their impacts should be further investigated.

Symptoms and impacts are similar for oHCM and nHCM, and although some differences emerge between the two forms (e.g. number and severity of HCM symptoms, heterogeneous disease progression), the conceptual model presented here is appropriate for both.

Historically, therapy has focused on relieving symptoms, and patients with HCM are often treated with drugs indicated for broader cardiovascular disorders, such as heart failure (e.g. beta-blockers, calcium channel blockers, or disopyramide). Pharmacologic management options for HCM recommended by current guidelines produce limited and variable improvement in patients’ symptoms and functional status. The development of this HCM-specific conceptual model reflects the core symptoms and impacts of this disease from the perspectives of patients and clinical experts; it is expected to help in the management of patients’ symptoms by formalizing patient experience as a reference for clinicians to use for assessment of disease burden and progression. Full awareness of core symptoms and their impact on quality of life is key to shared decision-making with regard to treatment options. Such a conceptual model can further provide a basis for a PRO instrument to evaluate treatment efficacy for investigational drugs in clinical trials and in clinical practice.

Strengths of this study include the facts that a combination of qualitative and quantitative approaches was applied, clinicians and patients from multiple countries were included, and consistent and similar findings emerged across the various sources consulted. Despite being applied to a rare disease, this study generated a large, meaningful dataset that reflects diverse patient experiences. Limitations of this study include the relatively small number of clinical experts and patients interviewed (a factor related to the disease’s rarity) and the fact that some patients’ obstructive status (i.e. oHCM or nHCM) was unknown or self-reported without clinical confirmation of that diagnosis.

## Conclusions

A conceptual model was developed that identifies the core symptoms of HCM (those that patients reported as most frequent and most important: shortness of breath, palpitations, fatigue/tiredness, dizziness/light-headedness, and chest pain), as well as the impacts those symptoms have on patients’ lives (including limitations to physical activities, emotional impacts, and impacts on work, sleep, and family relationships). In accordance with recommended guidelines for the development of PRO instruments [28], the symptoms and impacts of HCM presented in this conceptual model can be used as a framework for developing a targeted PRO instrument that reflects the patient experience with HCM, and that would be useful for assessing outcomes in clinical practice or clinical trials. Such a PRO instrument can measure the impact of an intervention on one or more aspects (concepts) of patients’ health status, which include purely symptomatic responses, more complex concepts (e.g. ability to carry out activities of daily living), and extremely complex concepts (e.g. quality of life, which is widely understood to be a multidomain concept with physical, psychological, and social components). Data generated with a PRO instrument can provide evidence of a treatment benefit from patients’ perspectives [28].

## Supplementary Information

**Additional file 1.**

**Additional file 2.**

## Data Availability

The datasets used and analyzed for the current study are available from the corresponding author upon reasonable request.

## Abbreviations

COPD: Chronic obstructive pulmonary disease
HCM: Hypertrophic cardiomyopathy
HCMA: Hypertrophic Cardiomyopathy Association
nHCM: Nonobstructive HCM
NYHA: New York Heart Association
oHCM: Obstructive HCM
PRO: Patient-reported outcome
SD: Standard deviation
UK: United Kingdom
US: United States of America
yrs: Years
