# Supplementary material for: Patient experiences with hypertrophic cardiomyopathy: a conceptual model of symptoms and impacts on quality of life
Source: J Patient Rep Outcomes. 2020 Dec 1;4:102. doi: 10.1186/s41687-020-00269-8 (PMC7708573; doi:10.1186/s41687-020-00269-8)

Survey Results and Analysis for Patient Journey Questionnaire 2015 – HCMA and MyoKardia

January 22, 2016

MyoKardia

Survey background

- Collaboration between Lisa Salberg (HCMA) and MyoKardia
- 80 question, web-based survey, exploring HCM patient journey, developed by Lisa Salberg and MyoKardia
- Fielded between August 18 and October 22 of 2015
- The survey was sent online to HCMA members who had completed previous surveys in the past 2 years.
  - 3089 invited
  - 2469 successfully delivered
  - 71 bounces
  - 633 clicks
  - 474 completed
  - 444 completed and answered “yes” to question 1 *

* Question 1: “Do you have hypertrophic cardiomyopathy (HCM)? Please note that this includes those who are genetically positive or have a diagnosis either with or without symptoms. Parents may complete on behalf of children under 18 with HCM. If you have lost a loved one to HCM - please answer "no" on this survey and do not answer on their behalf.”

Q2 **At what age were you first diagnosed with HCM?**

Response	Count	Percent
Under 12 months	6	1.4%
1 - 5 years	7	1.6%
6-12 years	17	3.8%
13-25 years	42	9.5%
26-40 years	111	25.0%
41-55 years	174	39.2%
56-70 years	80	18.0%
71 years or older	7	1.6%
Total	444	

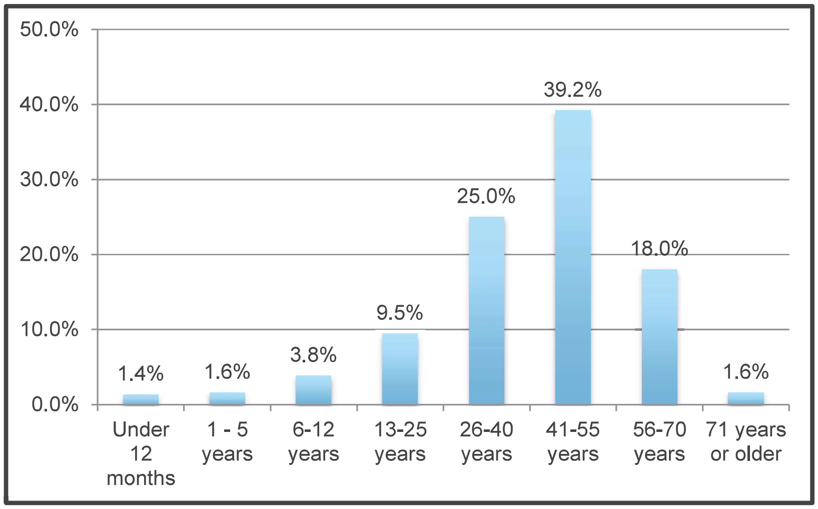

MyoKardia I CONFIDENTIAL
MyoKardia

Q3 **What was the reason for your diagnosis?**

Response	Count	Percent
My symptoms raised a concern	206	47.1%
No symptoms. Found at general physical - either from a heart murmur-- abnormal ECG or conversation with doctor	116	26.5%
Found at family screening due to a relative's diagnosis of HCM	61	14.0%
Other	48	11.0%
Found during athletic screening	6	1.4%
Total	437	

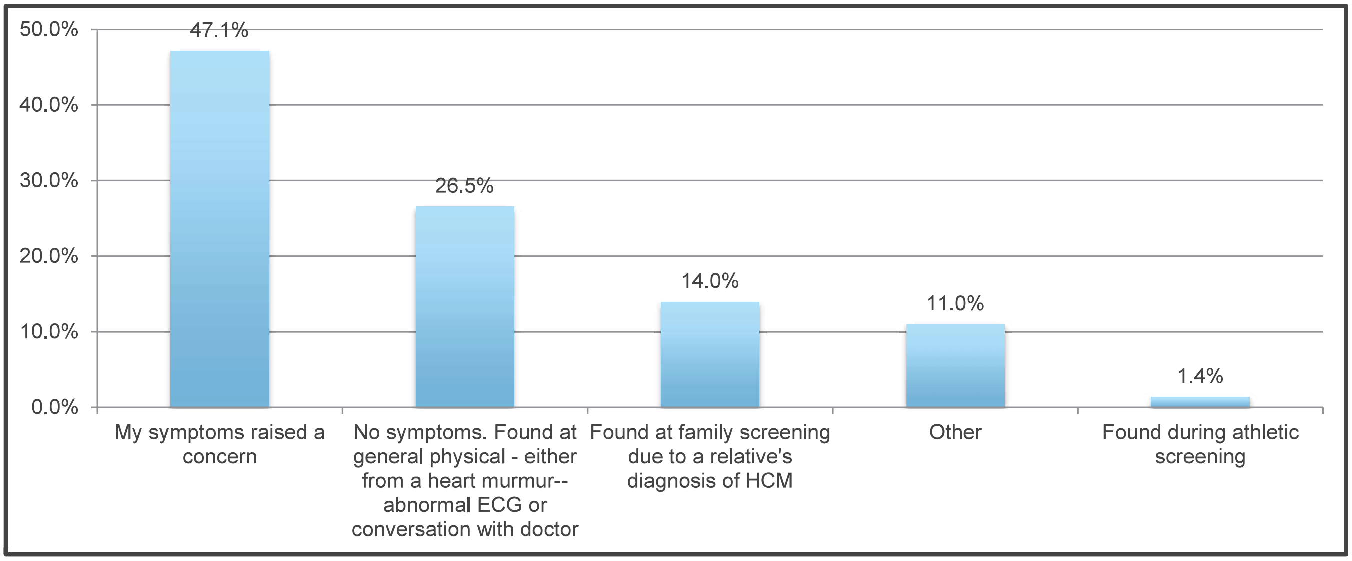

Q3A1 **NEW: Redefine categories**
What was the reason for your diagnosis?

Response	Count	Percent
Symptoms	206	46.4%
Unrelated medical investigation	122	27.5%
Screening	61	13.7%
Other	55	12.4%
Total	444	

Q3A2 **NEW: Reclassify "Other" into existing categories**
What was the reason for your diagnosis?

Response	Count	Percent
Symptoms	239	53.8%
Unrelated medical investigation	138	31.1%
Screening	67	15.1%
Total	444	

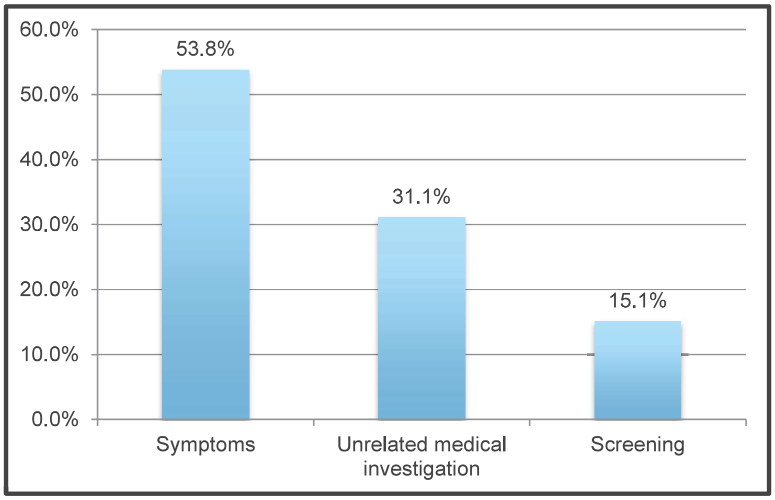

Q4 **Prior to your diagnosis of HCM-- were you diagnosed with any of the following conditions? (Check all that apply)**

Response	Count	Percent
Innocent murmur	192	43.2%
Anxiety or panic attacks	71	16.0%
Mitral valve prolapse	67	15.1%
Depression	67	15.1%
Asthma	66	14.9%
Other	55	12.4%
Total (N): all respondents	444	

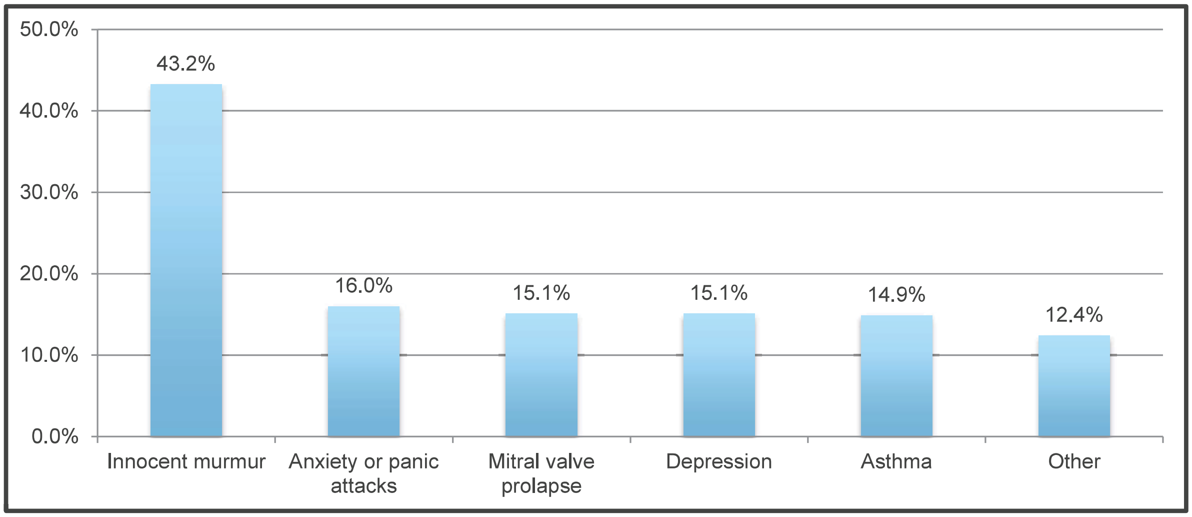

Q5 **Who first diagnosed you with HCM? (Please select one)**

Response	Count	Percent
General cardiologist	317	72.5%
Cardiologist specializing in HCM	61	14.0%
Primary care physician	42	9.6%
Other	17	3.9%
Total	437	

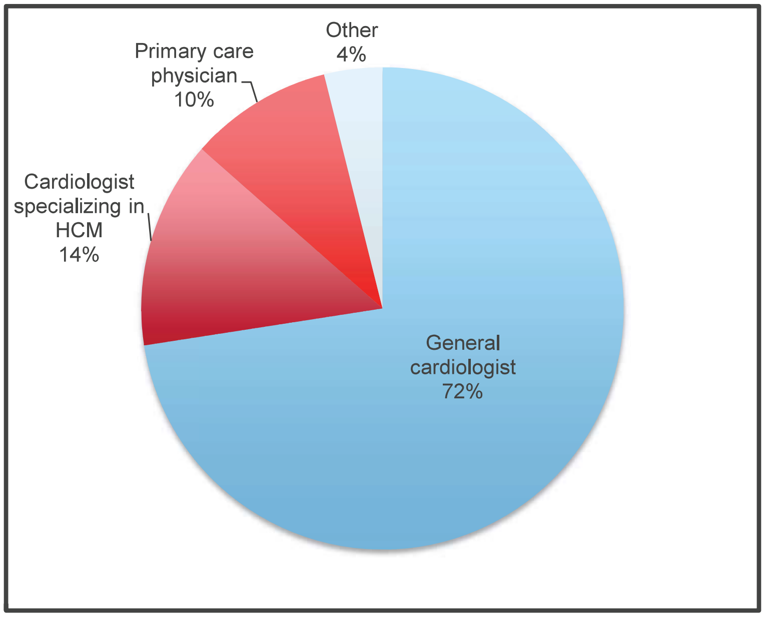

Q6 **Do you have obstructive HCM (have you been diagnosed with left ventricular outflow tract obstruction)?**

Response	Count	Percent
Yes	256	58.0%
No	146	33.1%
Don't know	39	8.8%
Total	441	

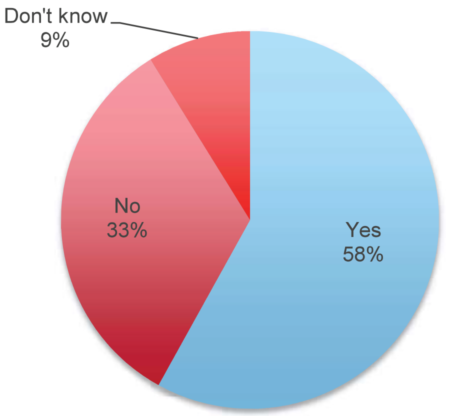

Q7 **Have you been tested for a genetic mutation related to HCM?**

Response	Count	Percent
Yes	247	55.9%
No	195	44.1%
Total	442	

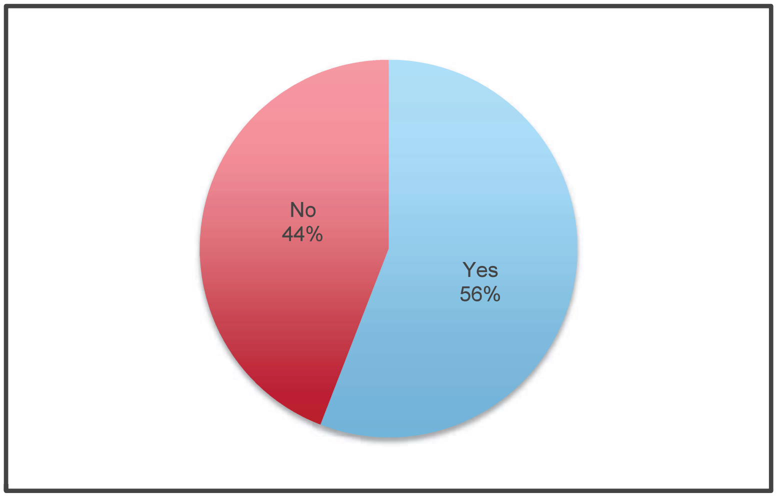

Q8 **If yes-- do you know which mutation was identified?**

Response	Count	Percent
Mutation was not identified	78	34.5%
Don't know it	57	25.2%
MYBPC3 (Cardiac myosin-binding protein c)	33	14.6%
MYH7 (β-cardiac myosin heavy chain)	25	11.1%
TNNT2 (Cardiac troponin T)	7	3.1%
TNNI3 (Cardiac troponin I)	6	2.7%
TPM1 (α-Tropomyosin)	2	0.9%
ACTC (Cardiac actin)	0	0.0%
MYL2 (Cardiac myosin regulatory light chain)	0	0.0%
MYL3 (Cardiac myosin regulatory light chain 3)	4	1.8%
Other	14	6.2%
Total	226	

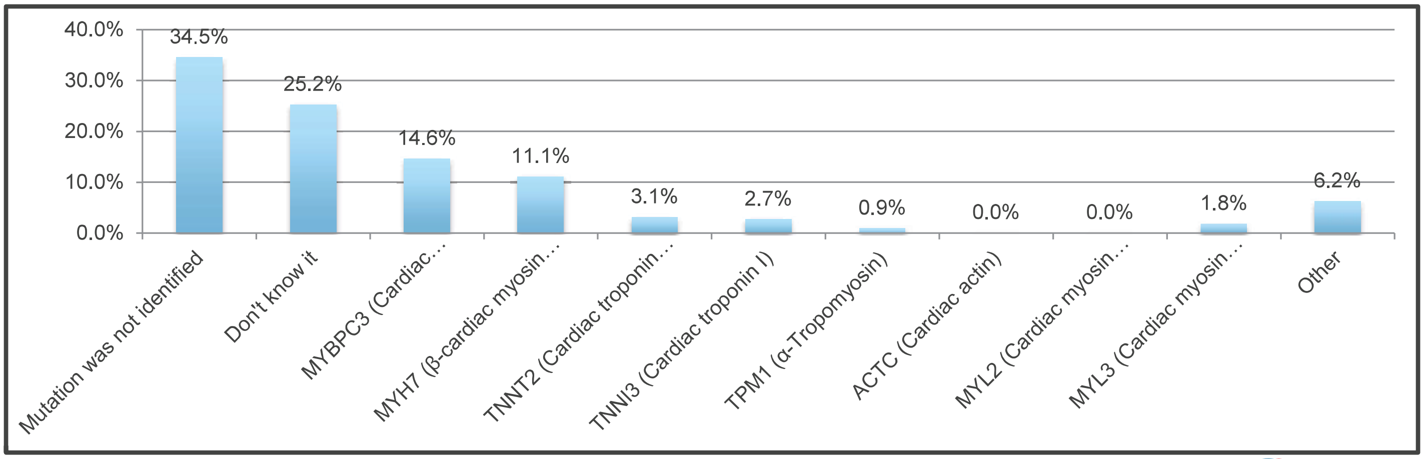

Q9 **If you have not had a genetic test for HCM-- what were the reasons? (Check all that apply)**

Response	Count	Percent
Not offered as an option-- or not discussed with my physician	76	39.0%
Cost is too great	53	27.2%
Benefit of genetic test is not clear	44	22.6%
Not reimbursed by my insurance	40	20.5%
Other	39	20.0%
Concern about stigma of HCM	10	5.1%
My physician recommended against it	3	1.5%
Total (N): those who answered "no" to genetic testing	195	

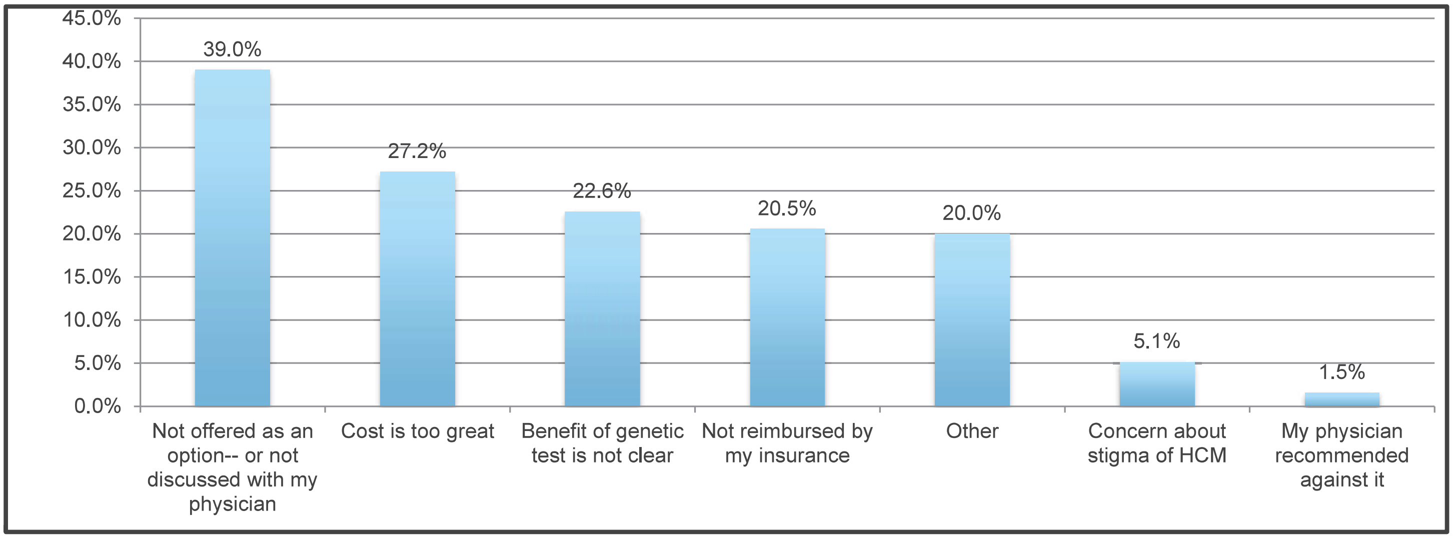

Q10 **Have you received an MRI (imaging test) for your HCM?**

Response	Count	Percent
Yes-- only at diagnosis	145	33.0%
Yes-- only during routine follow-ups	68	15.5%
Yes-- at diagnosis and during routine follow-ups	83	18.9%
No	144	32.7%
Total	440	

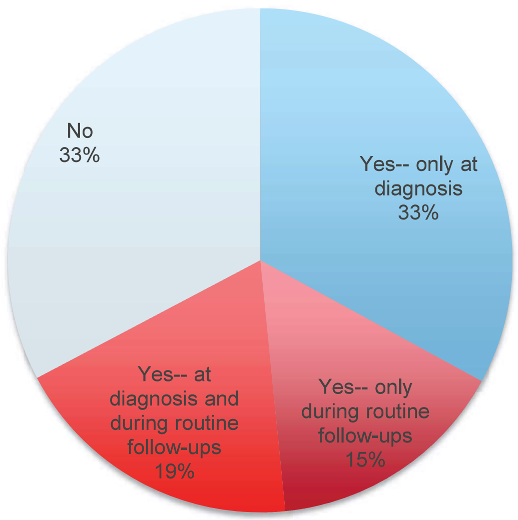

Q11 **Have you undergone exercise testing (walked on a treadmill or stationary bike then gotten an echocardiogram) for your HCM?**

Response	Count	Percent
Yes-- only at diagnosis	105	23.9%
Yes-- only during routine follow-ups	96	21.9%
Yes-- at diagnosis and during routine follow-ups	201	45.8%
No-- I have not had this test-- nor was it discussed	26	5.9%
No-- I have not had this test-- but was told due to my obstruction and gradient it was not necessary.	11	2.5%
Total	439	

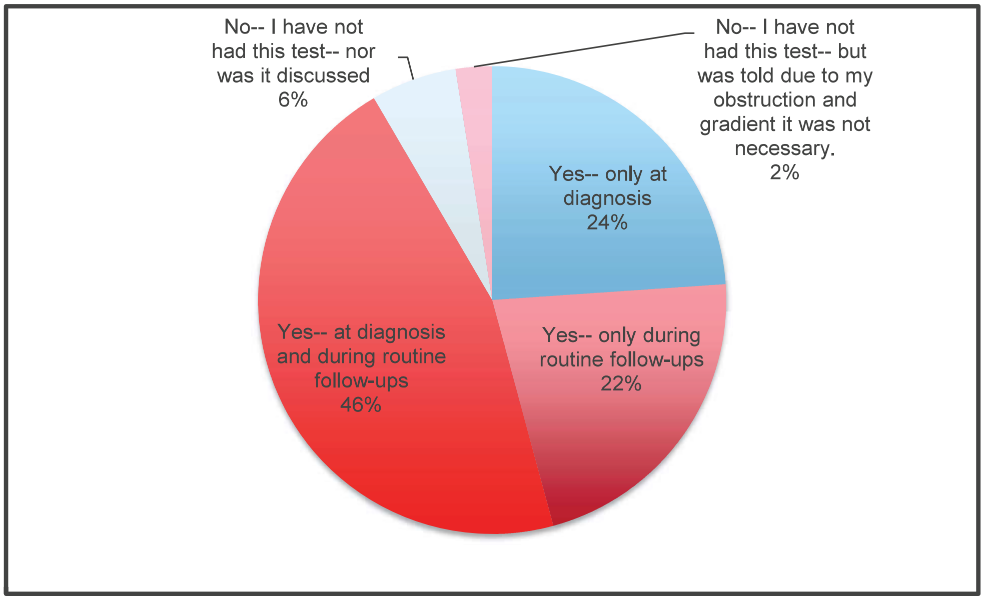

Q12 **Have you ever been diagnosed with any of the following? (Check all that apply)**

Response	Count	Percent
Atrial fibrillation	152	34.2%
TIA or stroke	27	6.1%
Neither	273	61.5%
Total (N): all respondents	444	

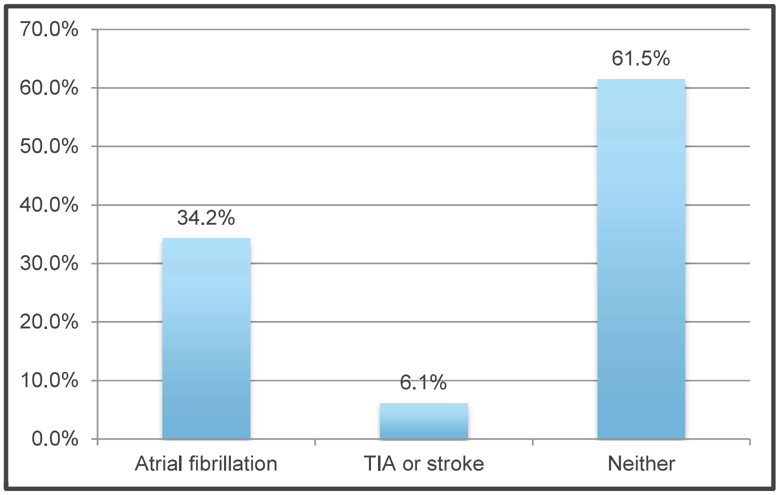

Q13A **NEW: Include only yes responders to Afib** **If you were diagnosed with atrial fibrillation-- how do you define your atrial fibrillation?**

Response	Count	Percent
Only occasional (paroxysmal - comes and goes)	120	78.9%
Present all the time (chronic - all the time)	30	19.7%
Total (N): all respondents stating "yes" to diagnosed with Afib	152	

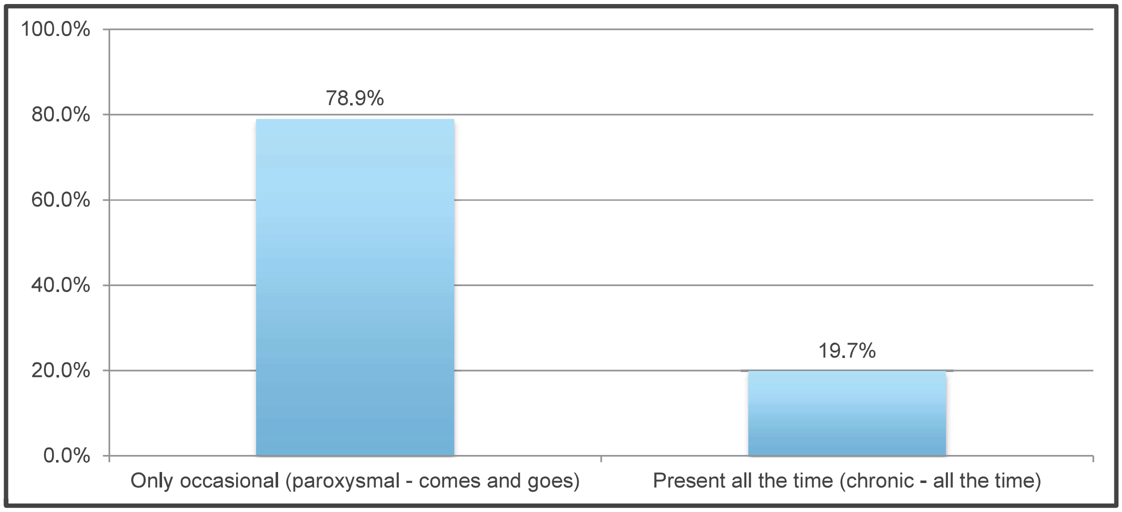

Q14A **NEW: Include only yes responders to Afib** **Have you ever been cardioverted (with defibrillation from an external device) to treat your atrial fibrillation?**

Response	Count	Percent
Never	73	48.0%
Once	28	18.4%
2 to 5 times	33	21.7%
6 to 10 times	10	6.6%
10 to 20 times	6	3.9%
More than 20 times	2	1.3%
Total (N): all respondents stating "yes" to diagnosed with Afib	152	

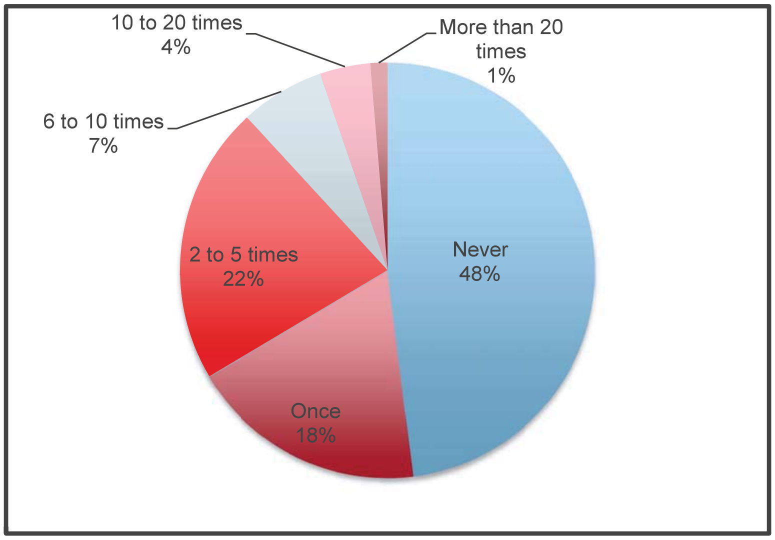

Q15A **NEW: Include only yes responders to Afib** Regarding the management of your atrial fibrillation-- what methods have been used to treat it? (Check all that apply)

Response	Count	Percent
Medication	117	77.0%
RFA - Radio Frequency Ablation - PVA or pathway	30	19.7%
Surgical - MAZE	20	13.2%
None	22	14.5%
Total (N): all respondents stating "yes" to diagnosed with Afib	152	

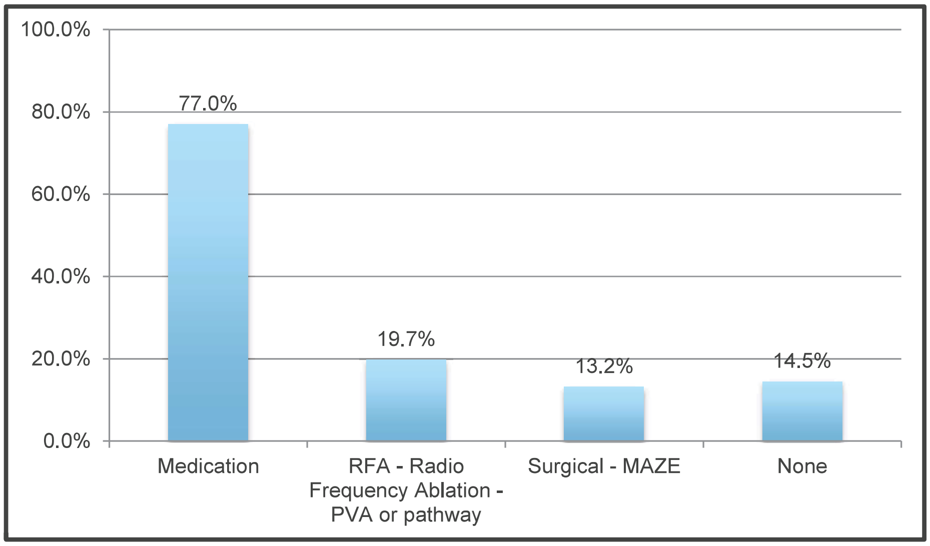

Q16 **Have you experienced any of the following? (Check all that apply)**

Response	Count	Percent
Tiredness	329	74.1%
Shortness of breath when you try to exert yourself	326	73.4%
Shortness of breath after meals	218	49.1%
Shortness of breath when lying flat	191	43.0%
Light-headedness	310	69.8%
Dizziness after exertion	239	53.8%
Fainting spells	108	24.3%
Palpitations	283	63.7%
Exercise intolerance	251	56.5%
Chest Pain (chest-- upper back-- jaw)	171	38.5%
Chest Pain upon exertion	163	36.7%
Other	27	6.1%
Total (N): all respondents	444	

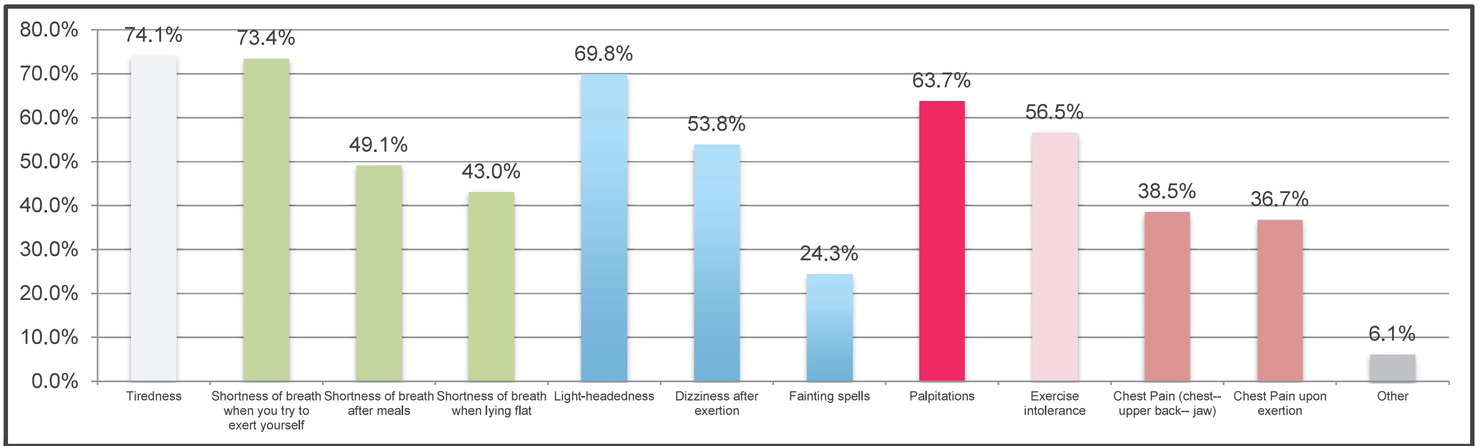

Q16A **NEW Number of Symptoms, Segmented by Obstruction**

	All patients		Obstructive		Non-Obstructive	
**Response**	**Count**	**Percent**	**Count**	**Percent**	**Count**	**Percent**
None	21	4.7%	4	1.6%	13	8.9%
One	31	7.0%	8	3.1%	21	14.4%
Two	34	7.7%	15	5.9%	16	11.0%
Three	37	8.3%	14	5.5%	16	11.0%
Four	39	8.8%	21	8.2%	10	6.8%
Five	40	9.0%	25	9.8%	10	6.8%
Six	46	10.4%	29	11.3%	13	8.9%
Seven	39	8.8%	26	10.2%	12	8.2%
Eight	35	7.9%	25	9.8%	7	4.8%
Nine or more	122	27.5%	89	34.8%	28	19.2%
Total	444		256		146	

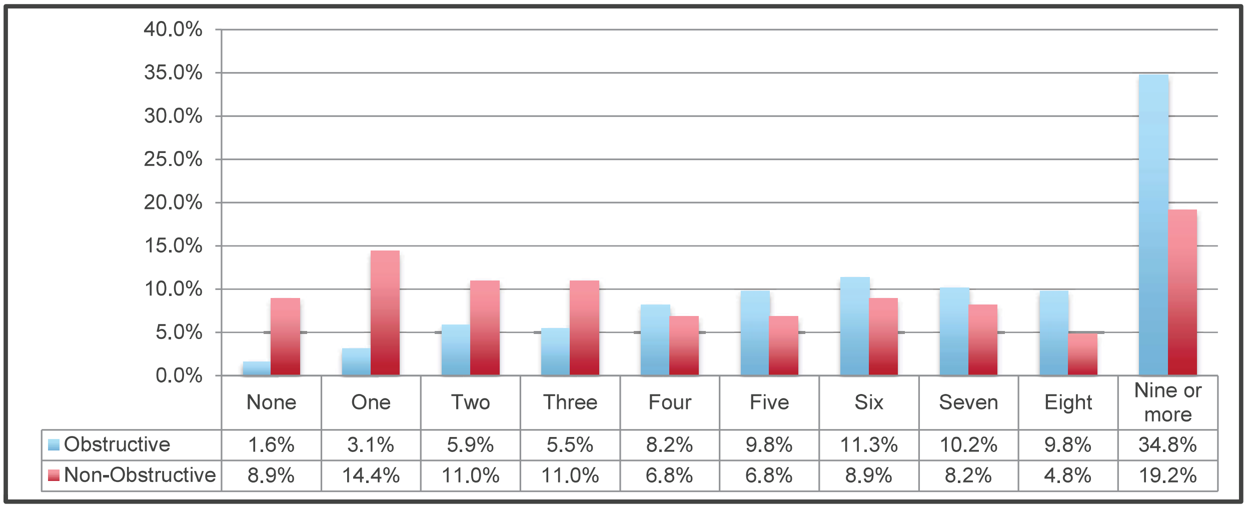

Q16B NEW Segment by obstruction Have you experienced any of the following? (Check all that apply)

	All patients		Obstructive		Non-obstructive	
Response	Count	Percent	Count	Percent	Count	Percent
Tiredness	329	74.1%	215	84.0%	85	58.2%
Shortness of breath when you try to exert yourself	326	73.4%	213	83.2%	82	56.2%
Shortness of breath after meals	218	49.1%	152	59.4%	53	36.3%
Shortness of breath when lying flat	191	43.0%	131	51.2%	47	32.2%
Light-headedness	310	69.8%	198	77.3%	87	59.6%
Dizziness after exertion	239	53.8%	163	63.7%	60	41.1%
Fainting spells	108	24.3%	75	29.3%	29	19.9%
Palpitations	283	63.7%	173	67.6%	89	61.0%
Exercise intolerance	251	56.5%	174	68.0%	64	43.8%
Chest Pain (chest-- upper back-- jaw)	171	38.5%	118	46.1%	41	28.1%
Chest Pain upon exertion	163	36.7%	115	44.9%	40	27.4%
Other	27	6.1%	14	5.5%	8	5.5%
Total (N)	444		256		146	

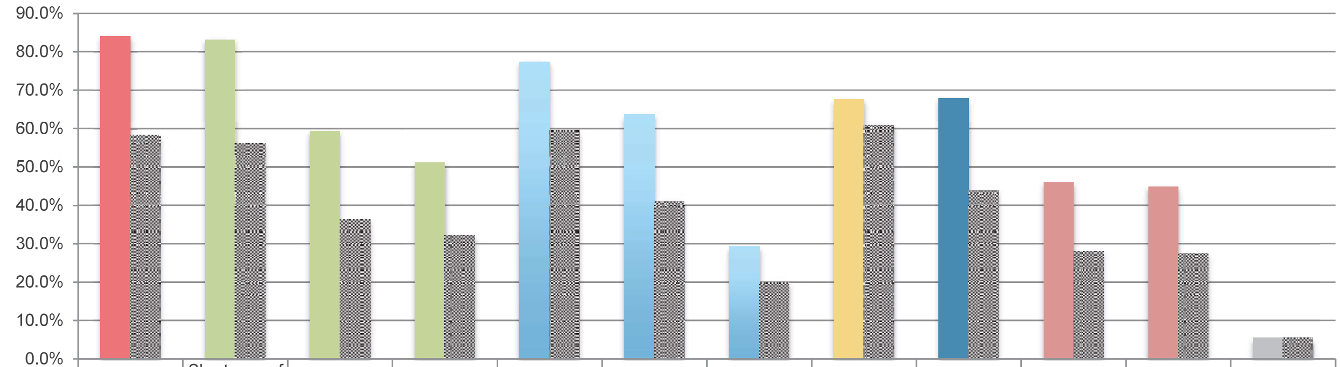

	Tiredness	Shortness of breath when you try to exert yourself	Shortness of breath after meals	Shortness of breath when lying flat	Light-headednes	Dizziness after exertion	Fainting spells	Palpitations	Exercise intolerance	Chest Pain (chest-- upper back-- jaw)	Chest Pain upon exertion	Other
Obstructive	84.0%	83.2%	59.4%	51.2%	77.3%	63.7%	29.3%	67.6%	68.0%	46.1%	44.9%	5.5%
Non-obstructive	58.2%	56.2%	36.3%	32.2%	59.6%	41.1%	19.9%	61.0%	43.8%	28.1%	27.4%	5.5%

Q17 **What was the time period between when you first started having symptoms until you saw your physician?**

Response	Count	Percent
I have not developed symptoms / I do not feel I have "symptoms"	65	15.1%
Less than 3 months	168	39.0%
3 months to 1 year	94	21.8%
1-3 years	36	8.4%
3-5 years	18	4.2%
More than 5 years	50	11.6%
Total	431	

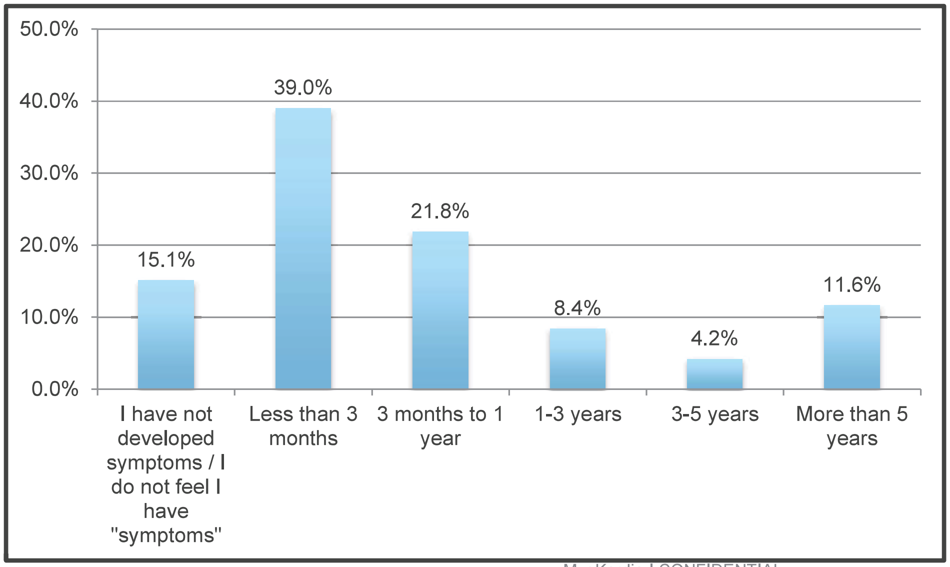

Q18 **How would you rate your symptoms?**

Response	Count	Percent	NYHA
No limitation of physical activity. Ordinary physical activity does not cause undue fatigue-- palpitation-- dyspnea (shortness of breath).	91	21.0%	I
Slight limitation of physical activity. Comfortable at rest. Ordinary physical activity results in fatigue-- palpitation-- dyspnea (shortness of breath).	183	42.2%	II
Marked limitation of physical activity. Comfortable at rest. Less than ordinary activity causes fatigue-- palpitation-- or dyspnea.	136	31.3%	III
Unable to carry on any physical activity without discomfort. Even at rest symptoms include dyspnea (shortness of breath)-- fatigue-- fainting (syncope) and chest pain. If any physical activity is undertaken-- discomfort increases.	24	5.5%	IV
Total	434		

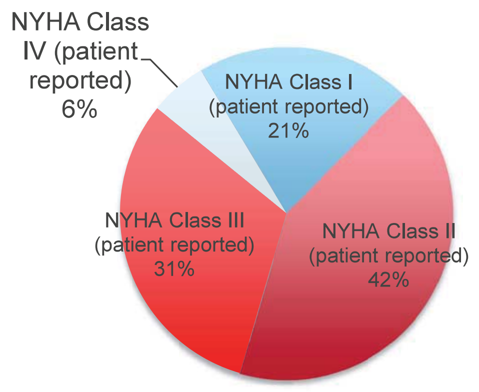

Q18B **NEW: Segment by obstruction and symptoms** **Proportion of patients with obstruction and symptoms**

Response	Count	Percent
Obstructive, NYHA Class I	28	7.1%
Obstructive, NYHA Class II-IV	223	56.5%
Non-Obstructive, NYHA Class I	53	13.4%
Non-Obstructive, NYHA Class II-IV	91	23.0%
Total	395	

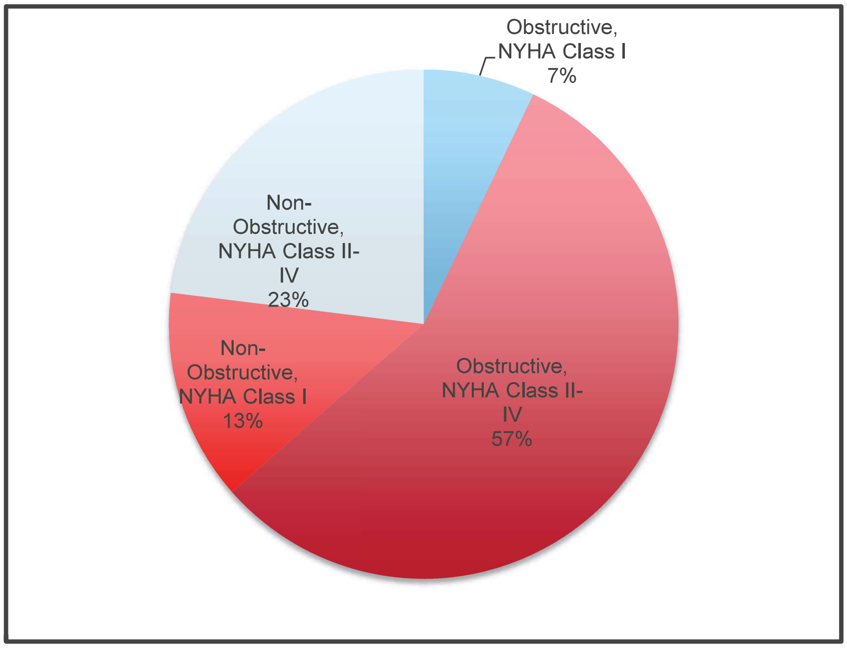

Q18C **NEW: Segment by patient origination** **How would you rate your symptoms?**

	All Patients		Symptoms		Unrelated Med Investig.		Screening	
**Response**	**Count**	**Percent**	**Count**	**Percent**	**Count**	**Percent**	**Count**	**Percent**
NYHA Class I (patient reported)	91	21.0%	28	12.1%	43	31.6%	20	30.3%
NYHA Class II (patient reported)	183	42.2%	96	41.4%	60	44.1%	27	40.9%
NYHA Class III (patient reported)	136	31.3%	92	39.7%	27	19.9%	17	25.8%
NYHA Class IV (patient reported)	24	5.5%	16	6.9%	6	4.4%	2	3.0%
Total	434		232		136		66	

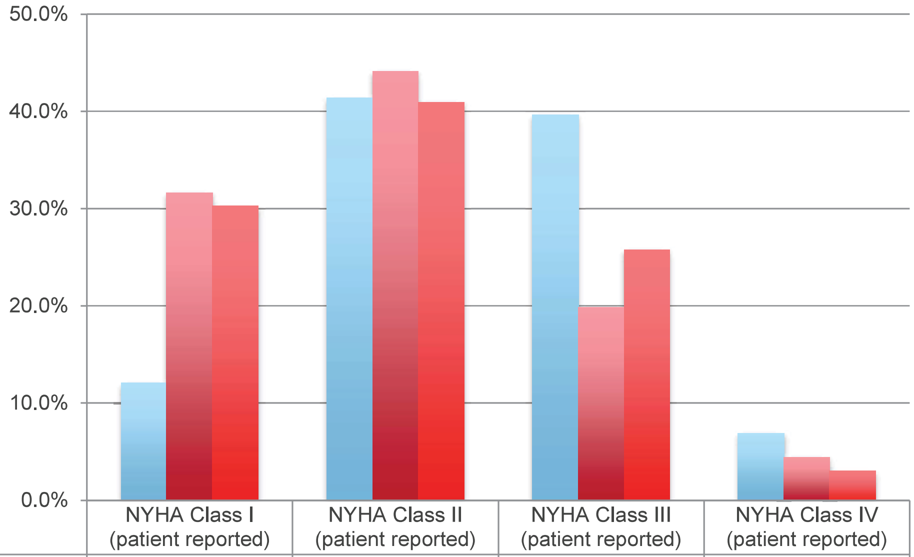

	NYHA Class I (patient reported)	NYHA Class II (patient reported)	NYHA Class III (patient reported)	NYHA Class IV (patient reported)
Symptoms	12.1%	41.4%	39.7%	6.9%
Unrelated Med Investig.	31.6%	44.1%	19.9%	4.4%
Screening	30.3%	40.9%	25.8%	3.0%

Q18E **NEW: Segment by obstruction and symptoms** Proportion of patients with obstruction and symptoms

All Patients

Response	Count	Percent
Obstructive, NYHA Class I	28	7.1%
Obstructive, NYHA Class II	115	29.1%
Obstructive, NYHA Class III	97	24.6%
Obstructive, NYHA Class IV	11	2.8%
Non-Obstructive, NYHA Class I	53	13.4%
Non-Obstructive, NYHA Class II	52	13.2%
Non-Obstructive, NYHA Class III	29	7.3%
Non-Obstructive, NYHA Class IV	10	2.5%
Total	395	

Obstructive

Response	Count	Percent
Obstructive, NYHA Class I	28	11.2%
Obstructive, NYHA Class II	115	45.8%
Obstructive, NYHA Class III	97	38.6%
Obstructive, NYHA Class IV	11	4.4%
Total	251	

Non-Obstructive

Response	Count	Percent
Non-Obstructive, NYHA Class I	53	36.8%
Non-Obstructive, NYHA Class II	52	36.1%
Non-Obstructive, NYHA Class III	29	20.1%
Non-Obstructive, NYHA Class IV	10	6.9%
Total	144	

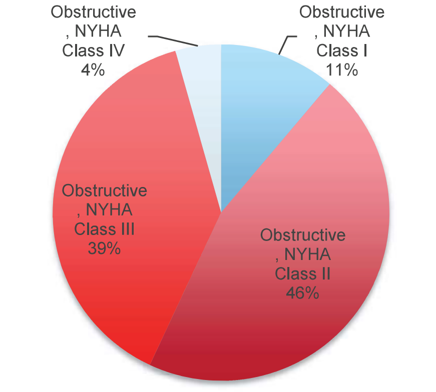

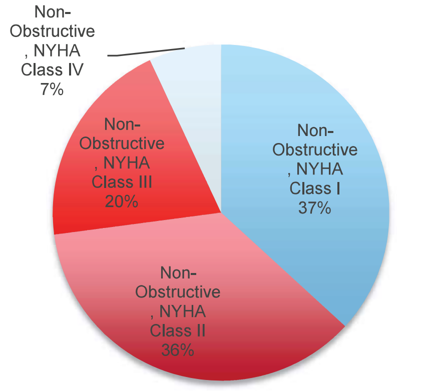

MyoKardia I CONFIDENTIAL
MyoKardia

Q19 **What was the time period between when you first experienced symptoms-- and mentioned them to a doctor-- until you were diagnosed with HCM?**

Response	Count	Percent
Less than 3 months	156	36.5%
3 months to 1 year	60	14.1%
1-3 years	48	11.2%
3-5 years	28	6.6%
More than 5 years	80	18.7%
I have never experienced symptoms	55	12.9%
Total	427	

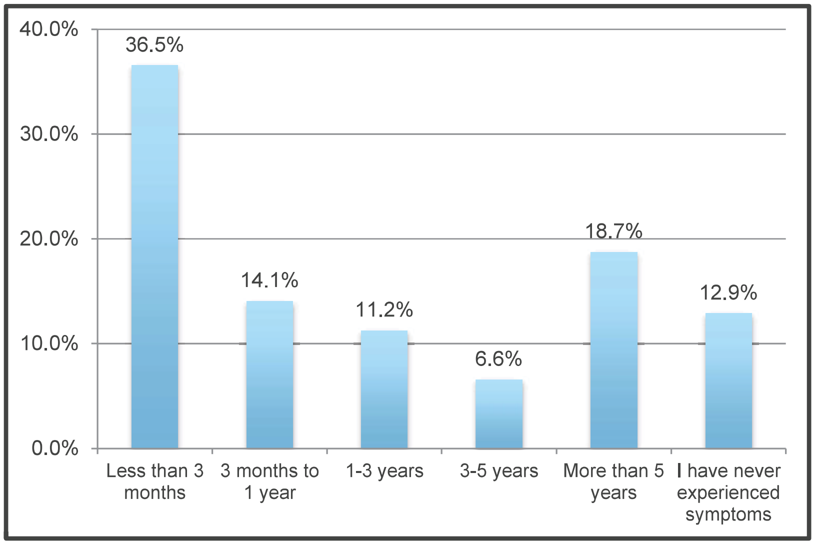

Q20 **How many living people in your family are known to have HCM or be genetic mutation carriers? (please remember to include those who have received heart transplantation)**

Response	Count	Percent
One	204	57.3%
two to five	117	32.9%
six to nine	23	6.5%
10+	12	3.4%
Total	356	

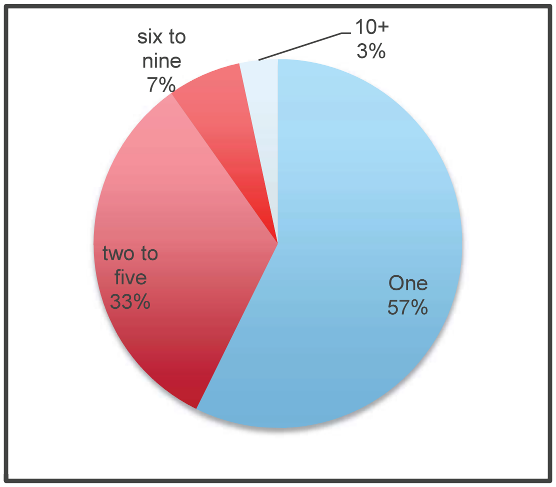

Q21 **How would you describe your diagnosis of HCM?**

Response	Count	Percent
Familial (runs in the family)	209	47.6%
Believed to be sporadic (I am the only member of my family known to have HCM)	131	29.8%
Associated with the diagnosis of another condition (for instance-- Noonan Syndrome-- Pompe Disease-- Danon Disease-- or other)	4	0.9%
I don't know	95	21.6%
Total	439	

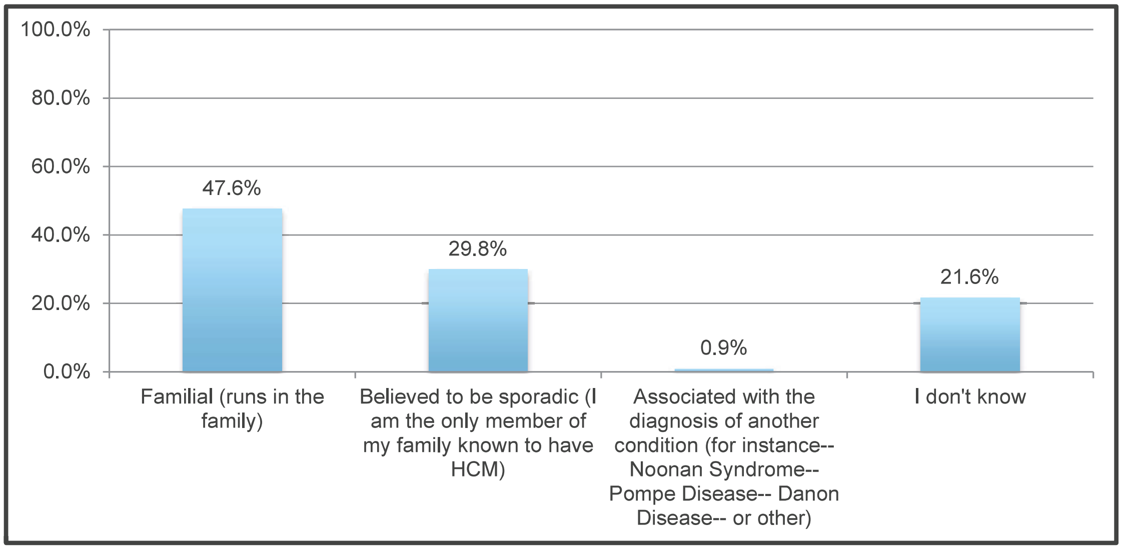

Q22 **Have there been any HCM related deaths in your family? Please include sudden death, heart failure or strokes in those with HCM.**

Response	Count	Percent
Yes	145	33.0%
No	149	33.9%
Unsure	146	33.2%
Total	440	

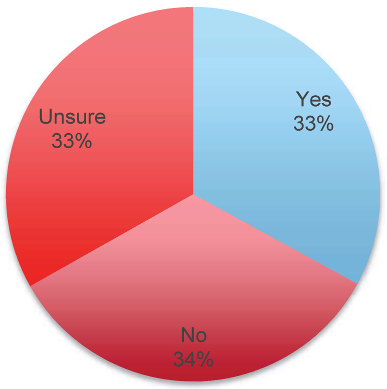

Q23 **Are you seeing a cardiologist who specializes in HCM and is located in a university center or at a hospital clinic for cardiomyopathy? This includes an HCMA recognized Center of Excellence.**

Response	Count	Percent
Yes-- I see a Cardiologist at either an HCMA recognized Center of Excellence or a University Center with a Cardiomyopathy program	301	68.4%
No	139	31.6%
Total	440	

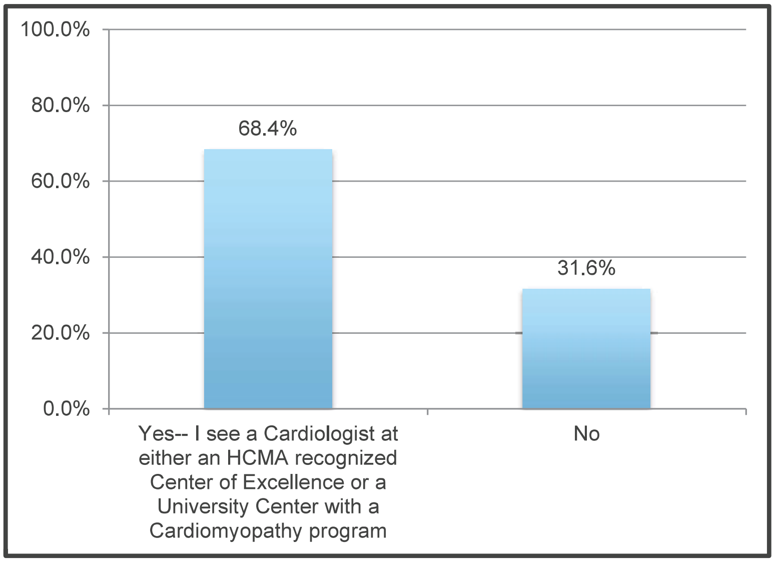

Q24A **NEW: Include only yes responders to seeing HCM spec** If yes-- how often do you see your HCM specialist to monitor your HCM?

Response	Count	Percent
Every 3 months	25	8.4%
Every 6 months	94	31.5%
Annually	129	43.3%
Every 2 years	28	9.4%
Every 3 or more years	22	7.4%
Total	298	

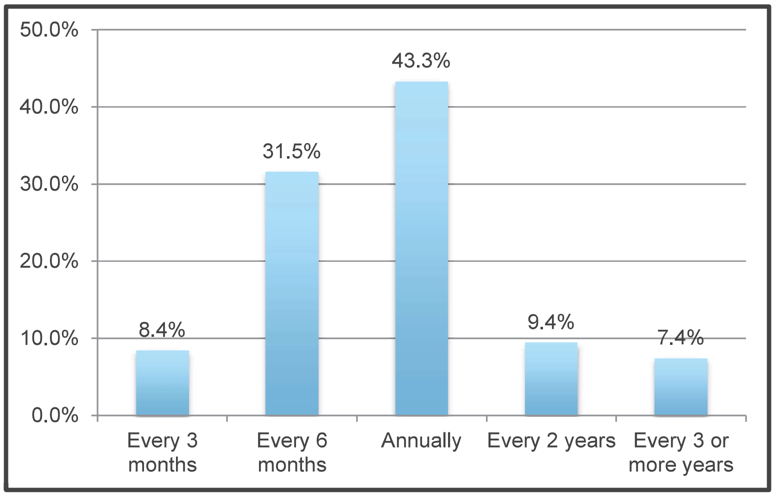

Q25A **NEW: Include only yes responders to seeing HCM spec If yes-- how far do you travel to see your HCM specialist?**

Response	Count	Percent
<10 miles	49	16.3%
10 to <30 miles	48	16.0%
30 to <50 miles	51	17.0%
50 to <100 miles	39	13.0%
More than 100 miles	113	37.7%
Total	300	

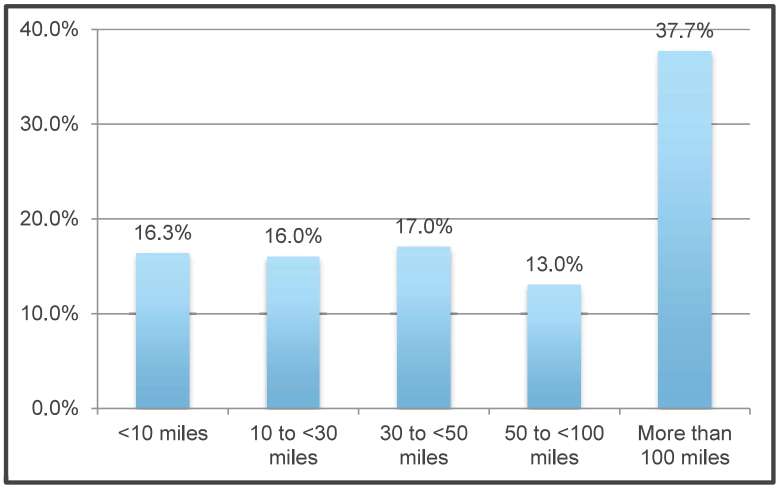

Q26A **NEW: Include only yes responders to seeing HCM spec** **If yes-- how many HCM specialists have you seen in the past 5 years?**

Response	Count	Percent
One	151	50.3%
Two	100	33.3%
Three	36	12.0%
Four	5	1.7%
Five or more	8	2.7%
Total	300	

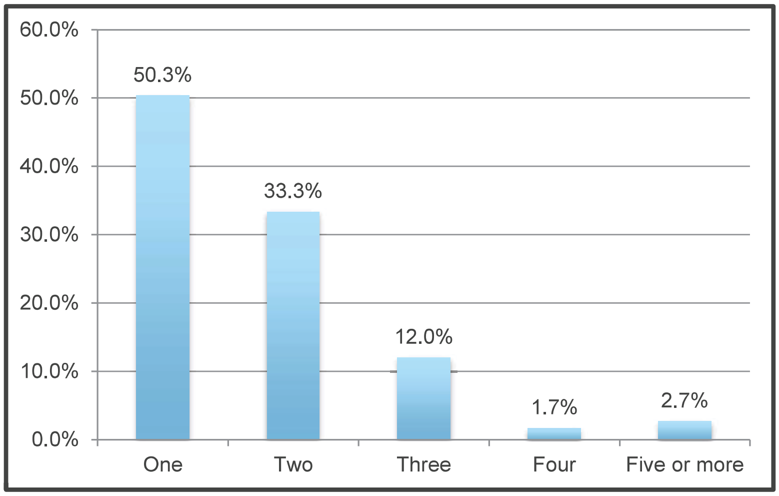

Q27 **Are you seeing a general cardiologist-- who does not specialize in HCM?**

Response	Count	Percent
Yes	269	60.7%
No	174	39.3%
Total	443	

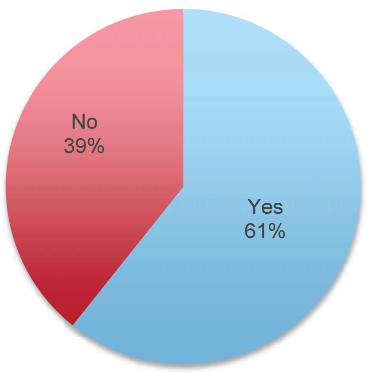

Q28A **NEW: Include only yes responders to seeing gen card** If yes-- how often do you check in with your general cardiologist to monitor your HCM?

Response	Count	Percent
Every 3 months	48	18.2%
Every 6 months	117	44.3%
Annually	87	33.0%
Every 2 years	9	3.4%
Every 3 or more years	3	1.1%
Total	264	

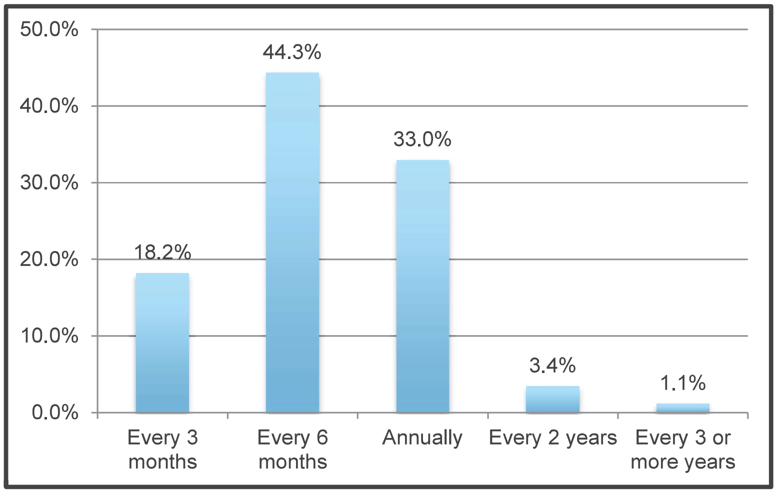

Q29A **NEW: Include only yes responders to seeing gen card** **If yes-- how far do you travel to see your general cardiologist?**

Response	Count	Percent
<10 miles	138	51.9%
10 to <30 miles	79	29.7%
30 to <50 miles	25	9.4%
50 to <100 miles	18	6.8%
More than 100 miles	6	2.3%
Total	266	

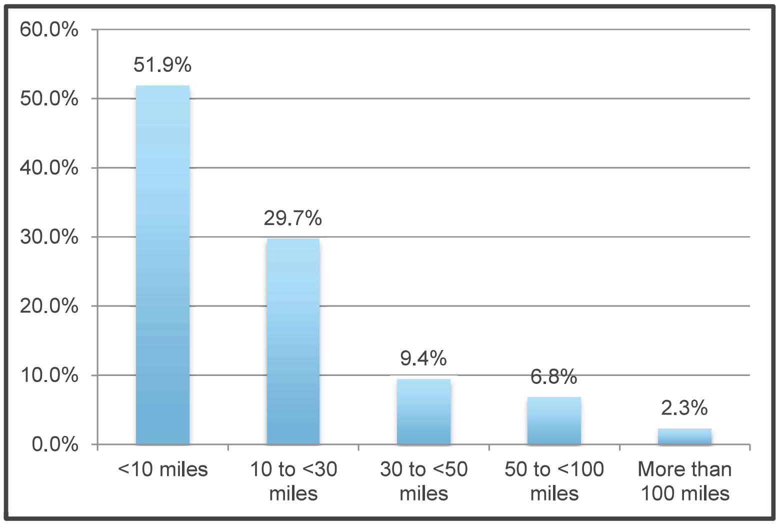

Q30A **NEW: Include only yes responders to seeing gen card** **If yes-- how many general cardiologists have you seen in the past 5 years?**

Response	Count	Percent
One	101	37.8%
Two	104	39.0%
Three	42	15.7%
Four	8	3.0%
Five or more	12	4.5%
Total	267	

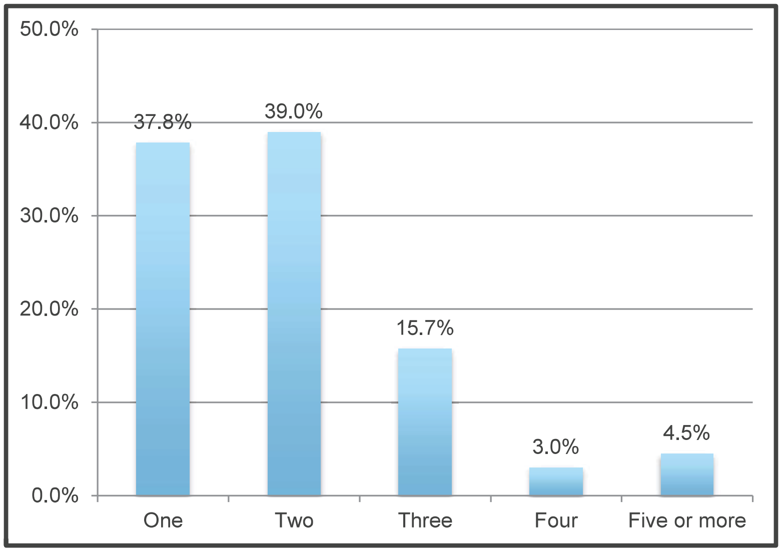

MyoKardia I CONFIDENTIAL

Q31 Which of the following medications do you currently take? (Check all that apply)

If you have switched from one medication to another within the same class please check the box that best applies - example: it is common to switch from one beta blocker to a different one. In this case you would check Beta Blocker both in the area of taking and previously taken (the next question).

Response	Count	Percent
Beta blockers (propranolol-- nadolol-- atenolol-- acebutolol-- metroprolol-- Lopressor-- Inderal)	315	70.9%
Calcium channel blockers (diazetam-- nifedipine-- verapamil)	105	23.6%
Diuretics (HCZT-- Lasix-- Torsemide-- Spironolactone)	104	23.4%
Anticoagulant medications (blood thinners)	101	22.7%
Other	48	10.8%
ACE inhibitors ("-prils" or ARBs ("-sartans")	38	8.6%
Antiarrhythmic medications (like amiodarone)	38	8.6%
Disopyramide (Norpace)	34	7.7%
Aldosterone antagonists (aldactone or eplerenone)	8	1.8%
Total (N): all respondents	444	

Q31 **Which of the following medications do you currently take? (Check all that apply)**

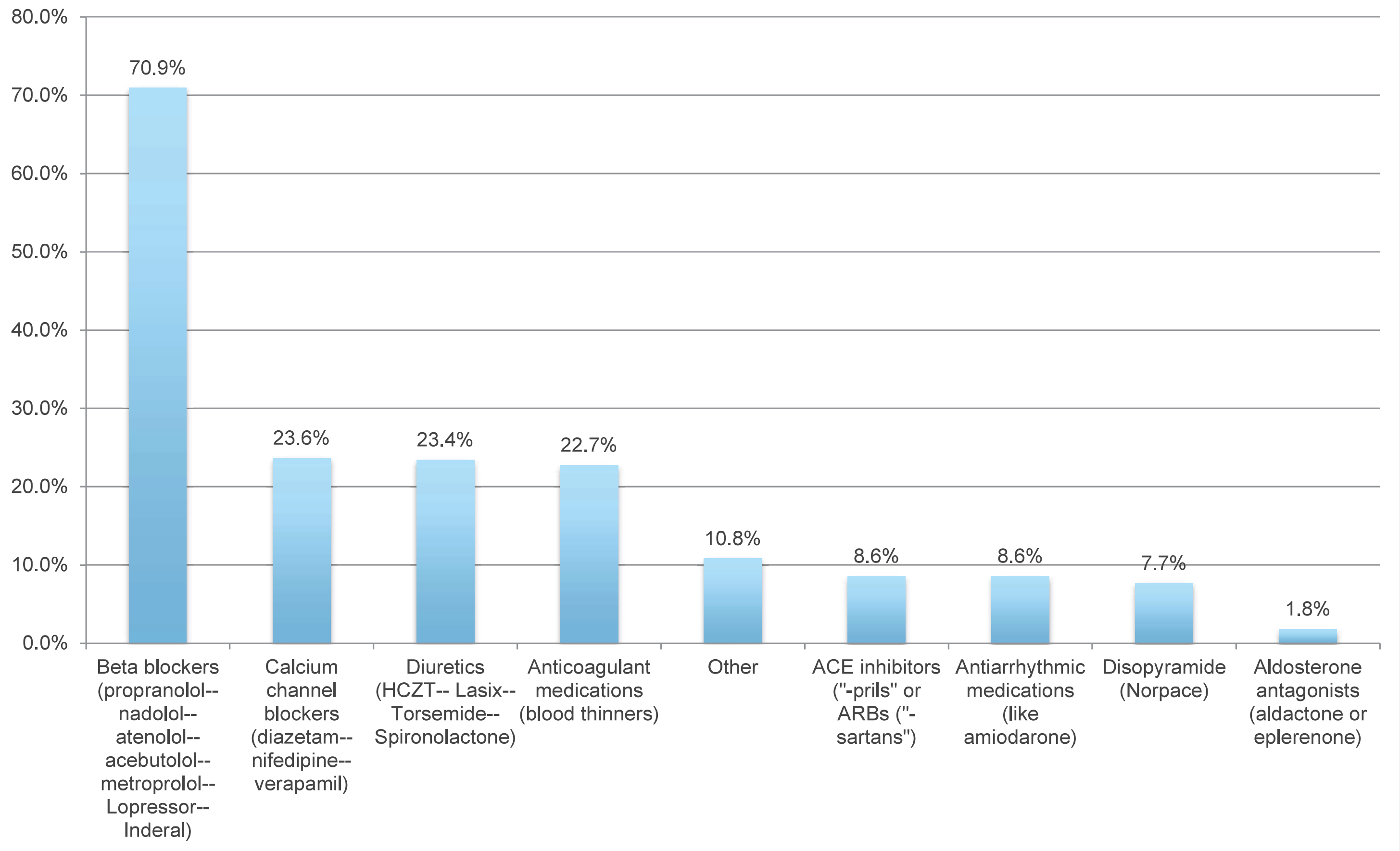

Q31A **NEW: Segment by NYHA Class** Which of the following medications do you currently take? (Check all that apply)

If you have switched from one medication to another within the same class please check the box that best applies - example: it is common to switch from one beta blocker to a different one. In this case you would check Beta Blocker both in the area of taking and previously taken (the next question).

	All Patients		NYHA I		NYHA II		NYHA III		NYHA IV		NYHA II-IV	
**Response**	**Count**	**Percent**	**Count**	**Percent**	**Count**	**Percent**	**Count**	**Percent**	**Count**	**Percent**	**Count**	**Percent**
Beta blockers (propranolol-- nadolol-- atenolol-- acebutolol-- metroprolol-- Lopressor-- Inderal)	315	70.9%	56	61.5%	133	72.7%	103	75.7%	16	66.7%	252	73.5%
Calcium channel blockers (diazetam-- nifedipine-- verapamil)	105	23.6%	14	15.4%	49	26.8%	36	26.5%	5	20.8%	90	26.2%
Diuretics (HCZT-- Lasix-- Torsemide-- Spironolactone)	104	23.4%	13	14.3%	32	17.5%	47	34.6%	11	45.8%	90	26.2%
Anticoagulant medications (blood thinners)	101	22.7%	16	17.6%	40	21.9%	35	25.7%	8	33.3%	83	24.2%
Other	48	10.8%	9	9.9%	15	8.2%	17	12.5%	5	20.8%	37	10.8%
ACE inhibitors ("-prils" or ARBs ("-sartans")	38	8.6%	4	4.4%	22	12.0%	10	7.4%	2	8.3%	34	9.9%
Antiarrhythmic medications (like amiodarone)	38	8.6%	7	7.7%	11	6.0%	16	11.8%	3	12.5%	30	8.7%
Disopyramide (Norpace)	34	7.7%	3	3.3%	15	8.2%	13	9.6%	3	12.5%	31	9.0%
Aldosterone antagonists (aldactone or eplerenone)	8	1.8%	1	1.1%	2	1.1%	4	2.9%	1	4.2%	7	2.0%
Total (N): all respondents by NYHA class	444		91		183		136		24		343	

Q31A **NEW: Segment by NYHA Class** Which of the following medications do you currently take? (Check all that apply)

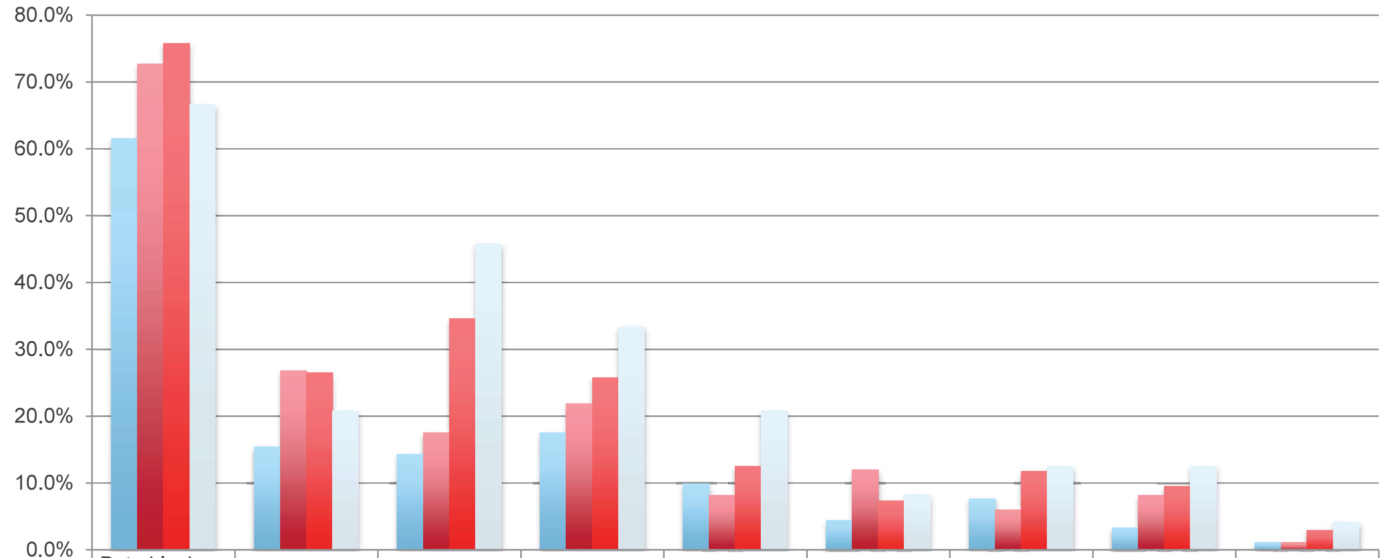

	Beta blockers (propranolol--nadolol--atenolol--acebutolol--metroprolol--Lopressor--Inderal)	Calcium channel blockers (diazetam--nifedipine--verapamil)	Diuretics (HCZT--Lasix--Torsemide--Spironolactone)	Anticoagulant medications (blood thinners)	Other	ACE inhibitors ("-prils" or ARBs ("-sartans")	Antiarrhythmic medications (like amiodarone)	Disopyramide (Norpace)	Aldosterone antagonists (aldactone or eplerenone)
NYHA I	61.5%	15.4%	14.3%	17.6%	9.9%	4.4%	7.7%	3.3%	1.1%
NYHA II	72.7%	26.8%	17.5%	21.9%	8.2%	12.0%	6.0%	8.2%	1.1%
NYHA III	75.7%	26.5%	34.6%	25.7%	12.5%	7.4%	11.8%	9.6%	2.9%
NYHA IV	66.7%	20.8%	45.8%	33.3%	20.8%	8.3%	12.5%	12.5%	4.2%

MyoKardia I CONFIDENTIAL
MyoKardia

Q32 **Which of the following medications have you taken in the past-- but are no longer taking? (Check all that apply).**

If you have switched from one medication to another within the same class please check the box that best applies - example: it is common to switch from one beta blocker to a different one in this case you would check Beta Blocker both in the area of taking and previously taken.

Response	Count	Percent
Beta blockers (propranolol-- nadolol-- atenolol-- acebutolol-- metroprolol-- Lopressor-- Inderal)	170	38.3%
Calcium channel blockers (diazetam-- nifedipine-- verapamil)	113	25.5%
Disopyramide (Norpace)	53	11.9%
Anticoagulant medications (blood thinners)	49	11.0%
Antiarrhythmic medications (like amiodarone)	49	11.0%
Diuretics (HCZT-- Lasix-- Torsemide-- Spironolactone)	48	10.8%
ACE inhibitors ("-prils" or ARBs ("-sartans")	35	7.9%
Other	16	3.6%
Aldosterone antagonists (aldactone or eplerenone)	7	1.6%
Total (N): all respondents	444	

Q32 **Which of the following medications have you taken in the past-- but are no longer taking? (Check all that apply).**

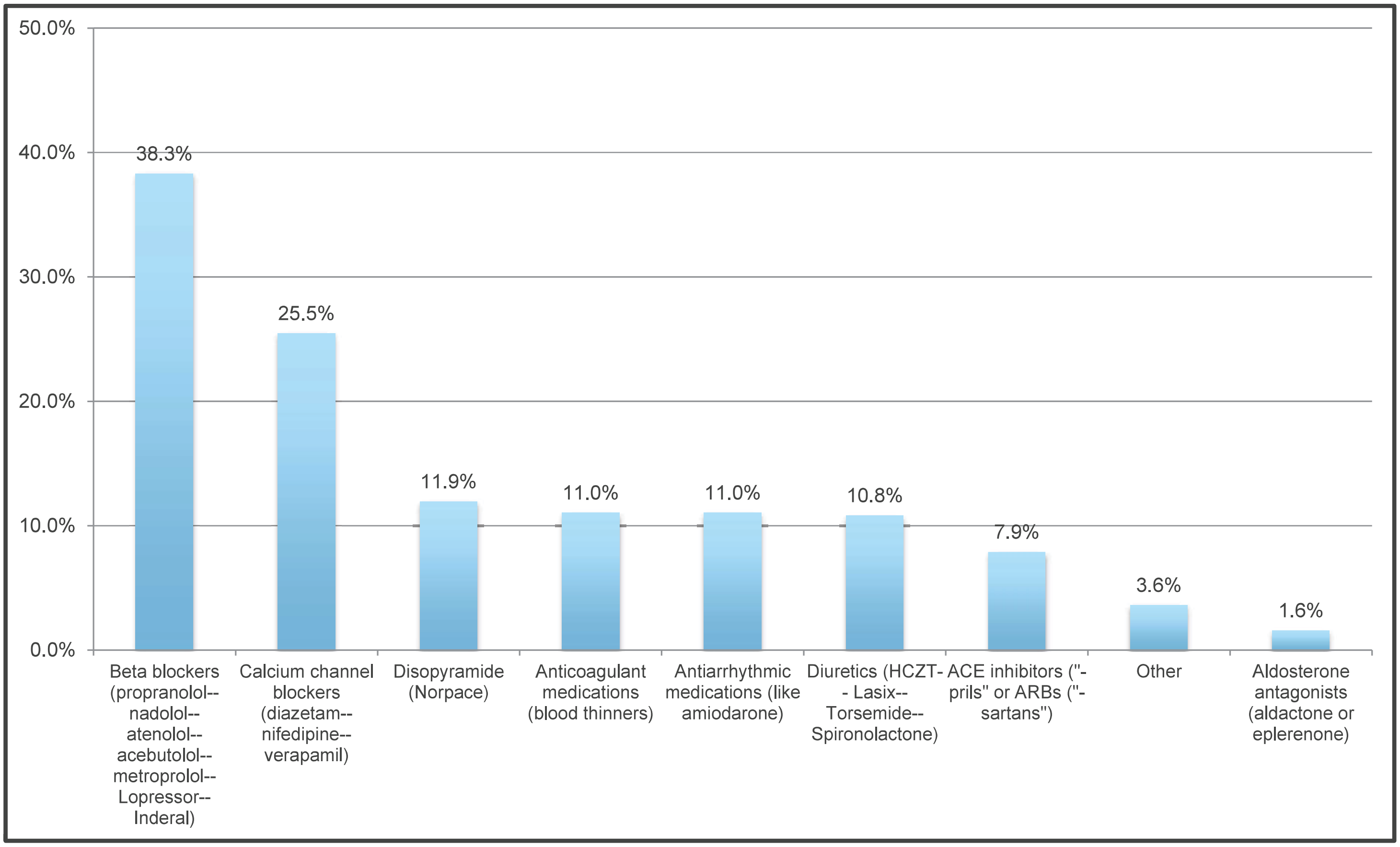

MyoKardia I CONFIDENTIAL

Q33 **How satisfied are you with how well your current medications for HCM work?**

Response	Count	Percent
Very dissatisfied	33	8.0%
Dissatisfied	27	6.5%
Neutral	101	24.4%
Satisfied	161	38.9%
Very satisfied	92	22.2%
Total	414	

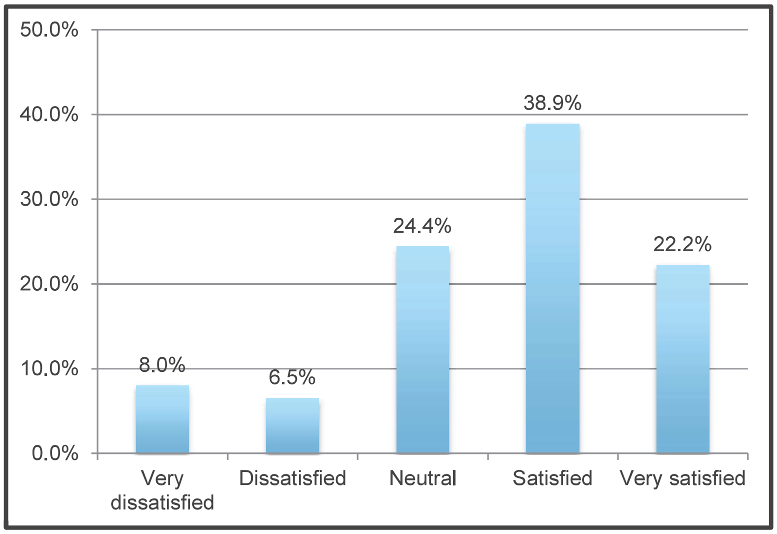

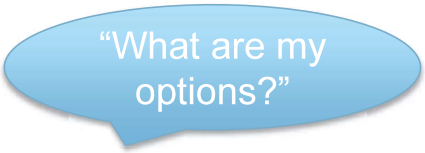

Q33a **New: Segment by NYHA class** How satisfied are you with how well your current medications for HCM work?

	All Patients		Class I		Class II-IV		Class III-IV	
**Response**	**Count**	**Percent**	**Count**	**Percent**	**Count**	**Percent**	**Count**	**Percent**
Very dissatisfied	33	8.0%	12	16.2%	20	6.0%	10	6.4%
Dissatisfied	27	6.5%	2	2.7%	25	7.5%	15	9.6%
Neutral	101	24.4%	16	21.6%	82	24.7%	44	28.2%
Satisfied	161	38.9%	21	28.4%	137	41.3%	63	40.4%
Very satisfied	92	22.2%	23	31.1%	68	20.5%	24	15.4%
Total	414		74		332		156	

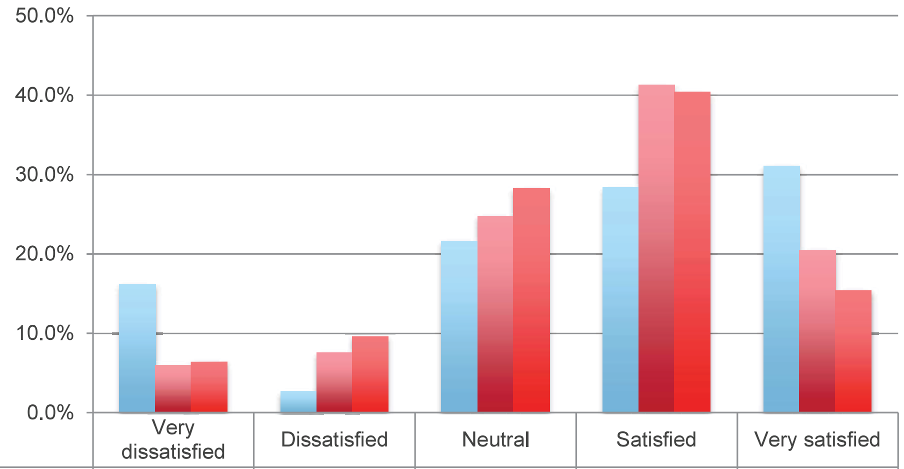

	Very dissatisfied	Dissatisfied	Neutral	Satisfied	Very satisfied
Class I	16.2%	2.7%	21.6%	28.4%	31.1%
Class II-IV	6.0%	7.5%	24.7%	41.3%	20.5%
Class III-IV	6.4%	9.6%	28.2%	40.4%	15.4%

MyoKardia

Q34 **When did you start taking daily medications for HCM?**

Response	Count	Percent
Not taking daily medications for HCM	37	8.5%
Within the last year	28	6.4%
1 to 2 years ago	37	8.5%
3 or more years ago	335	76.7%
Total	437	

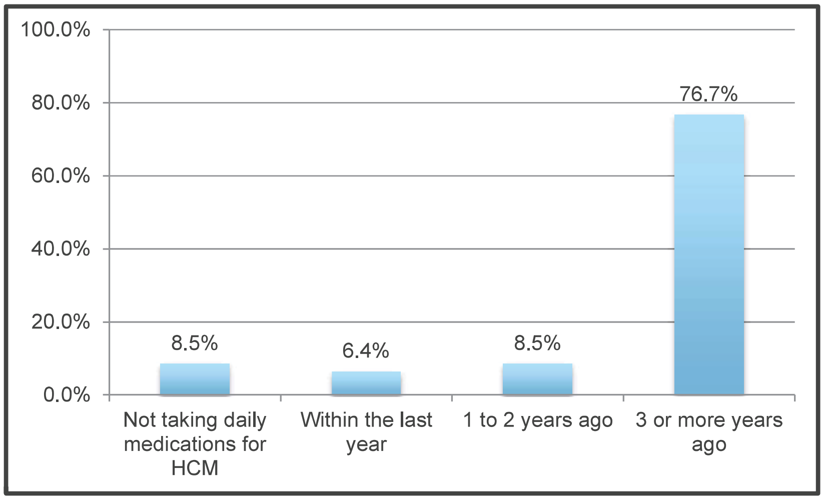

Q35 **How burdensome is your existing medication regimen for HCM?**

Response	Count	Percent
Not at all	220	52.4%
Slightly	104	24.8%
Somewhat	72	17.1%
Significantly	19	4.5%
Unwilling to take my medications	5	1.2%
Total	420	

“What else can I do?”

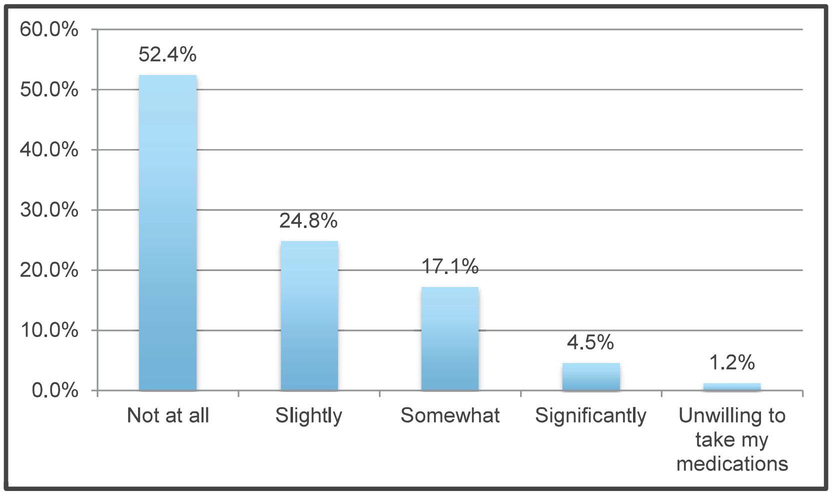

Q36 How often do you miss or skip taking your medication for HCM?

Response	Count	Percent
Never	231	56.8%
Once a month	130	31.9%
Once a week	27	6.6%
2-3 times a week	15	3.7%
4-5 times a week	0	0.0%
Most days	4	1.0%
Total	407	

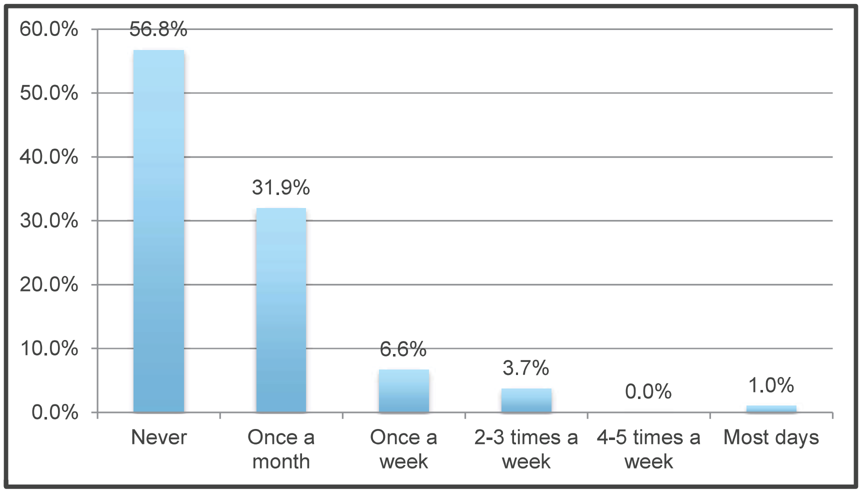

Q37 **Do you have an ICD (Implantable Cardioverter-Defibrillator)?**

Response	Count	Percent
Yes	209	47.2%
No	234	52.8%
Total	443	

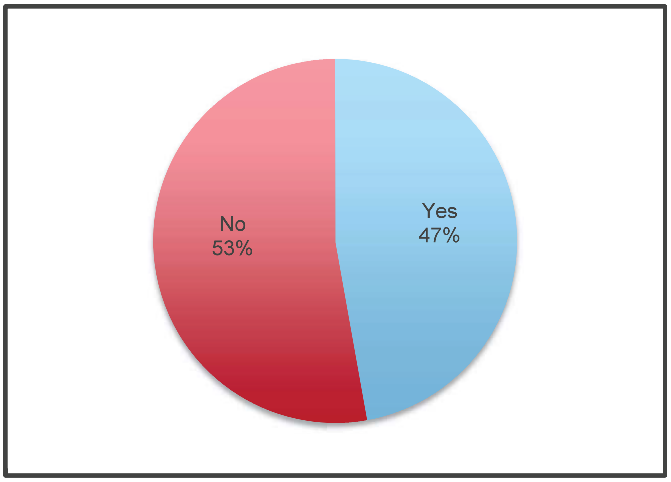

Q38A **NEW: Include only yes responders to "do you have an ICD?"** If yes-- how long have you had an ICD?

Response	Count	Percent
Less than two years	30	14.4%
2 to 5 years	67	32.1%
5 to 10 years	72	34.4%
More than 10 years	39	18.7%
Total (N): all respondents with an ICD	209	

Q39A **NEW: Include only yes responders to "do you have an ICD?"** If yes-- how many different ICDs have you had?

Response	Count	Percent
One	116	55.5%
Two	54	25.8%
Three or more	37	17.7%
Total (N): all respondents with an ICD	209	

Q40A **NEW: Include only yes responders to "do you have an ICD?"** If yes-- how often have you received an appropriate discharge (shock) from your ICD?

Response	Count	Percent
Never	156	74.6%
I have had less then 2 shocks	34	16.3%
I have between 3 and 6 shocks	8	3.8%
I have had over 7 shocks	9	4.3%
Total (N): all respondents with an ICD	209	

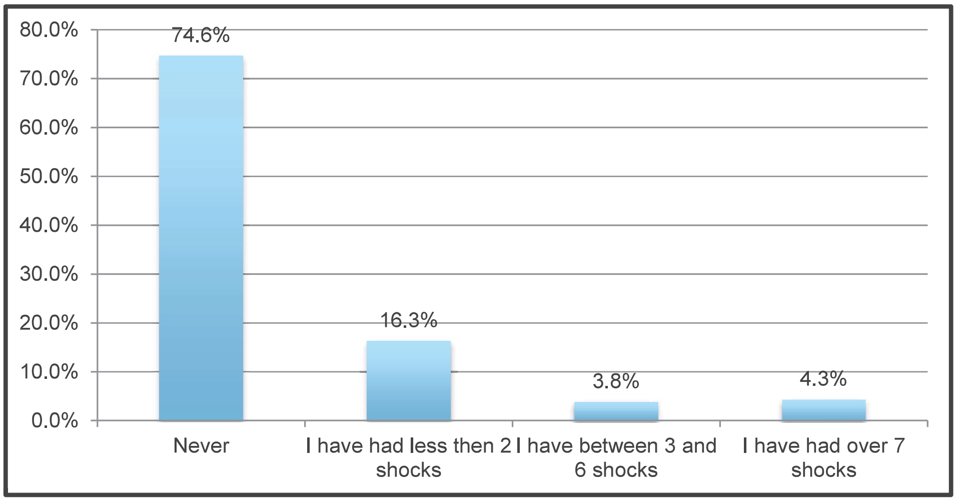

Q41A NEW: Include only yes responders to "do you have an ICD?" If yes-- how often have your received an inappropriate or non-therapeutic discharge (shock) from your ICD (for instance-- due to lead problems-- incorrect settings-- or other reason)?

Response	Count	Percent
Never	155	74.2%
Rarely	22	10.5%
Occasionally	5	2.4%
Often	1	0.5%
Total (N): all respondents with an ICD	209	

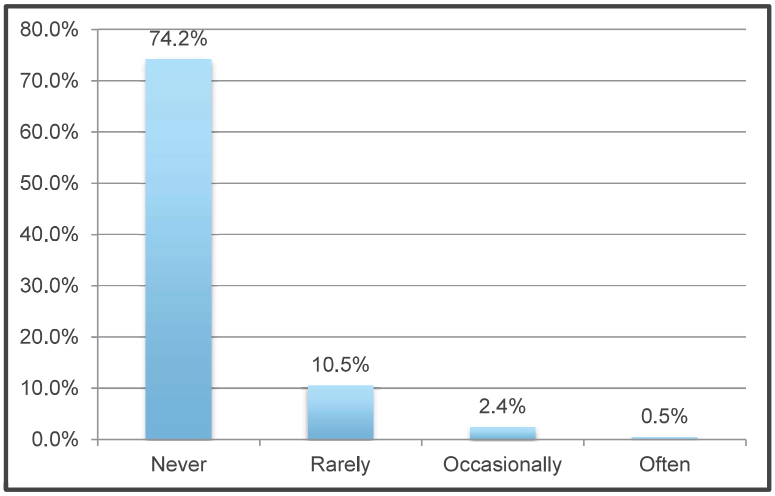

Q42 **Have you ever been told that you are a candidate for a septal reduction procedure-- to reduce obstruction (myectomy or alcohol ablation)?**

Response	Count	Percent
Yes	226	50.9%
No	218	49.1%
Total	444	

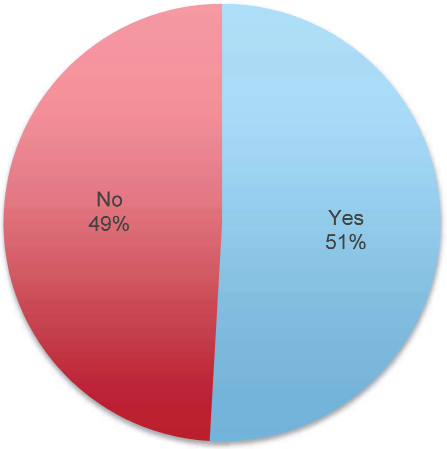

Q43A **NEW: Include only yes responders to "have you been told you are a candidate"** If yes-- have you had a procedure to reduce obstruction?

Response	Count	Percent
Yes	167	73.9%
No	59	26.1%
Total (N): all responders to "yes I have been told I am a candidate for septal reduction procedure"	226	

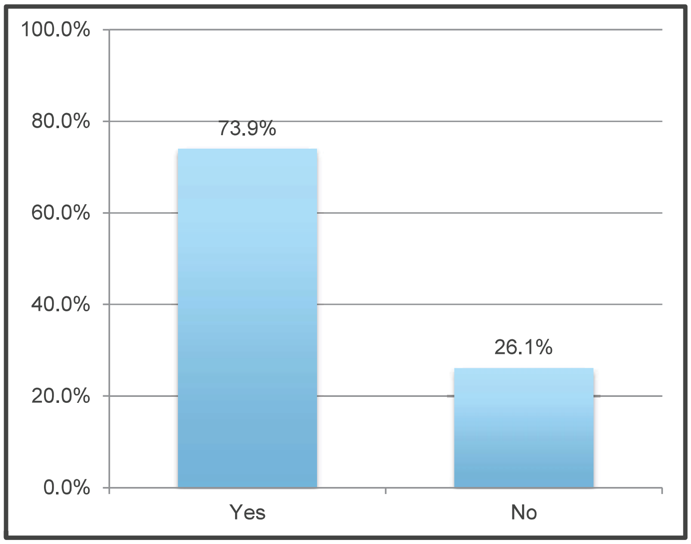

Q44 Have you had a surgical myectomy to reduce obstruction?

Response	Count	Percent
Yes	159	36.9%
No	272	63.1%
Total	431	

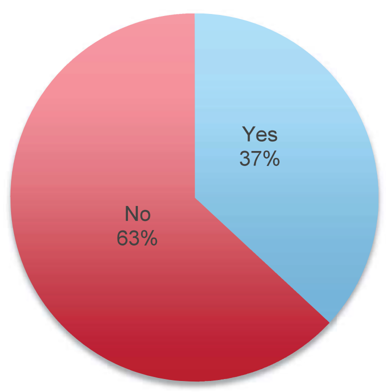

Q44A **NEW: For those who have had a myectomy, what is NYHA symptom class?**

Response	Count	Percent
NYHA Class I	18	11.5%
NYHA Class II	70	44.6%
NYHA Class III	60	38.2%
NYHA Class IV	9	5.7%
Total	157	

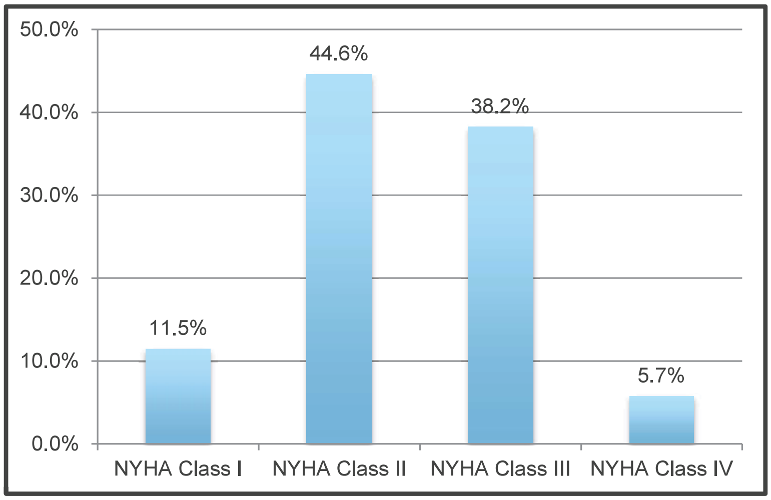

Q45 When was the myectomy performed?

Response	Count	Percent
Within that last year	28	17.7%
2 to 5 years ago	59	37.3%
5 to 10 years ago	48	30.4%
10 to 20 years ago	22	13.9%
More than 20 years ago	1	0.6%
Total	158	

Q46 **Have you had alcohol septal ablation to reduce obstruction?**

Response	Count	Percent
Yes	31	7.1%
No	406	92.9%
Total	437	

Q47A **NEW - Include only responders who said yes to ASA** **If yes-- how many times have you had alcohol septal ablation?**

Response	Count	Percent
Once	27	90.0%
Twice	2	6.7%
Three times	0	0.0%
Four or more times	1	3.3%
Total	30	

Q48A **NEW - Include only responders who said yes to ASA** When was the most recent alcohol ablation performed?

Response	Count	Percent
Within that last year	5	16.7%
2 to 5 years ago	8	26.7%
5 to 10 years ago	9	30.0%
10 to 20 years ago	8	26.7%
More than 20 years ago	0	0.0%
Total	30	

Q49 **Have you discussed options to reduce your outflow tract gradient (obstruction) -- a myectomy or an alcohol ablation procedure with your doctor-- but declined to have the procedure performed? Pick the best option.**

Response	Count	Percent
No - did not discuss	214	62.8%
Yes-- I have discussed one or both however at this time I am attempting to manage my symptoms with medication	76	22.3%
Yes - discussed and declined alcohol ablation	35	10.3%
Yes - discussed and declined myectomy	11	3.2%
Yes - discussed and declined both myectomy and alcohol ablation	5	1.5%
Total	341	

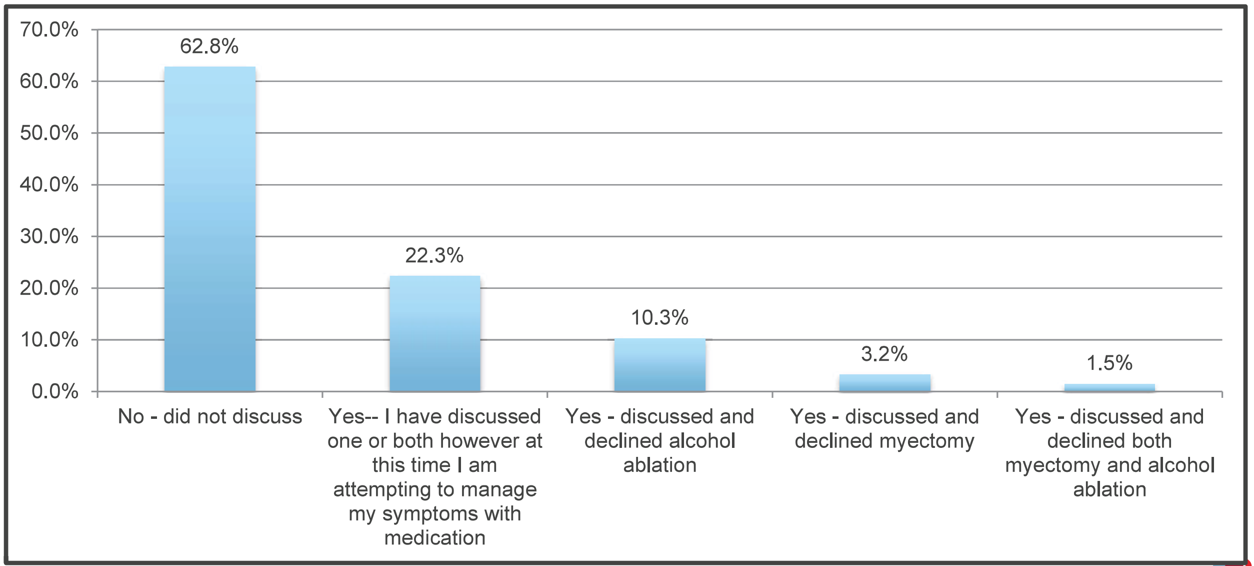

Q49A **NEW: Include only respondents who have ever been told they are a candidate for septal reduction procedure (myectomy or ASA)** **Have you discussed options to reduce your outflow tract gradient (obstruction) -- a myectomy or an alcohol ablation procedure with your doctor-- but declined to have the procedure performed? Pick the best option.**

Response	Count	Percent
No - did not discuss	53	38.1%
Yes-- I have discussed one or both however at this time I am attempting to manage my symptoms with medication	39	28.1%
Yes - discussed and declined alcohol ablation	33	23.7%
Yes - discussed and declined myectomy	11	7.9%
Yes - discussed and declined both myectomy and alcohol ablation	3	2.2%
Total	139	

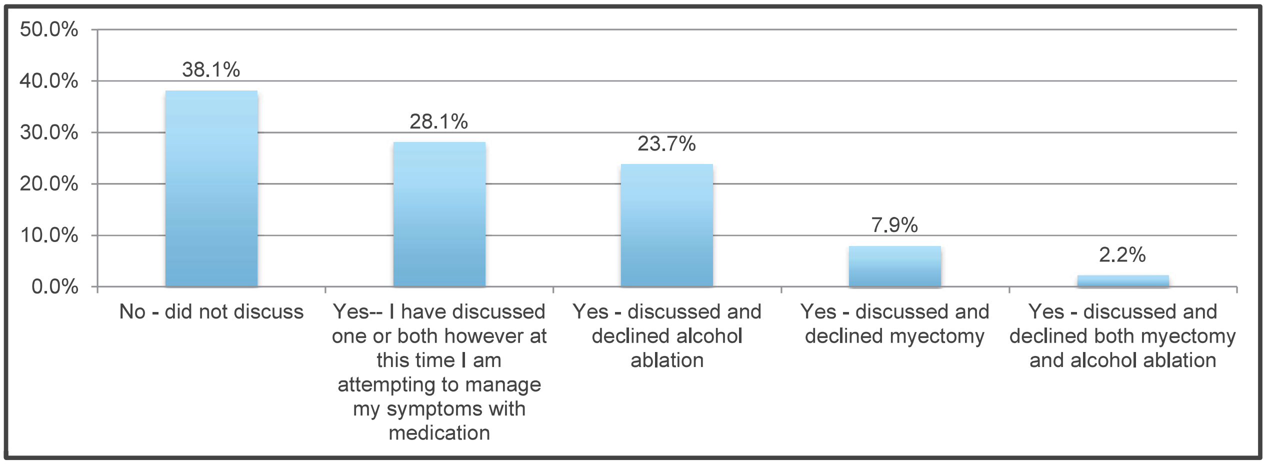

MyoKardia I CONFIDENTIAL
MyoKardia

Q50A **NEW: Include only responders who discussed and declined** **If you declined a procedure-- for what reasons? (Check all that apply)**

Response	Count	Percent
Concerned about effectiveness	37	29.1%
Other	32	25.2%
Concerned about safety	29	22.8%
Concerned about side effects	27	21.3%
Fear about invasive procedure	22	17.3%
Cost is too great	9	7.1%
Insurance barriers	6	4.7%
Total (N): candidates who declined procedure	127	

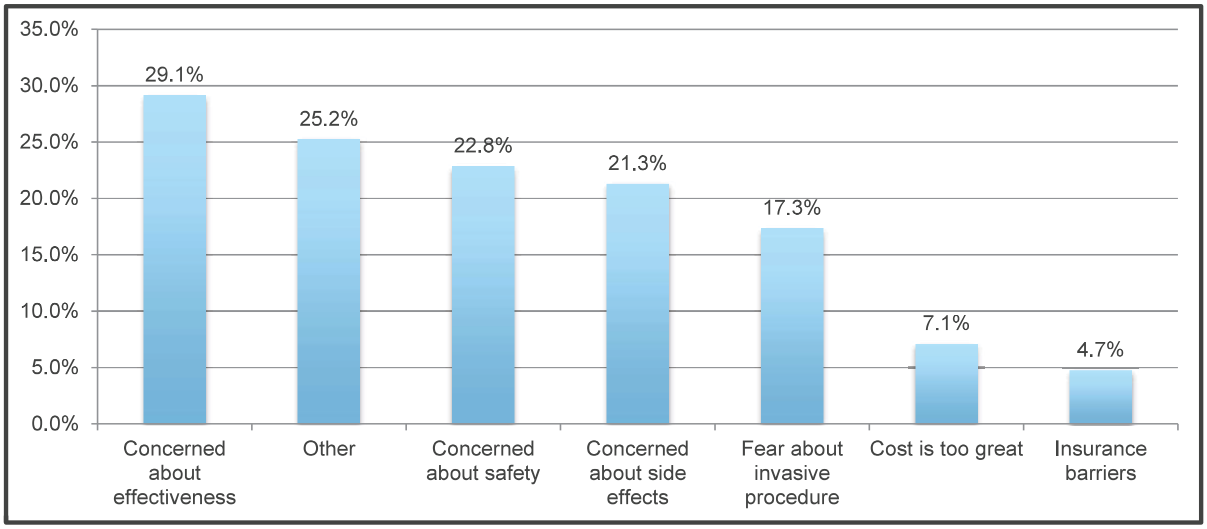

Q51 Have you had a heart transplant?

Response	Count	Percent
Yes	5	1.1%
No	434	98.9%
Total	439	

Q52 **How many times over your lifetime have you visited the emergency department or been admitted to the hospital due to cardiac symptoms?**

Response	Count	Percent
None	150	33.9%
Once	86	19.4%
Twice	63	14.2%
Three times	48	10.8%
Four times	21	4.7%
Five times	24	5.4%
Six or more times	51	11.5%
Total	443	

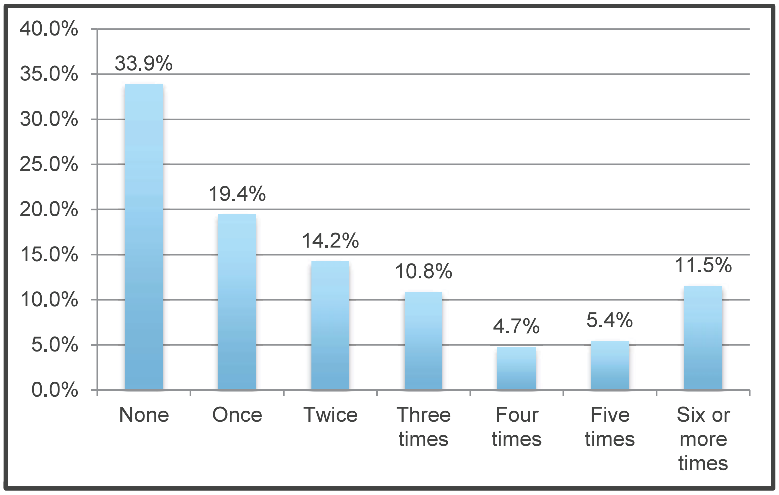

Q52A **NEW segment by time since diagnosis** How many times over your lifetime have you visited the emergency department or been admitted to the hospital due to cardiac symptoms?

	All patients		Less than 5 years		5 to less than 10 years		10 to less than 20 years		20 to less than 30 years		30 or more years	
**Response**	**Count**	**Percent**	**Count**	**Percent**	**Count**	**Percent**	**Count**	**Percent**	**Count**	**Percent**	**Count**	**Percent**
None	150	33.9%	46	41.4%	48	36.1%	39	33.6%	11	19.3%	5	21.7%
Once	86	19.4%	24	21.6%	25	18.8%	24	20.7%	9	15.8%	4	17.4%
Twice	63	14.2%	15	13.5%	20	15.0%	15	12.9%	12	21.1%	1	4.3%
Three times	48	10.8%	12	10.8%	15	11.3%	9	7.8%	7	12.3%	4	17.4%
Four times	21	4.7%	3	2.7%	8	6.0%	3	2.6%	4	7.0%	3	13.0%
Five times	24	5.4%	6	5.4%	7	5.3%	5	4.3%	4	7.0%	2	8.7%
Six or more times	51	11.5%	5	4.5%	10	7.5%	21	18.1%	10	17.5%	4	17.4%
Total	443		111		133		116		57		23	

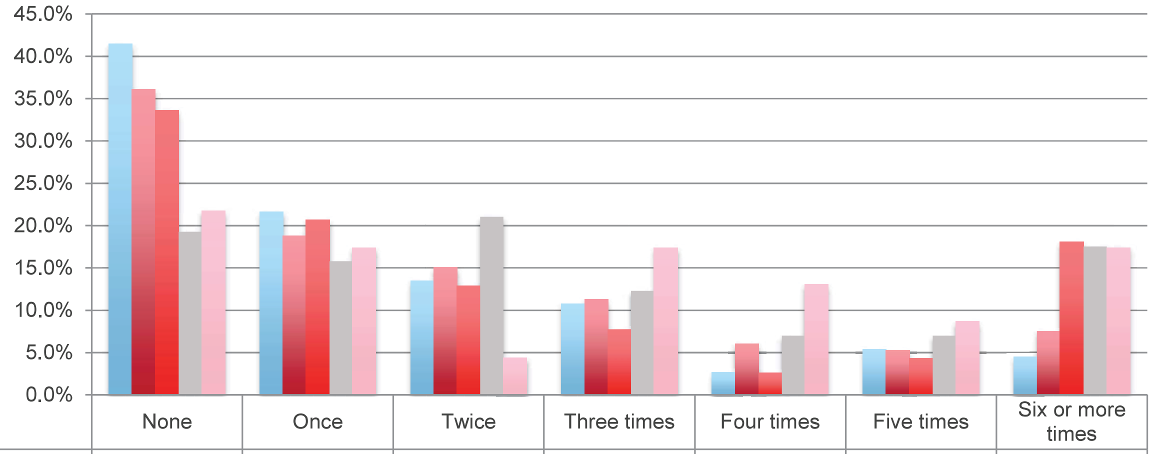

	None	Once	Twice	Three times	Four times	Five times	Six or more times
Less than 5 years	41.4%	21.6%	13.5%	10.8%	2.7%	5.4%	4.5%
5 to less than 10 years	36.1%	18.8%	15.0%	11.3%	6.0%	5.3%	7.5%
10 to less than 20 years	33.6%	20.7%	12.9%	7.8%	2.6%	4.3%	18.1%
20 to less than 30 years	19.3%	15.8%	21.1%	12.3%	7.0%	7.0%	17.5%
30 or more years	21.7%	17.4%	4.3%	17.4%	13.0%	8.7%	17.4%

Q52B **NEW segment by time since diagnosis** How many times over your lifetime have you visited the emergency department or been admitted to the hospital due to cardiac symptoms?

	None		Once		Twice		Three times		Four times		Five times		Six or more times	
**Time to Diagnosis**	**Count**	**Percent**	**Count**	**Percent**	**Count**	**Percent**	**Count**	**Percent**	**Count**	**Percent**	**Count**	**Percent**	**Count**	**Percent**
Less than 5 years	46	30.9%	24	27.9%	15	23.8%	12	25.5%	3	14.3%	6	25.0%	5	10.0%
5 to less than 10 years	48	32.2%	25	29.1%	20	31.7%	15	31.9%	8	38.1%	7	29.2%	10	20.0%
10 to less than 20 years	39	26.2%	24	27.9%	15	23.8%	9	19.1%	3	14.3%	5	20.8%	21	42.0%
20 to less than 30 years	11	7.4%	9	10.5%	12	19.0%	7	14.9%	4	19.0%	4	16.7%	10	20.0%
30 or more years	5	3.4%	4	4.7%	1	1.6%	4	8.5%	3	14.3%	2	8.3%	4	8.0%
Total	149		86		63		47		21		24		50	

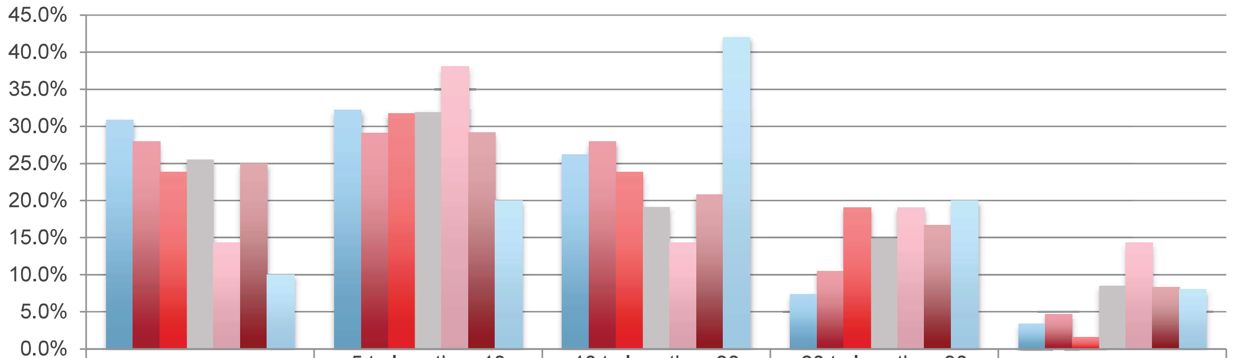

	Less than 5 years	5 to less than 10 years	10 to less than 20 years	20 to less than 30 years	30 or more years
None	30.9%	32.2%	26.2%	7.4%	3.4%
Once	27.9%	29.1%	27.9%	10.5%	4.7%
Twice	23.8%	31.7%	23.8%	19.0%	1.6%
Three times	25.5%	31.9%	19.1%	14.9%	8.5%
Four times	14.3%	38.1%	14.3%	19.0%	14.3%
Five times	25.0%	29.2%	20.8%	16.7%	8.3%
Six or more times	10.0%	20.0%	42.0%	20.0%	8.0%

Q52C NEW Segment by obstruction How many times over your lifetime have you visited the emergency department or been admitted to the hospital due to cardiac symptoms?

	All patients		Obstructive		Non-obstructive	
**Response**	**Count**	**Percent**	**Count**	**Percent**	**Count**	**Percent**
None	150	33.9%	70	27.3%	64	43.8%
Once	86	19.4%	50	19.5%	28	19.2%
Twice	63	14.2%	39	15.2%	18	12.3%
Three times	48	10.8%	34	13.3%	10	6.8%
Four times	21	4.7%	14	5.5%	5	3.4%
Five times	24	5.4%	16	6.3%	6	4.1%
Six or more times	51	11.5%	33	12.9%	15	10.3%
Total	443		256		146	

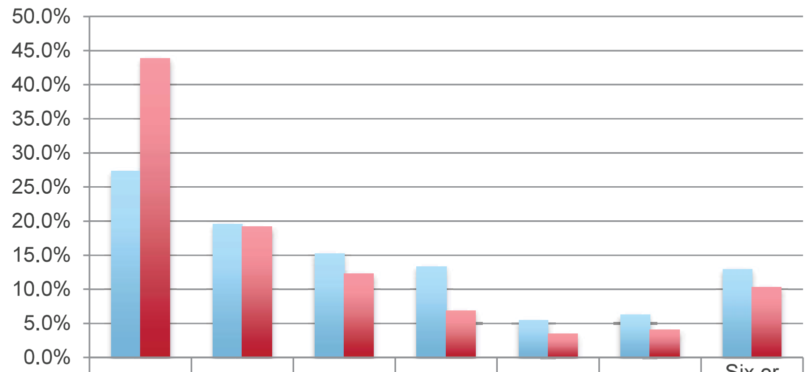

	None	Once	Twice	Three times	Four times	Five times	Six or more times
Obstructive	27.3%	19.5%	15.2%	13.3%	5.5%	6.3%	12.9%
Non-obstructive	43.8%	19.2%	12.3%	6.8%	3.4%	4.1%	10.3%

MyoKardia I CONFIDENTIAL
MyoKardia

Q53 **How many years has it been since your diagnosis of HCM?**

Response	Count	Percent
Less than 5 years	111	25.2%
5 to less than 10 years	134	30.4%
10 to less than 20 years	116	26.3%
20 to less than 30 years	57	12.9%
30 or more years	23	5.2%
Total	441	

Q54 **Have your symptoms increased or interfered with your life since your original diagnosis?**

Response	Count	Percent
Not at all	65	14.7%
Slightly	107	24.3%
Somewhat	138	31.3%
Significantly worsened	131	29.7%
Total	441	

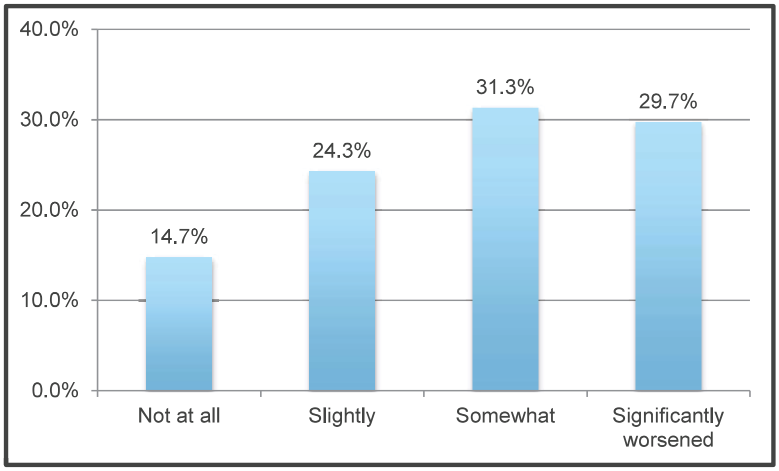

Q55 **In the past 4 weeks-- please rate your physical quality of life:**

Response	Count	Percent
Poor	20	4.5%
Fair	93	21.1%
Good	156	35.5%
Very good	119	27.0%
Excellent	52	11.8%
Total	440	

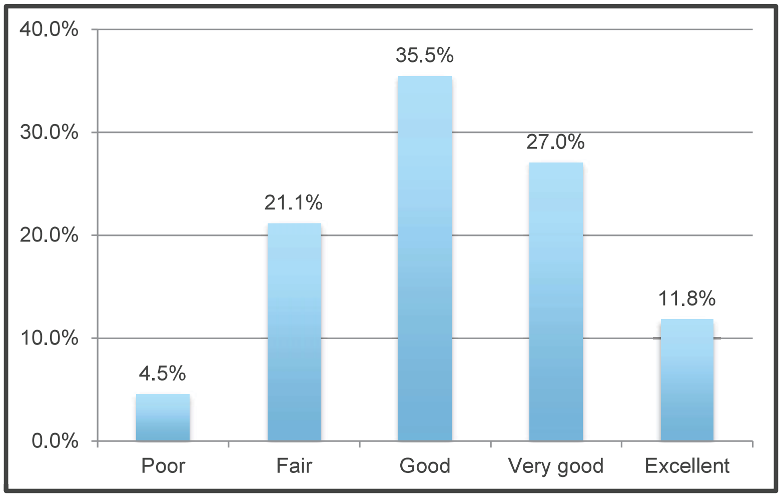

Q55A **NEW: Segment based on patient origination** In the past 4 weeks-- please rate your physical quality of life:

	All Patients		Symptoms		Unrelated Med Investig.		Screening	
**Response**	**Count**	**Percent**	**Count**	**Percent**	**Count**	**Percent**	**Count**	**Percent**
Poor	20	4.5%	15	6.4%	4	2.9%	1	1.5%
Fair	93	21.1%	66	28.0%	15	10.9%	12	17.9%
Good	156	35.5%	93	39.4%	42	30.7%	21	31.3%
Very good	119	27.0%	44	18.6%	55	40.1%	20	29.9%
Excellent	52	11.8%	18	7.6%	21	15.3%	13	19.4%
Total	440		236		137		67	

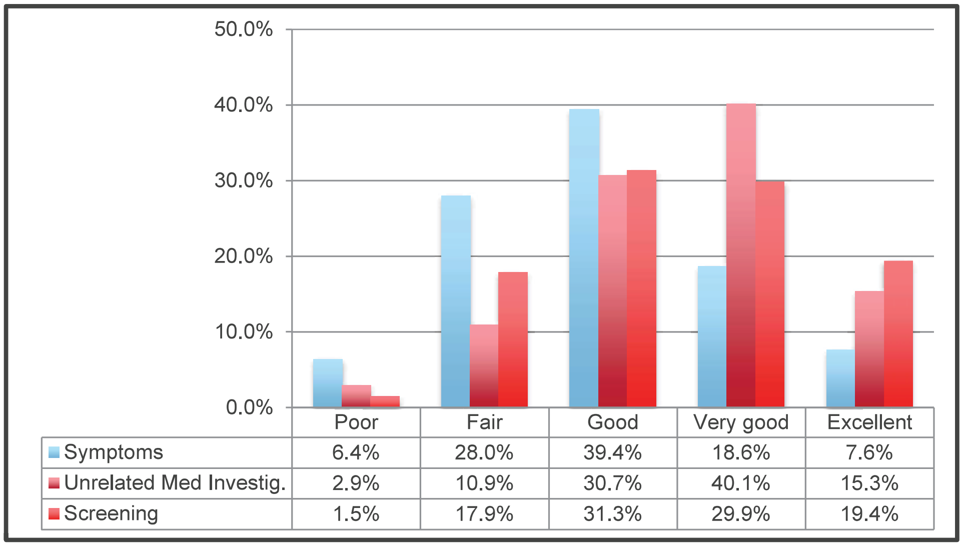

Q55B **NEW: Segment based on NYHA symptoms** In the past 4 weeks-- please rate your physical quality of life:

	All Patients		NYHA Class I		NYHA Class II		NYHA Class III		NYHA Class IV	
**Response**	**Count**	**Percent**	**Count**	**Percent**	**Count**	**Percent**	**Count**	**Percent**	**Count**	**Percent**
Poor	20	4.5%	0	0.0%	2	1.1%	8	6.0%	9	37.5%
Fair	93	21.1%	0	0.0%	31	17.0%	53	39.8%	7	29.2%
Good	156	35.5%	22	24.2%	81	44.5%	46	34.6%	4	16.7%
Very good	119	27.0%	34	37.4%	61	33.5%	21	15.8%	1	4.2%
Excellent	52	11.8%	35	38.5%	7	3.8%	5	3.8%	3	12.5%
Total	440		91		182		133		24	

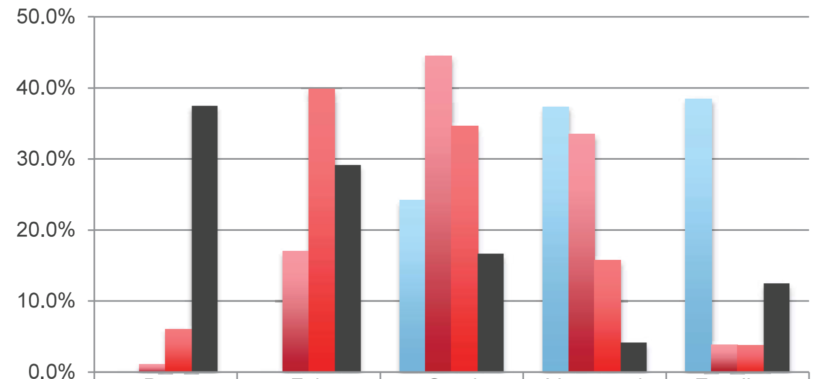

	Poor	Fair	Good	Very good	Excellent
NYHA Class I	0.0%	0.0%	24.2%	37.4%	38.5%
NYHA Class II	1.1%	17.0%	44.5%	33.5%	3.8%
NYHA Class III	6.0%	39.8%	34.6%	15.8%	3.8%
NYHA Class IV	37.5%	29.2%	16.7%	4.2%	12.5%

Q55C **NEW: Segment based on obstruction** In the past 4 weeks-- please rate your physical quality of life:

	All Patients		Obstructive		Non-obstructive	
**Response**	**Count**	**Percent**	**Count**	**Percent**	**Count**	**Percent**
Poor	20	4.5%	11	4.4%	6	4.1%
Fair	93	21.1%	58	23.0%	25	17.1%
Good	156	35.5%	106	42.1%	40	27.4%
Very good	119	27.0%	60	23.8%	44	30.1%
Excellent	52	11.8%	17	6.7%	31	21.2%
Total	440		252		146	

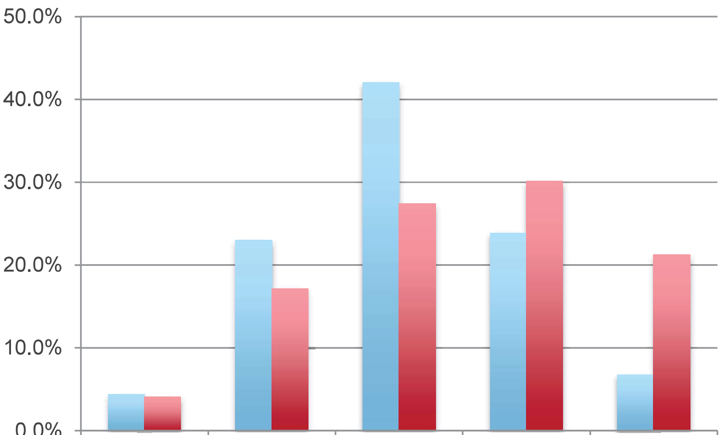

	Poor	Fair	Good	Very good	Excellent
Obstructive	4.4%	23.0%	42.1%	23.8%	6.7%
Non-obstructive	4.1%	17.1%	27.4%	30.1%	21.2%

Q56 **In the past 4 weeks-- please rate your emotional quality of life:**

Response	Count	Percent
Poor	26	5.9%
Fair	77	17.4%
Good	146	33.0%
Very good	126	28.5%
Excellent	67	15.2%
Total	442	

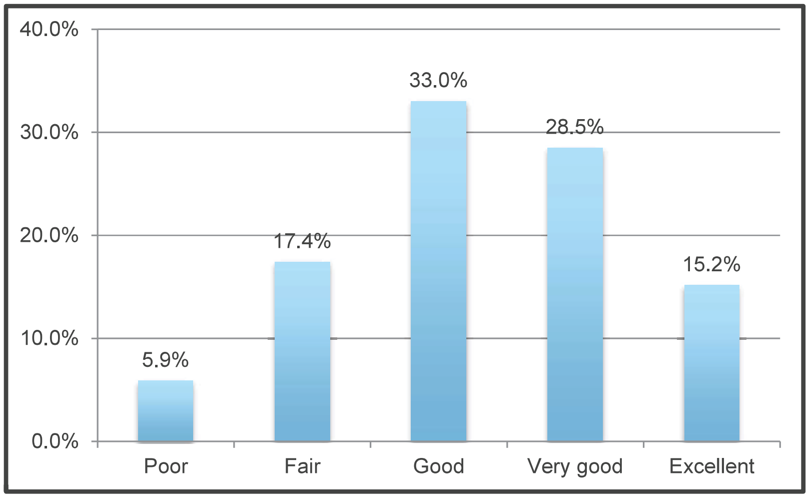

Q57 **To what extent does your HCM affect your work?**

Response	Count	Percent
Not at all	145	33.1%
Slightly	86	19.6%
Somewhat	74	16.9%
Significantly	35	8.0%
Unable to work	57	13.0%
Other	41	9.4%
Total	438	

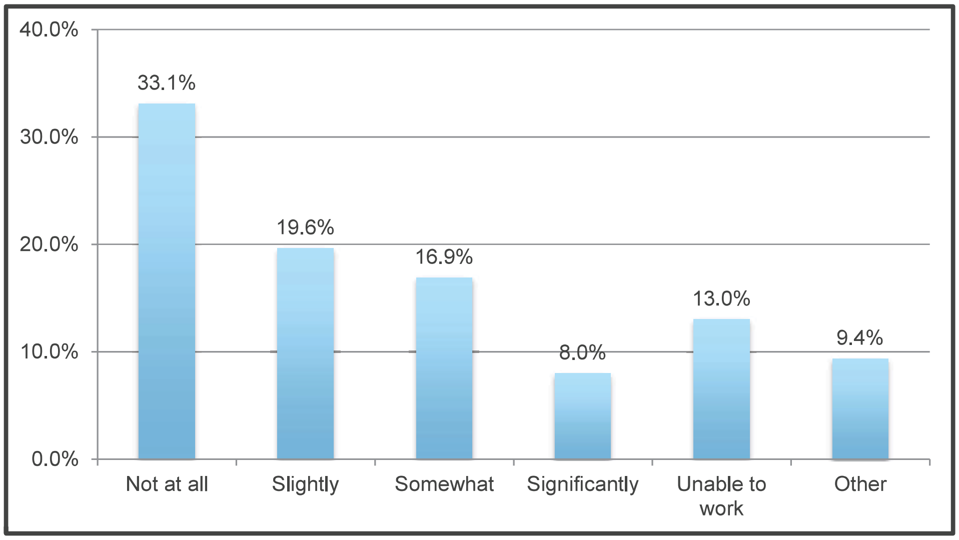

Q58 **To what extent does your HCM affect your social life?**

Response	Count	Percent
Not at all	147	33.7%
Slightly	107	24.5%
Somewhat	119	27.3%
Significantly	61	14.0%
Unable to socialize	2	0.5%
Total	436	

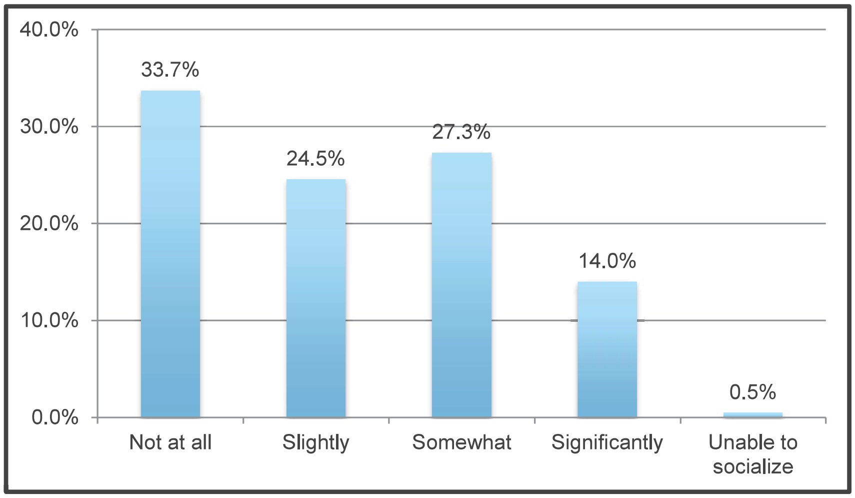

Q59 To what extent does your HCM affect your ability to exercise?

Response	Count	Percent
Not at all	49	11.2%
Slightly	84	19.2%
Somewhat	124	28.4%
Significantly	141	32.3%
Unable to exercise	39	8.9%
Total	437	

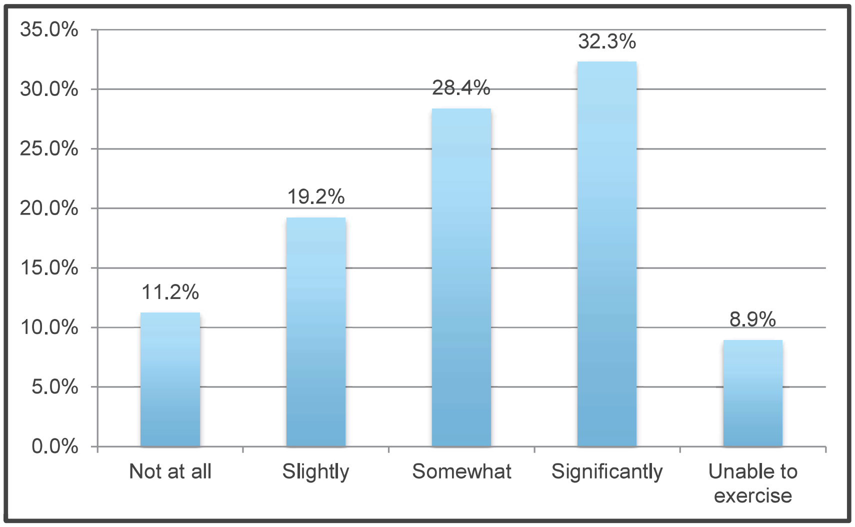

Q59A **NEW: Segment by patient origination** To what extent does your HCM affect your ability to exercise?

	All Patients		Symptoms		Unrelated Med Investigation		Screening	
**Response**	**Count**	**Percent**	**Count**	**Percent**	**Count**	**Percent**	**Count**	**Percent**
Not at all	49	11.2%	15	6.4%	19	14.0%	15	22.7%
Slightly	84	19.2%	34	14.5%	33	24.3%	17	25.8%
Somewhat	124	28.4%	69	29.4%	43	31.6%	12	18.2%
Significantly	141	32.3%	92	39.1%	31	22.8%	18	27.3%
Unable to exercise	39	8.9%	25	10.6%	10	7.4%	4	6.1%
Total	437		235		136		66	

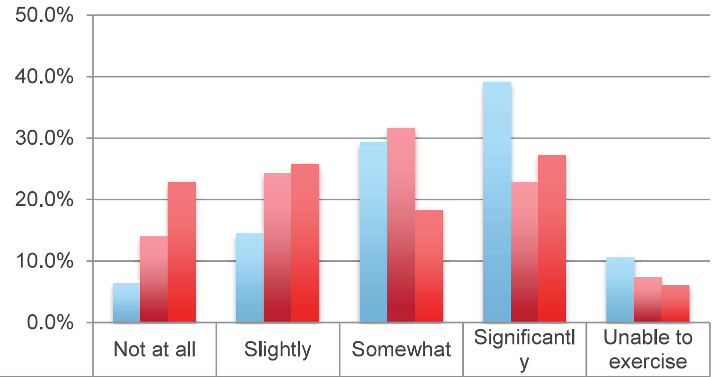

	Not at all	Slightly	Somewhat	Significantl y	Unable to exercise
Symptoms	6.4%	14.5%	29.4%	39.1%	10.6%
Unrelated Med Investigation	14.0%	24.3%	31.6%	22.8%	7.4%
Screening	22.7%	25.8%	18.2%	27.3%	6.1%

Q59B **NEW: Segment by NYHA Class** To what extent does your HCM affect your ability to exercise?

	All Patients		NYHA Class I		NYHA Class II		NYHA Class III		NYHA Class IV	
**Response**	**Count**	**Percent**	**Count**	**Percent**	**Count**	**Percent**	**Count**	**Percent**	**Count**	**Percent**
Not at all	49	11.2%	30	33.3%	8	4.4%	5	3.8%	3	12.5%
Slightly	84	19.2%	32	35.6%	45	24.9%	7	5.3%	0	0.0%
Somewhat	124	28.4%	23	25.6%	75	41.4%	21	15.8%	2	8.3%
Significantly	141	32.3%	5	5.6%	49	27.1%	82	61.7%	3	12.5%
Unable to exercise	39	8.9%	0	0.0%	4	2.2%	18	13.5%	16	66.7%
Total	437		90		181		133		24	

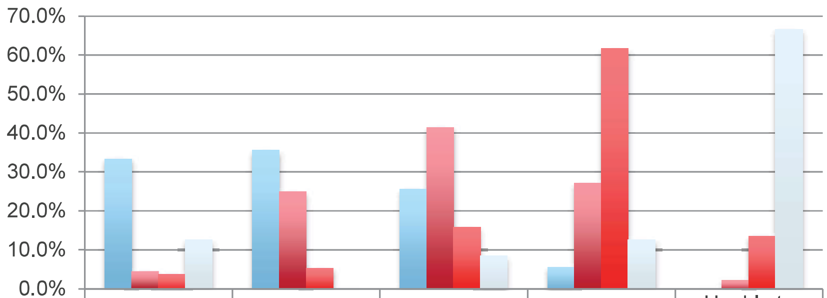

	Not at all	Slightly	Somewhat	Significantly	Unable to exercise
NYHA Class I	33.3%	35.6%	25.6%	5.6%	0.0%
NYHA Class II	4.4%	24.9%	41.4%	27.1%	2.2%
NYHA Class III	3.8%	5.3%	15.8%	61.7%	13.5%
NYHA Class IV	12.5%	0.0%	8.3%	12.5%	66.7%

Q60 **To what extent does your HCM affect your ability to travel?**

Response	Count	Percent
Not at all	203	46.3%
Slightly	96	21.9%
Somewhat	97	22.1%
Significantly	40	9.1%
Unable to travel	2	0.5%
Total	438	

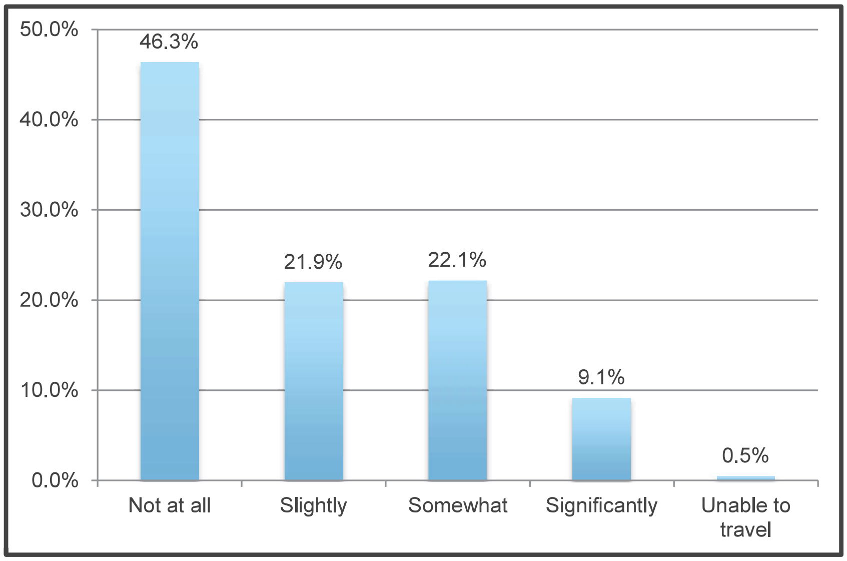

Q61 To what extent does your HCM affect physical relationships?

Response	Count	Percent
Not at all	195	44.8%
Slightly	98	22.5%
Somewhat	82	18.9%
Significantly	51	11.7%
Unable to maintain a physical relationship	9	2.1%
Total	435	

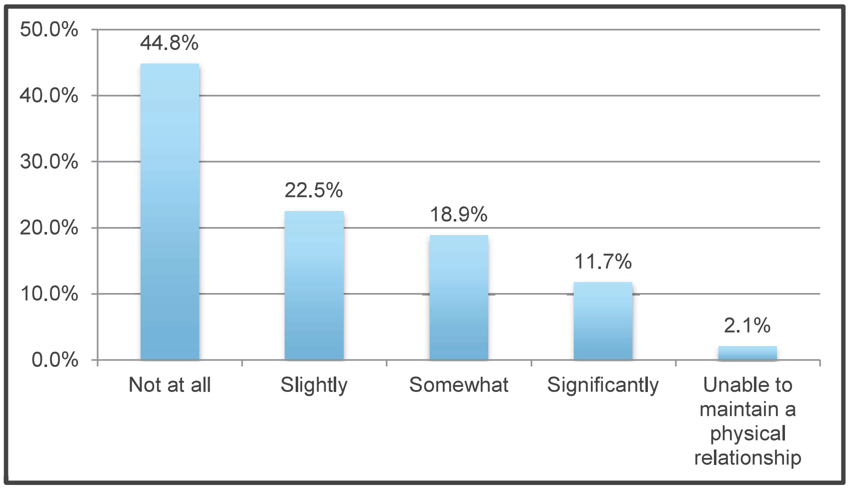

Q62 **How aware is your HCM specialist of the burden of your HCM on your overall quality of life?**

Response	Count	Percent
Not at all aware	53	12.5%
Slightly aware	58	13.6%
Somewhat aware	85	20.0%
Very aware	146	34.4%
I don't see an HCM specialist	83	19.5%
Total	425	

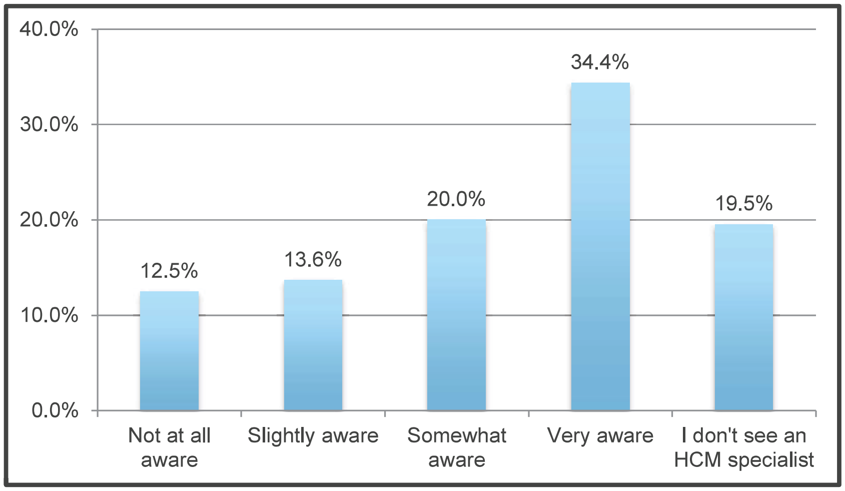

Q63 **How aware is your general cardiologist of the burden of your HCM on your overall quality of life?**

Response	Count	Percent
Not at all aware	57	13.4%
Slightly aware	71	16.7%
Somewhat aware	79	18.6%
Very aware	127	30.0%
I don't see a general cardiologist	90	21.2%
Total	424	

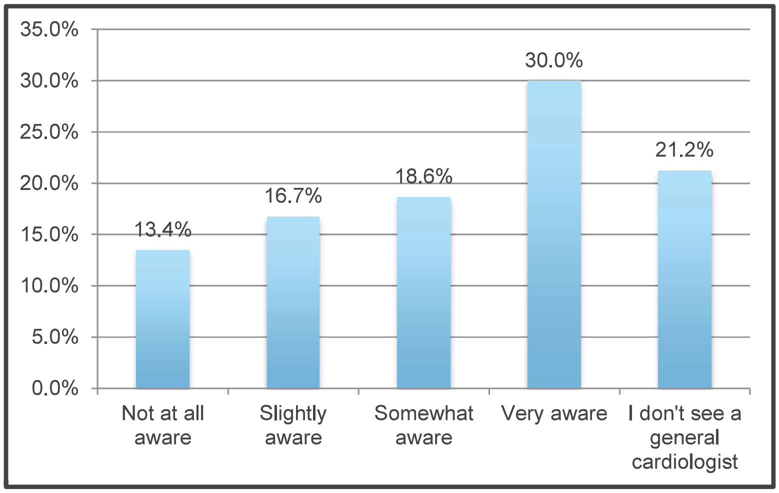

Q64 **Excluding your HCM specialist-- how well do you feel other physicians in your life understand your HCM medical condition?**

Response	Count	Percent
Poorly	55	12.5%
Not that well	125	28.5%
Average	127	28.9%
Reasonably well	94	21.4%
Very well	38	8.7%
Total	439	

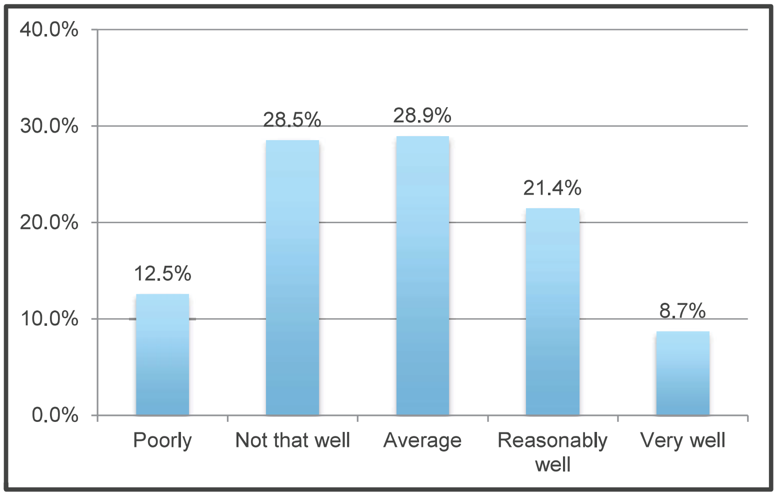

Q65 **Beyond family members-- and excluding social media contacts-- how many people do you personally know with HCM?**

Response	Count	Percent
None	277	63.0%
1 to 4 people	141	32.0%
5 to 9 people	17	3.9%
10 or more people	5	1.1%
Total	440	

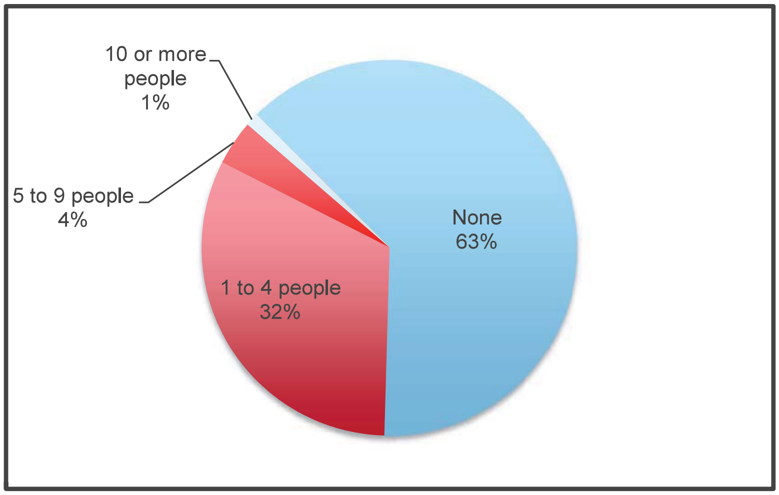

Q66 **How engaged are you with HCM in seeking out information-- discussing concerns with your physician-- talking about HCM with your family-- or participating in patient advocacy?**

Response	Count	Percent
No engagement	57	12.9%
Mildly engaged	172	38.9%
Moderately engaged	119	26.9%
Highly engaged	76	17.2%
Extremely highly engaged	18	4.1%
Total	442	

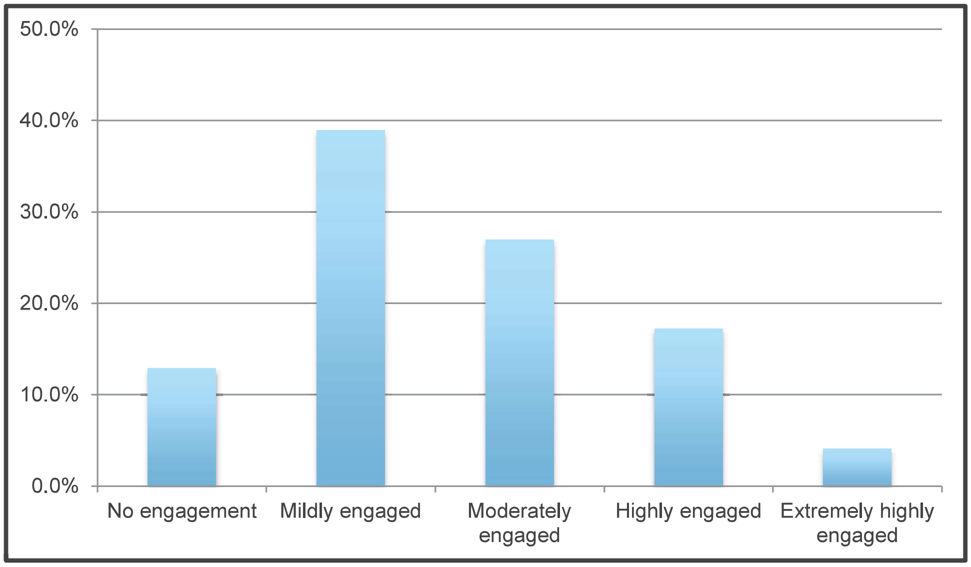

Q67 **What level of education and awareness do you believe you have about HCM including symptoms-- causes-- complications-- tests and treatment options?**

Response	Count	Percent
Poor	13	2.9%
Fair	54	12.2%
Good	131	29.6%
Very good	166	37.6%
Excellent	78	17.6%
Total	442	

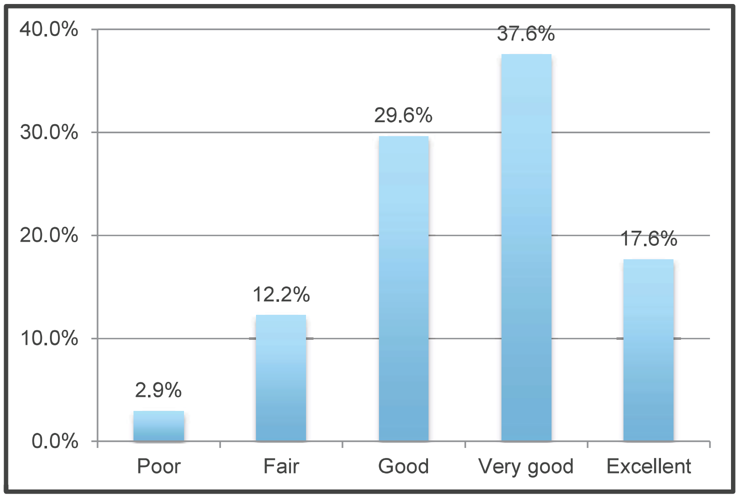

Q68 **Where do you obtain your information about HCM? (Check all that apply)**

Response	Count	Percent
HCMA	369	83.1%
Your HCM physician	302	68.0%
Other	130	29.3%
Local doctor	89	20.0%
Facebook	61	13.7%
Friends with HCM	31	7.0%
Total (N): all respondents	444	

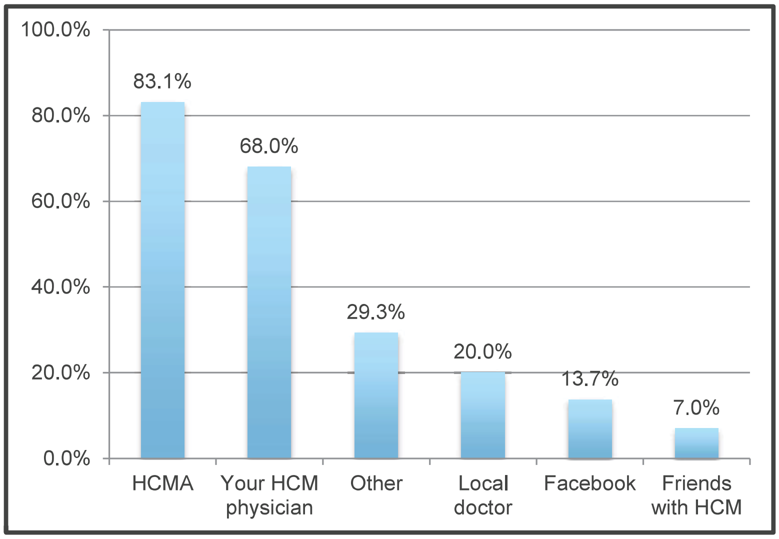

Q69 **Have you ever attended an educational program on HCM? (Check all that apply)**

Response	Count	Percent
Yes-- I have attended an HCMA event	108	24.3%
Yes-- I have attended an HCM educational program	58	13.1%
No-- I have not-- nor do I think I would	83	18.7%
No-- I have not but may be interested in doing so	218	49.1%
Total (N): all respondents	444	

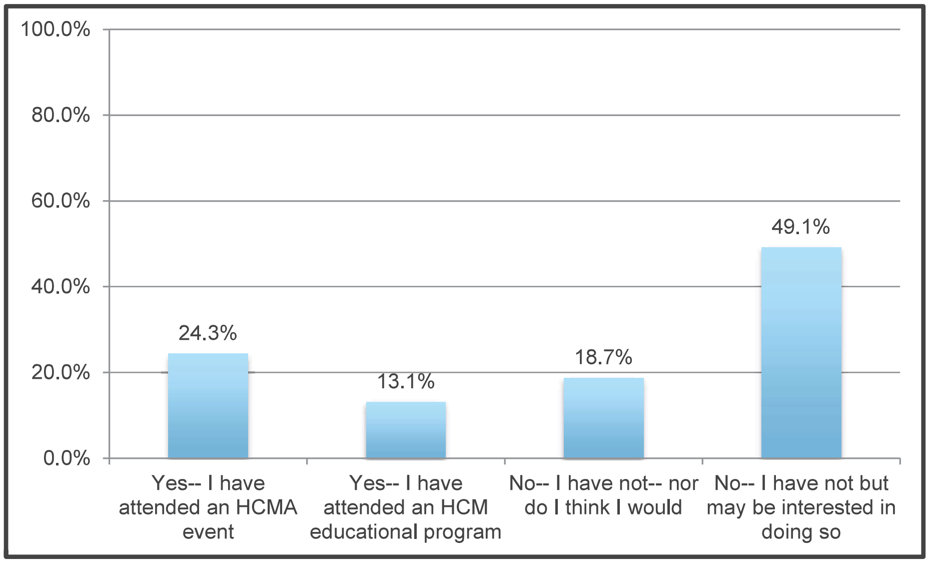

Q70 **Have you ever participated in a clinical trial?**

Response	Count	Percent
Yes	89	20.3%
No	349	79.7%
Total	438	

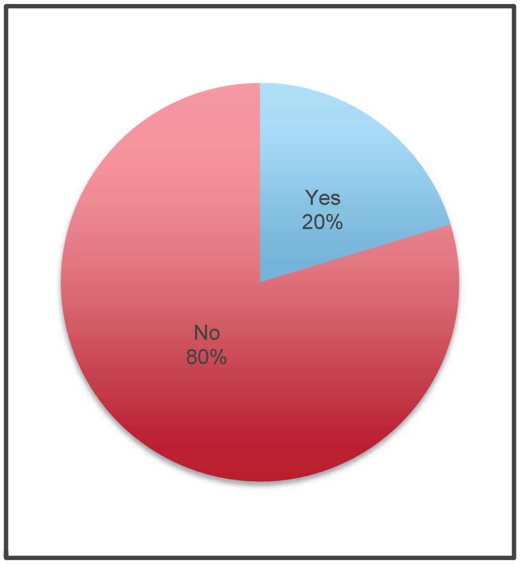

Q71 **If yes-- why did you participate? (Check all that apply)**

Response	Count	Percent
Desire to help advance medicine/science	70	78.7%
My physician recommended it	35	39.3%
Other	12	13.5%
Financial incentive	2	2.2%
Total (N): all respondents participating in a trial	89	

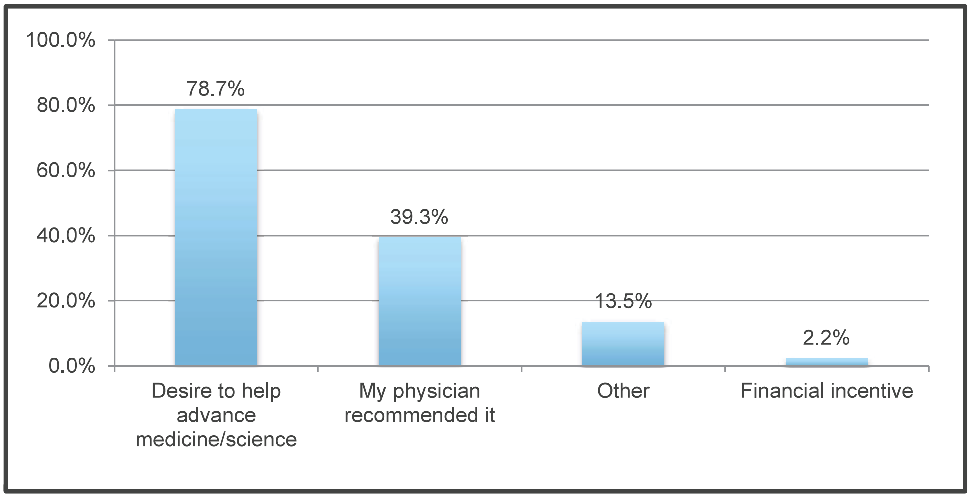

Q72 **Would you consider participating in a clinical trial for a new HCM therapy?**

Response	Count	Percent
Yes	315	72.2%
No	121	27.8%
Total	436	

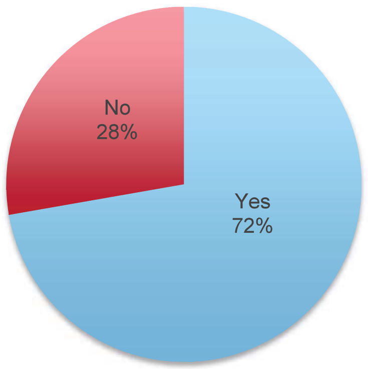

Q73 **You replied that you are not interested in participating in clinical trials-- can you please provide us with your reasons for not being interested? Check all that apply**

Response	Count	Percent
Concern about safety or side effects	54	44.6%
The benefit to me is not clear or adequate	51	42.1%
Other	35	28.9%
Concern about the distance to travel to the study site	27	22.3%
Concern about receiving a placebo in the trial	25	20.7%
I believe my treatment is better than that being studied	19	15.7%
I do not have time	18	14.9%
I do not trust companies	6	5.0%
Total (N): all those not interested in participating in a trial	121	

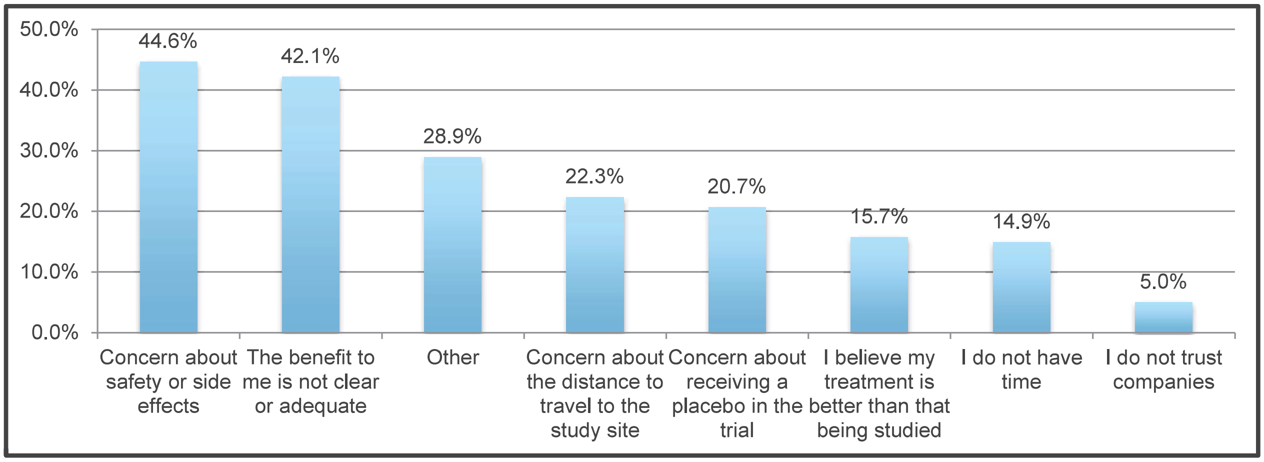

Q74 **What gender are you?**

Response	Count	Percent
Male	227	51.8%
Female	211	48.2%
Total	438	

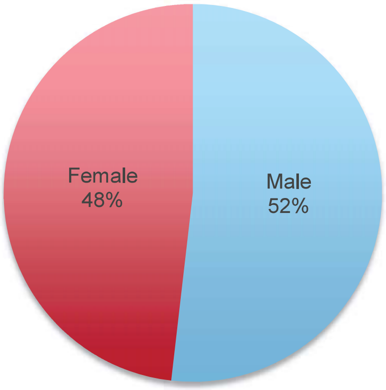

Q75 **What age group are you?**

Response	Count	Percent
<18 years old	16	3.6%
18-25	8	1.8%
26-34	18	4.1%
35-44	61	13.8%
45-54	89	20.2%
55-64	143	32.4%
65-74	83	18.8%
75+ years old	23	5.2%
Total	441	

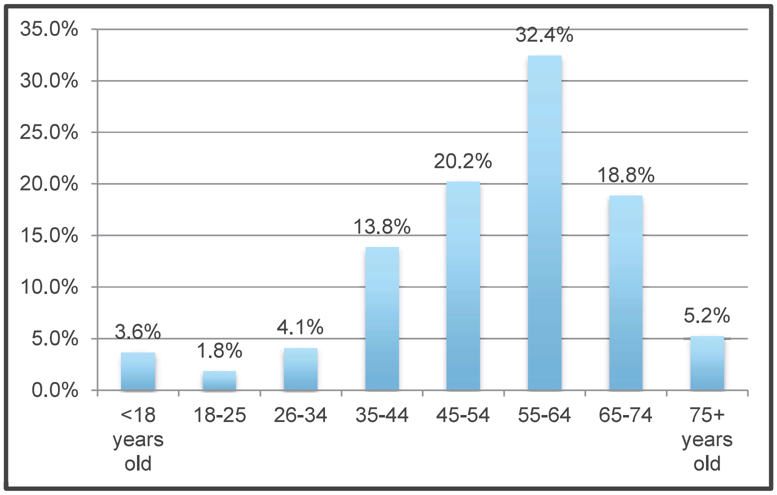

Q76 **What is your ethnicity?**

Response	Count	Percent
Caucasian	411	92.6%
Hispanic	8	1.8%
African American	6	1.4%
Native American	5	1.1%
Asian	8	1.8%
Other	6	1.4%
Total	444	

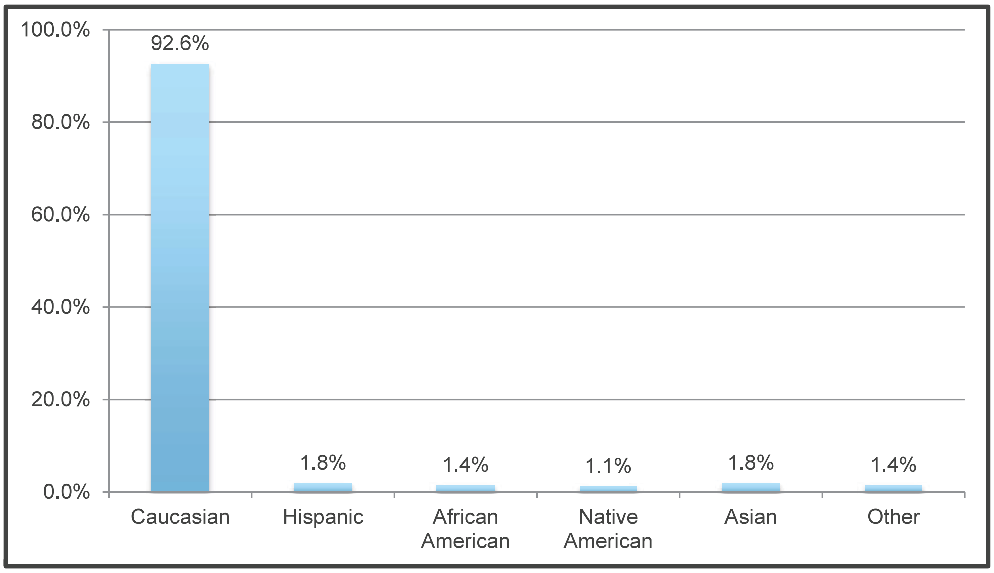

Q77 **How many children do you have?**

Response	Count	Percent
None	104	23.7%
1	63	14.4%
2	167	38.0%
3	73	16.6%
4	18	4.1%
5	8	1.8%
6 or more	6	1.4%
Total	439	

Q78 **Are you covered by health insurance?**

Response	Count	Percent
Yes	421	95.5%
No	5	1.1%
I live outside the USA - and am covered by a governmental insurance program	15	3.4%
Total	441	

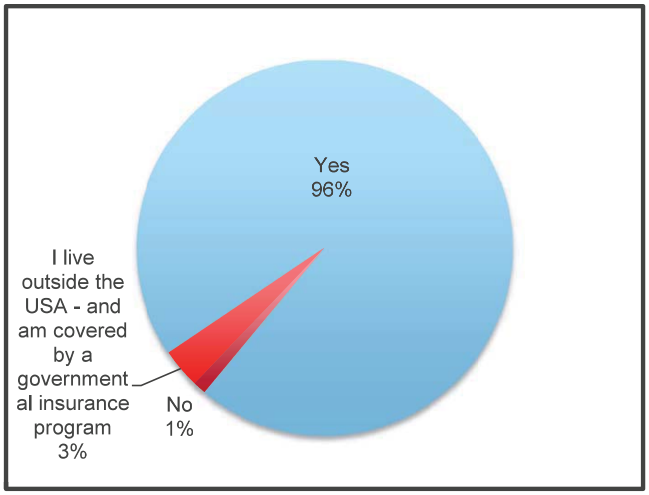

Q79 **If yes-- is the coverage?**

Response	Count	Percent
Provided by your or your spouse's employer	208	49.2%
Private insurance (you pay the entire premium)	58	13.7%
State Program	12	2.8%
Medicare	18	4.3%
Medicare and supplement	92	21.7%
I have 2 forms of insurance (both my spouse and I have coverage)	12	2.8%
Other	23	5.4%
Total	423	

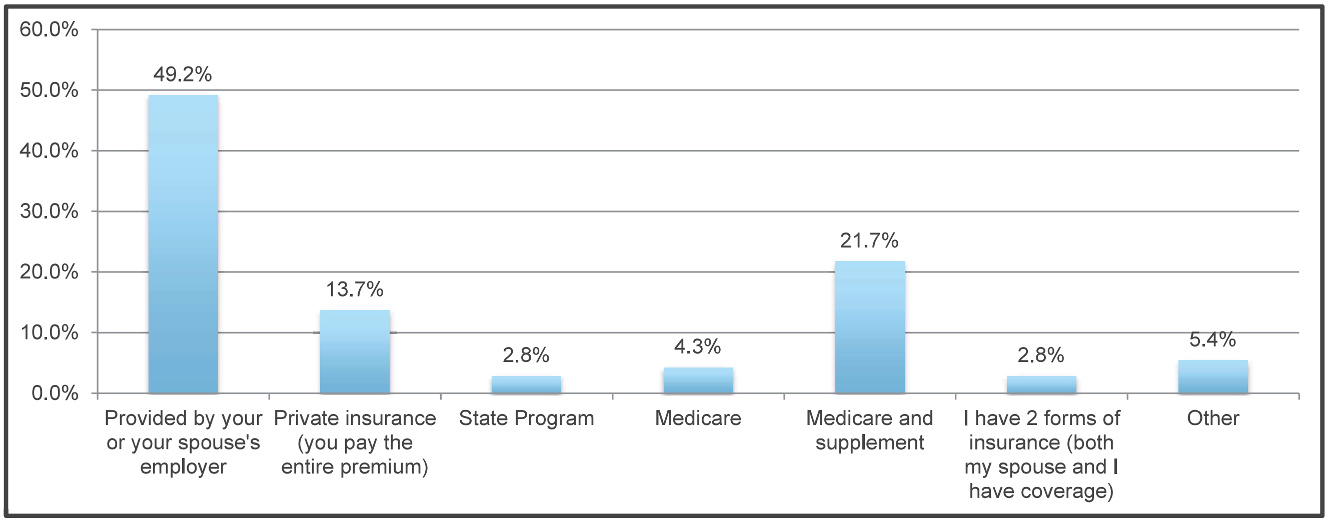

Q80 **Thank you for your participation in this survey. Are you interested in any of the following? (Check all that apply)**

Response	Count	Percent
Further sharing your experiences of being an HCM patient	265	59.7%
Learning more about participating in clinical trials	210	47.3%
Total (N): all respondents	444	

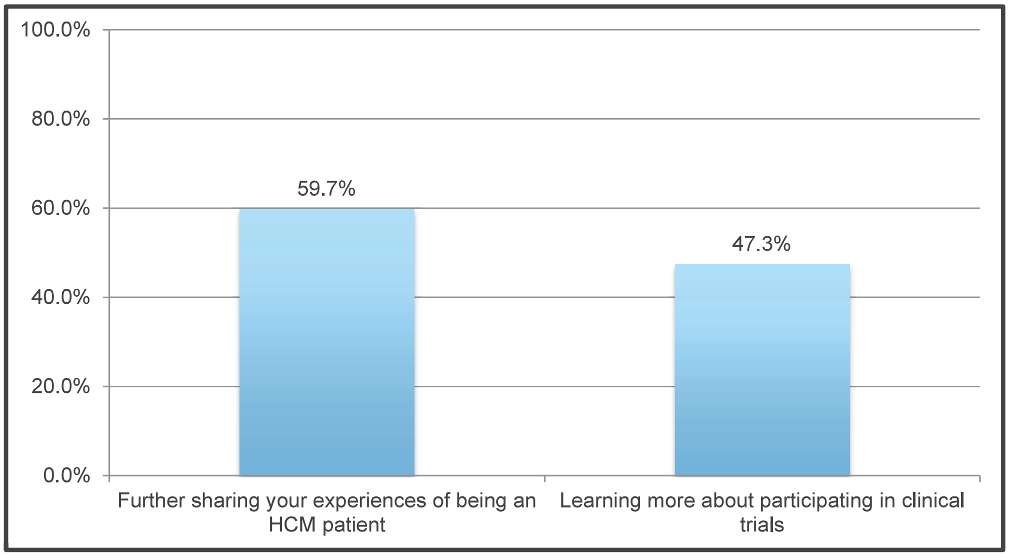

Supplement: Supplementary file 1 — Additional file 1. [file 41687_2020_269_MOESM1_ESM.pdf]
