# Supplementary material for: Patient experiences with hypertrophic cardiomyopathy: a conceptual model of symptoms and impacts on quality of life
Source: J Patient Rep Outcomes. 2020 Dec 1;4:102. doi: 10.1186/s41687-020-00269-8 (PMC7708573; doi:10.1186/s41687-020-00269-8)

Symptoms in Patients with HCM:

A Literature Review of the Existing Evidence on the Symptoms of HCM

The key objective of the literature review is to gather information on symptoms and impacts of HCM (and specifically to identify if any symptoms are different between non-obstructive HCM and obstructive HCM).

Methods

Published literature review

A search for published articles about HCM diagnosis or treatment was conducted in EMBASE (Table S1) and PubMED (Table S2); relevant English language articles were reviewed for information on HCM-specific symptoms and impacts.

Published articles were subjected to two levels of review. Level 1 review involved screening all titles and abstracts by keywords produced by the searches for relevancy according to pre-determined eligibility criteria. Full-text papers were obtained for studies considered potentially relevant to the review, or where uncertainty existed as to whether a paper should be included. Level 2 review involved a formal assessment of all full-text papers retrieved for relevance and inclusion into the review. Studies that met all inclusion and exclusion criteria after full-text screening were included in the final set of studies.

Review Eligibility Criteria

Published articles were included in the review if they met the following inclusion criteria:

1. The population under investigation was composed of adults (≥18 years of age) with HCM, obstructed HCM, heart failure, or cardiomyopathy.
2. The study included details related to symptoms, symptom impacts, effects of therapies on symptoms, quality of life, or PRO measures.

Exclusion of published articles was guided by the following predetermined criteria:

1. Non-relevant populations
2. No mention of symptoms associated with HCM; or no mention of symptom impacts (e.g., emotional, social, psychological, physical); or no inclusion of a PRO measure
3. Non-empirical studies/unspecified methodology (e.g., letters, general review

papers/discussion papers, descriptive narratives, and commentaries)

4. Non-English language

5. Non-human study or in vitro study

Table S1: Search Terms HCM (EMBASE 6 December 2016)

No.	Query	Results
#1	'hypertrophic cardiomyopathy'/exp OR 'hypertrophic cardiomyopathy' OR (hypertrophic:ab,ti AND cardiomyopathy:ab,ti) OR (hcm:ab,ti AND cardiomypath*:ab,ti) OR 'genetic cardiomyopathy':ab,ti OR 'hereditary cardiomyopathy':ab,ti	25685
#2	'pro measure*' OR 'patient reported outcome*':ab,ti OR 'treatment outcome'/exp OR ('self reported':ab,ti AND outcome*:ab,ti) OR ('self evaluation'/exp AND outcome*:ab,ti AND (measure*:ab,ti OR assess*:ab,ti)) OR ('patient reported' AND outcome*:ab,ti AND measure*:ab AND ti) OR ('patient preference'/exp AND (outcome*:ti OR outcome*:de)) OR proms OR patient* NEAR/2 reported OR patient* NEAR/2 rating OR patient* NEAR/2 rated	1291363
#3	'daily life activity'/exp OR 'activities of daily living':ab,ti OR adl:ab,ti OR 'adl disability'/de OR 'activity of daily living assessment'/exp OR activit* NEAR/3 'daily living'	91949
#4	'quality of life'/exp OR 'quality of life':ab,ti OR qol:ab,ti OR hrqol:ab,ti OR (quality:ab,ti AND ('well being':ab,ti OR life:ab,ti))	449303
#5	#1 AND (#2 OR #3 OR #4)	1841
#6	#1 AND (#2 OR #3 OR #4) AND [2015-2016]/py	330
#7	'editorial'/exp OR 'erratum'/exp OR 'letter'/exp OR 'note'/exp	2215863
#8	#6 NOT #7	299
#9	#8 AND ([article]/lim OR [article in press]/lim OR [review]/lim OR [short survey]/lim)	254

Table S2: Search Terms HCM (PubMed 6 December 2016)

Search	Query	Results
#1	Search ("Cardiomyopathy, Hypertrophic"[Mesh] OR "Hypertrophic cardiomyopathy"[tw] OR (Hypertrophic[tiab] AND cardiomyopathy[tiab]) OR (HCM[tiab] AND cardiomyopath*[tiab]) OR "genetic cardiomyopathy"[tiab] OR "hereditary cardiomyopathy"[tiab])	17332
#2	Search ((pro[tiab] measure*[tiab]) OR "patient reported outcome*"[tiab] OR ("self reported"[tiab] AND outcome*[tiab]) OR ("Diagnostic Self Evaluation"[Mesh] AND outcome*[tiab]) OR ("self evaluation"[tiab] AND outcome*[tiab]) OR ("Self-Assessment"[MeSH Major Topic] AND outcome*[tiab]) OR ("patient reported"[tw] AND (outcome*[ti] OR outcome*[ot])) OR Proms[tw]) OR "Patient Outcome Assessment"[Majr] OR "self report"[tiab] OR "self management"[tiab] OR "self care"[tiab])	105429
#3	Search ("Quality of Life"[Mesh] OR "Quality of Life"[Text Word] OR qol[Text Word] OR hrqol[Text Word] OR (quality[tiab] AND "well being"[tiab]))	245189
#4	Search (("activities of daily living"[MeSH Terms] OR "activities of daily living"[Text Word] OR adl[Text Word]) OR (activit*[Text Word] AND daily living[Text Word]))	70125
#5	Search (#1 AND (#2 OR #3 OR #4))	167
#6	Search ("editorial"[Publication Type]) OR "letter"[Publication Type]) OR "comment"[Publication Type]) OR "published erratum"[Publication Type]) OR "news"[Publication Type])	1704538
#7	Search (#5 NOT #6)	165
#8	Search (#7) AND ("2015"[Date - Publication] : "2016"[Date - Publication])	28

Review of Guidelines

Current HCM guidelines were reviewed for symptoms specifically related to HCM or oHCM. This review included the 2014 European Society of Cardiology (ESC) Guidelines on Diagnosis and Management of Hypertrophic Cardiomyopathy and the 2011 Cardiac Society of Australia and New Zealand (CSANZ) Guidelines for Diagnosis and Management of Hypertrophic Cardiomyopathy. These guidelines were also searched for any mention of relevant outcomes from a patient perspective or measures for assessing symptoms of HCM.

Patient Advocate Websites

In addition to the guidelines review, we researched patient advocacy websites (*The Hypertrophic Cardiomyopathy Association* (www.4hcm.org) and *Cardiomyopathy UK* (www.cardiomyopathy.org/)*)* to identify symptoms and impacts of HCM. The symptoms and impacts reported in the websites were cross-referenced with the literature search, and a final list of suggested HCM symptoms and impacts was generated.

Figure 1. Flow Diagram of Search Hits and Inclusion Numbers

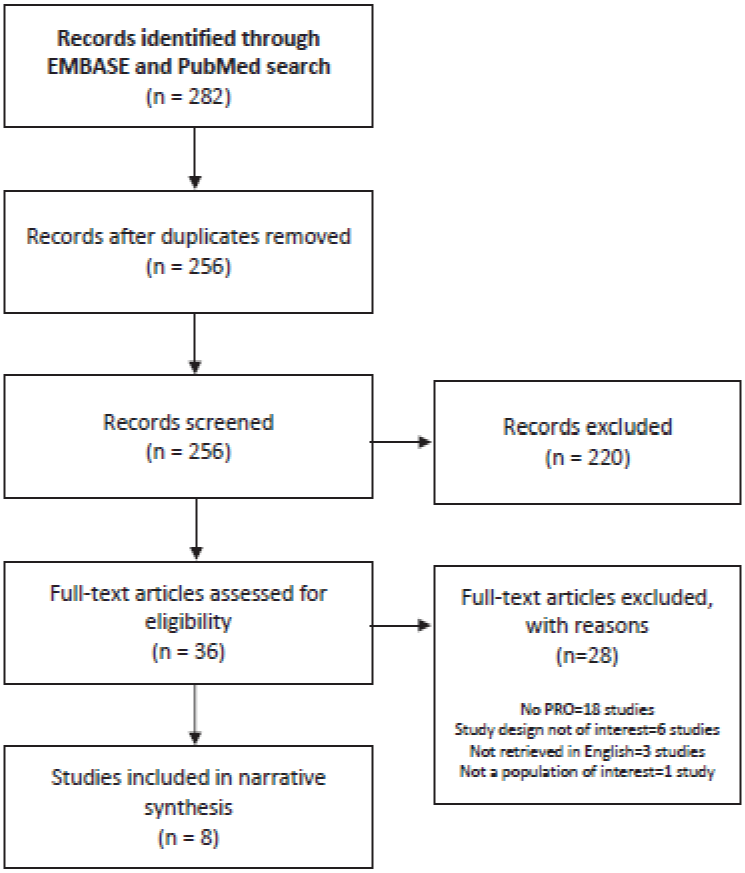

Supplement: Supplementary file 2 — Additional file 2. [file 41687_2020_269_MOESM2_ESM.pdf]
